# Climate change influences on the potential distribution of *Dianthus polylepis* Bien. ex Boiss. (Caryophyllaceae), an endemic species in the Irano-Turanian region

**DOI:** 10.1371/journal.pone.0237527

**Published:** 2020-08-18

**Authors:** Maryam Behroozian, Hamid Ejtehadi, A. Townsend Peterson, Farshid Memariani, Mansour Mesdaghi

**Affiliations:** 1 Quantitative Plant Ecology and Biodiversity Research Lab., Department of Biology, Faculty of Science, Ferdowsi University of Mashhad, Mashhad, Iran; 2 Biodiversity Institute, University of Kansas, Lawrence, Kansas, United States of America; 3 Department of Botany, Research Center for Plant Science, Ferdowsi University of Mashhad, Mashhad, Iran; 4 Department of Range and Watershed Management, Faculty of Natural Resources and Environment, Ferdowsi University of Mashhad, Mashhad, Iran; Instituto Federal de Educacao Ciencia e Tecnologia Goiano - Campus Urutai, BRAZIL

## Abstract

Endemic and restricted-range species are considered to be particularly vulnerable to the effects of environmental change, which makes assessing likely climate change effects on geographic distributions of such species important to the development of integrated conservation strategies. Here, we determined distributional patterns for an endemic species of *Dianthus* (*Dianthus polylepis*) in the Irano-Turanian region using a maximum-entropy algorithm. In total, 70 occurrence points and 19 climatic variables were used to estimate the potential distributional area under current conditions and two future representative concentration pathway (RCP2.6 and RCP8.5) scenarios under seven general circulation models for 2050. Mean diurnal range, iso-thermality, minimum temperature of coldest quarter, and annual precipitation were major factors that appeared to structure the distribution of the species. Most current potential suitable areas were located in montane regions. Model transfers to future-climate scenarios displayed upward shifts in elevation and northward shifts geographically for the species. Our results can be used to define high-priority areas in the Irano-Turanian region for conservation management plans for this species and can offer a template for analyses of other endangered and threatened species in the region.

## Introduction

Climate change is known as a major threat to species survival and the integrity of ecosystems worldwide [1,2]. Numerous studies have documented the effects of climate change on biodiversity during the twentieth century, including alterations in distribution and phenology, and increases in extinction risk for many species [3–5]. Given these changes, assessments of the impacts of climate change on species’ geographic distributions are necessary to reveal the extent of risk and to permit the design of appropriate conservation programs for species.

Endemic and restricted-range species are considered to be particularly vulnerable to the effects of environmental change, which makes assessing likely climate change effects on geographic distributions of such species important to the development of integrated conservation strategies [6]. Among major effects of climate change on species is that of shifting their potential distributions [7,8], threatening their viability via range reductions [9–11], and altering the representation of species in protected areas [12]. Climate change may also lead to local extinctions, ultimately reducing range size, and increasing habitat destruction and fragmentation; as a consequence, such that climate change particularly threatens species that already have small ranges [12–14]. These implications will be particularly damning for endemic plant species in montane areas, because of their restricted geographic ranges and relatively narrow environmental tolerances [15,16]. These species may shift their geographic ranges to higher elevations in the face of a warming climate, often accompanied by decreases in population size via range reduction and fragmentation [7,17]. Despite the importance of these regions for preserving endangered and endemic species, relatively few studies have focused on their climate change sensitivity [18]. Hence, developing the ability to predict the responses of restricted-range, montane species to climate change is important for assessing extinction risk or climate adaptation [19,20].

One approach by which to evaluate potential impacts of climate change on species’ distributions is via the use of ecological niche models (ENMs) [21,22]. These models relate occurrence data and environmental variables using statistical or machine-learning procedures to create an empirical, correlational model of the fundamental ecological niche [21,23]. One can then transfer modeled environmental requirements of species to future climate scenarios, predicting their potential distributions under changing conditions [21,24]. Among the different tools used in ENM, Maxent is a popular choice, is appropriate in situations in which only presence data are available, and achieves powerful predictive performance [25,26].

We selected an endemic species of the genus *Dianthus* L. (Caryophyllaceae) as a case study of the influence of climate change on the geographic potential of species in the Irano-Turanian region using ENM. The genus *Dianthus* is distributed throughout Eurasia and Africa, with more than 300 species. Over half of its species occur in small, geographically restricted ranges [27,28]. Previous studies have highlighted that climatic preferences have played prominent roles in the evolution and diversification of *Dianthus* [27–30]. In addition, most *Dianthus* species in the Irano-Turanian region live in rocky mountain habitats that may be affected severely by changing environmental conditions, land-use changes, and human activities. Therefore, many such montane *Dianthus* species, given their restricted ranges, may be highly sensitive to climate change.

*Dianthus polylepis* Bien. ex Boiss. 1867 is a species endemic to the mountains of northeastern Iran and southwestern Turkmenistan, with a highly restricted geographic distribution at elevations of 1100–2500 m, including the Kopet Dagh, Hezar-Msjed, Binalood, Sabzevar, and Kashmar-Torbat mountains [31–33]. Although it is listed by IUCN as Least Concern (LC) [34] based on its relatively wide distribution [31], the species is distributed patchily and occurs in small, isolated, and scattered populations, often with low genetic diversity [35,36]—this contrast of a broad extent of occurrence with a small actual distribution has important implications for conservation. Populations of the species are impacted severely by disturbance, so identifying the potential geographic distribution of this species and predicting how climate change will affect its geographic range can be useful for its conversation and management. Previous studies have focused on phylogeny and taxonomic status [37–39], yet little is known as regards the details of its geographic distribution or the environmental factors associated with that distribution.

In this study, we investigate the impacts of climate change on the potential distribution of *D*. *polylepis* across the Irano-Turanian region, under current and future climatic conditions. Our aims were: (a) to estimate the potential distribution of *D*. *polylepis* under current climatic conditions, (b) to identify key environmental factors delimiting that range, and (c) to predict the potential distribution of this species under various climate change scenarios.

## Materials and methods

### Study area and occurrence data

**Fig 1:**
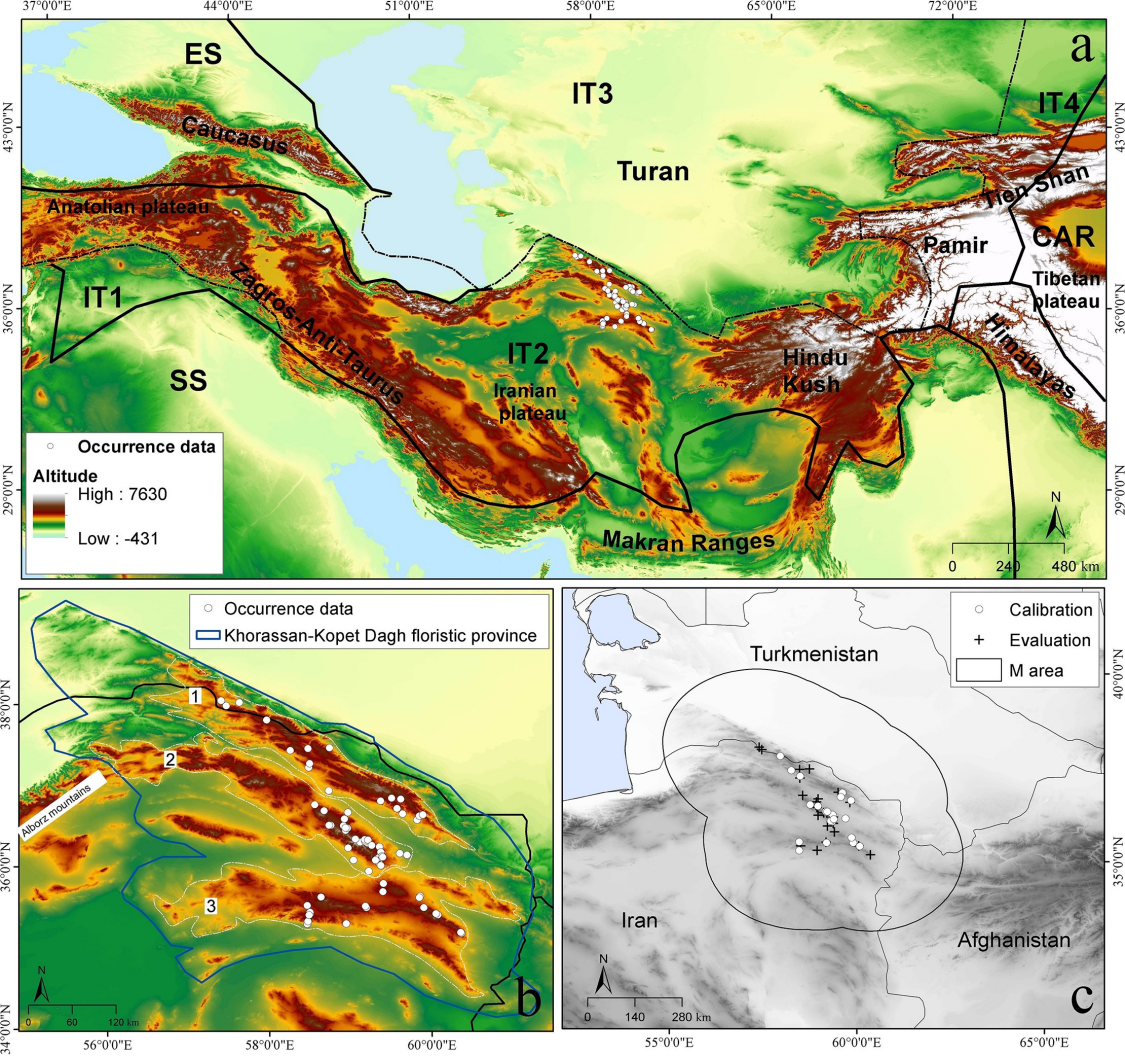
Study area and distribution of *Dianthus polylepis*. (a) Phytogeographic subdivisions of the Irano-Turanian region modified by the authors from previous published maps [47]. (b) The distribution of *Dianthus polylepis* in three main mountain ranges including (1) Kopet Dagh and Hezar-Masjed (3106 m), (2) Eastern Alborz (2956 m) and Binalood (3301 m), (3) Sabzevar (2937 m) and Kashmar-Torbat (2940 m) mountains. (c) Occurrence records of *Dianthus polylepis* and accessible area (**M**) for model calibration.

This study focused on the Irano-Turanian region (IT region) in southwestern Asia [40]. (Fig 1A). The region is a species- and endemism-rich floristic region, particularly in the Iranian Plateau and Anatolian Plateau [41–44]. It contains four sub-regions (IT1, IT2, IT3, IT4); the sub-region IT2 encompasses a wide area in Iran, Turkey, and Afghanistan, including many *Dianthus* species. This sub-region has a distinct climate, as well as the highest diversity of physiognomic types, including forest, scrub, alpine, and sub-alpine grassland and steppe, montane steppe, desert steppe, and diverse halophytic communities. Hence, the IT2 is considered as the most significant center of plant speciation and endemism in the Irano-Turanian floristic region [40,42,43,45,46].

*Dianthus polylepis* is distributed in the Khorassan-Kopet Dagh floristic province (KK), a small area within IT2 northeastern Iran and southwestern Turkmenistan [31,48]. The three most prominent mountain ranges are the Kopet Dagh (peak 2676 m) and Hezar-Msjed (3106 m) mountains, eastern Alborz (2956 m) and Binalood (3301 m) mountains, and the Sabzevar (2937 m) and Kashmar-Torbat (2940 m) ranges (Fig 1B). The climate in the area is distinctly continental: mean annual precipitation is 300–380 mm in the high mountains, and annual mean temperature varies 12–19°C, depending on elevation [49]. Under the Bioclimatic Classification System [50], the Kopet Dagh mountains have a Mediterranean or Irano-Turanian xeric-continental climate, with a short summer dry period. A limited area between the Kopeh Dagh and Aladagh-Binaloud mountains has a shorter summer drought and higher annual precipitation. *Dianthus polylepis* is distributed broadly in montane steppe habitats in this area. We used occurrence data for *D*. *polylepis* from field surveys at sites across the region [37,39] and gathered additional records from previous studies, in the form of herbarium specimens in the collections of the Ferdowsi University of Mashhad Herbarium (FUMH) and Herbarium of Research Institute of Forests and Rangelands (TARI). Additional georeferenced distribution records for this species were extracted from the literature, which was checked via searches on the scientific name in Google Scholar, although the number of such studies is few; we ended up using only information from two older papers that provided locality information [32,33] (S1 Table). We checked the Global Biodiversity Information Facility (GBIF; http://www.gbif.org/) and SpeciesLink (http://splink.cria.org.br/) and found no additional occurrence records.

To assure acceptable data quality in the occurrence information, we filtered occurrence data in two steps. That is, we checked occurrence data carefully for localities falling outside the species’ known range, and removed records for which coordinate uncertainty was either too high or was lacking. To reduce autocorrelation among occurrence data and potential for overfitting [24], we eliminated one of each pair of records falling within single grid cells (~1 km) using the spThin library in R Statistical package 3.5.1 [51]. Final occurrence data were split randomly into two equal portions: 50% with which to calibrate the model and the other 50% to evaluate model predictions (Fig 1C).

### Climate data

Given the focus of this paper on estimating the future potential distribution of the species, we were constrained to variables for which “before and after” versions of those variables were available, which is to say, a set of climate variables that have been developed for the present and multiple future scenarios. Current climatic data layers (19 initially) were therefore obtained from WorldClim version 1.4 (http://www.worldclim.org) [52], at a spatial resolution of 30” (~1 km). We excluded four of the bioclimatic variables (bio8, bio9, bio18, and bio19) from all analyses because they show odd spatial anomalies in the form of discontinuities between neighboring pixels in the absence of environmental gradients on the ground [53,54]. Two procedures were used to select subsets of the remaining 15 bioclimatic variables. First, we examined Pearson correlation coefficients among the variables across the calibration area (see below) using R 3.5.0 software [55], and found 5 variables among which all correlation coefficients were <0.8, a value that indicates that the two variables share 60+% of their overall variance; in each case of highly correlated variable pairs (i.e., *r* > 0.8), we chose the variable with the clearest interpretation regarding the biology of the species for further analysis [50,56]. With that reduced set of five variables, we selected three sets of variables, chosen in sequential steps using the jackknife function in Maxent, removing the variable with the lowest independent contributions, creating sets of 5, 4, and 3 variables for analysis.

For climate change projections, we downloaded climatic variables under future-climate scenarios to match the present-day datasets from the Climate Change, Agriculture, and Food Security website (CCAFS; http://www.ccafs-climate.org) [57], also at a spatial resolution of 30”. We selected seven general circulation models (GCMs) for 2050, under two representative concentration pathway scenarios (RCP 4.5 and RCP 8.5), giving lower and upper views of possible future greenhouse gas emissions, respectively, and bracketing likely future conditions. GCMs employed in this study included (1) Beijing Climate Center, China Meteorological Administration (BCC-CSM 1–1); (2) Canadian Center for Climate Modeling and Analysis (CCCMA-CANESMA); (3) Climate System Model (CSIRO Mk3); (4) NASA Goddard Institute for Space Studies, USA (GISS-E2-R); (5) Institute for Numerical Mathematics, Russia (INMCM4); (6) Institute Pierre-Simon Laplace, France (IPSL-CM4); and (7) Meteorological Research Institute, Japan (MRI-CGCM3). We used this diverse set of GCMs because estimates of GCM-to-GCM variation are key in understanding uncertainty in predictions of future distribution potential of species [58].

### Ecological niche modeling

Ecological niche models were created using a maximum entropy algorithm [23]. Although many have advocated for exploring multiple such algorithms for fitting ecological niche models [59,60], in light of different types of probabilities estimated by different algorithms [21], we have opted to use only Maxent, but with a deep exploration of parameter space [61]. We developed a specific hypothesis of the area that has been accessible to the species over relevant periods [62]. This area, termed **M**, was estimated as the area within 2° (~220 km) of known occurrences, which was delineated using buffer routines in ArcGIS 10.3.1, and was inspected to assure that all sites within the area are likely within the dispersal reach of the species. We delineated **M** using buffer routines in ArcMAP (version 3.10)—in light of the limited dispersal capacity of *D*. *polylepis*, we used a 220 km buffer around known presence points as a proxy of this area [63]. In light of the limited latitudinal range of this species, we used geographic coordinates for our analyses (WGS 1984); however, all area calculations were developed in a cylindrical equal-area projection.

We used a detailed model selection procedure [64] via the kuenm package in R [61]. We explored a combination of 20 regularization parameter values (0.1–1.0 at intervals of 0.1; 1–2 at intervals of 0.25; 2–6 at intervals of 1; 8; and 10), 29 sets of model response types (i.e., all possible combinations of linear, quadratic, product, threshold, and hinge responses), and the three sets of environmental variables described above. In all, 1740 candidate models were created, and each was evaluated based on statistical significance (partial ROC tests) [65], omission rate (OR), and the Akaike information criterion corrected for small sample sizes (AICc) [64].

In greater detail, candidate models were evaluated using partial ROC tests applied to 500 random replicate samples of 50% of the occurrences left out of model calibration [65], and statistical significance evaluated via a direct count of replicates with AUC ratios ≤1.0. All models were thresholded based on an acceptable calibration omission rate [65] of *E* = 7%, and removed models with omission rates above 0.07. Finally, we filtered models to retain only models with the lowest values of the Akaike information criterion (AICc) [64], retaining models with AICc values within 2 units of the minimum. Final models were selected based on (1) statistical significance, (2) acceptably low omission rates, and (3) presenting the minimum value of AICc, to assure that we worked with relatively simple but highly predictive models [61].

Final models were created using parameter settings selected in kuenm, with 10 bootstrap replicate analyses among available occurrence data. We transferred final models across a broader area of southwestern Asia under present-day conditions; models were also transferred to future conditions, permitting extrapolation and clamping under conditions not manifested across the calibration area. We used median values across replicates for the final models as the best estimate of the suitability across the region for present-day conditions, and assessed uncertainty as the range (maximum-minimum) across the 10 replicate analyses for each final parameter set. For projections to future conditions, the median of replicate medians was calculated across all 7 GCMs for each RCP, and the range among replicates was again used as an index of uncertainty related to the availability of occurrence data. We converted raw suitability scores into binary estimates of potential presence and absence across regions via establishing the highest threshold level using an adjusted minimum training presence threshold based on *E* = 0.07 [66], which in effect allows for some amount of error in the occurrence data. We visualized future expected changes in distributional patterns in terms of the agreement or lack of agreement among model transfers to different GCM outputs.

## Results

**Fig 2:**
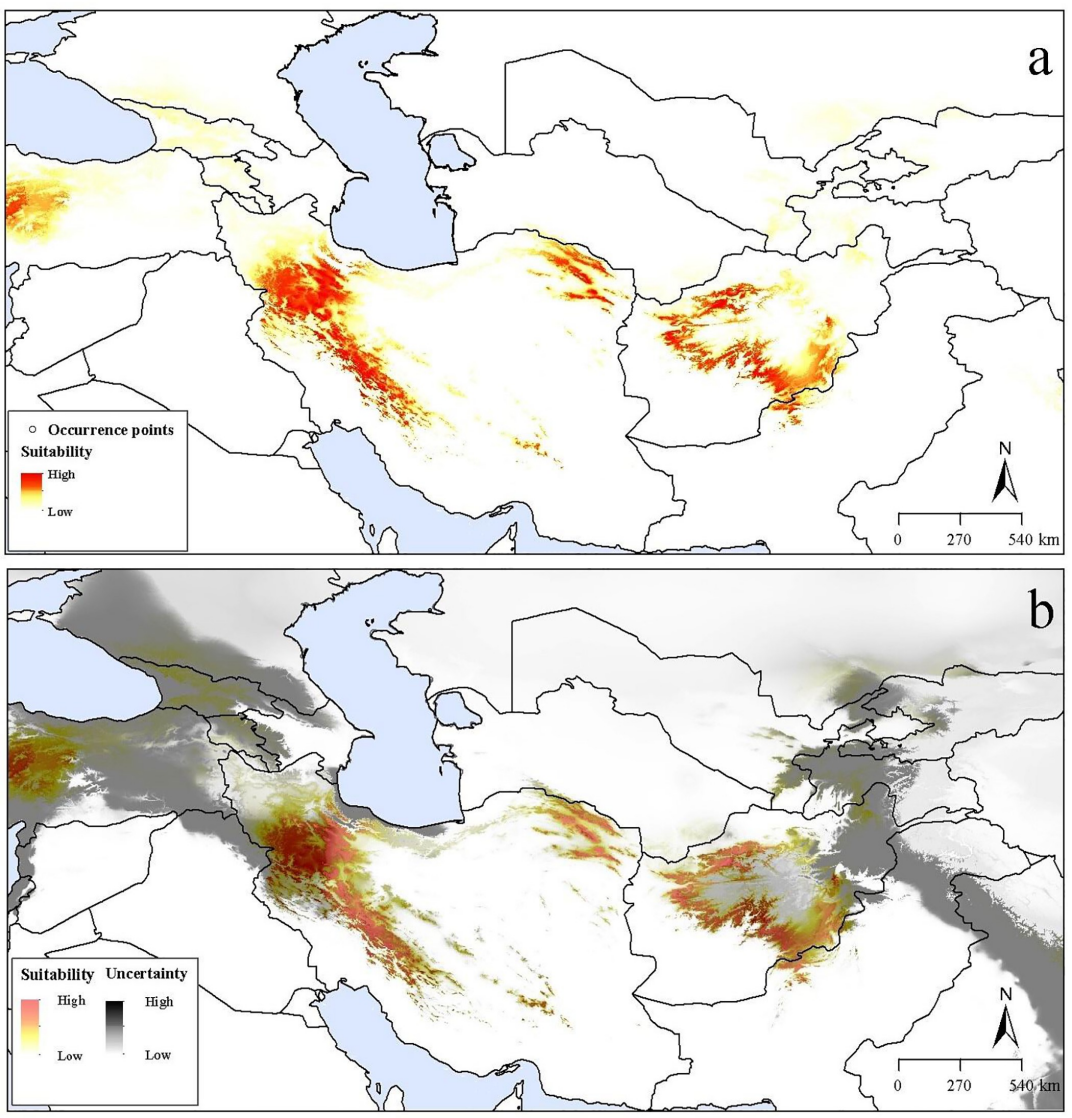
Predicted current suitable regions for *Dianthus polylepis* distribution across the Iran-Turanian region. (a) Median prediction. (b) The uncertainty associated with the predicted suitable regions.

In all, 83 unique occurrences for *D*. *polylepis* were assembled from across the study area. Two of these records did not include sufficiently precise information and were removed. To avoid spatial autocorrelation in occurrence data, 11 of 81 records were also excluded based on ~1 km distance filtering. In all, then, 70 unique occurrences were used for calibration and evaluation (Fig 2; S1 Table), which represents the full set of existing data regarding the occurrence of the species.

Based on the correlation matrix among environmental variables (*r* > 0.8), climatic variables bio1, bio4, bio5, bio6, bio7, bio10, bio13, bio14, bio15, and bio16, were removed from the analysis. The remaining five variables were subjected to the jackknife analysis, including mean diurnal range (bio2), Iso-thermality (bio3), mean temperature of the coldest quarter (bio11), annual precipitation (bio12), and precipitation of the driest quarter (bio17). In two jackknife steps, bioclimatic variables 17 and 3 were the variables that contributed least. Hence, we explored three sets of environmental variables: bio2-bio11-bio12, bio 2-bio11-bio12-bio17, and bio2-bio3-bio11-bio12-bio17.

In all, 1740 models were evaluated for model calibration, most of which were significantly better than random expectation based on the partial ROC test (*P* < 0.001; see S2 Table). Model selection procedures indicated that the models with the best fit to our data in terms of significant, low-omission models and models with the lowest AICc value, comprised two models including quadratic-threshold-hinge or all feature classes, a regularization parameter value of 1.75, and the intermediate set of four environmental variables. Because AICc values of other models were higher, these two models were used for estimating potential distributions of *D*. *polylepis* under present and future conditions.

Ecological niche models based on present-day conditions indicated high suitability for *D*. *polylepis* across montane areas, including parts of Iran, Afghanistan, and Turkey, and a small part of Pakistan. In Iran, the highest suitability was observed in the Zagros range in the west and several mountain ranges in the east, whereas suitability was markedly lower in central areas comprising desert and low-elevation areas. Suitable areas were also identified in the Hindu Kush in central Afghanistan and parts of the western Anatolian Plateau in Turkey (Fig 2A). Under present-day conditions, relatively low levels of uncertainty were observed across the predicted distributional areas, except in small parts of western Iran and western Turkey. Although these areas show low levels of uncertainty, some sites (e.g., in Turkey) are likely not accessible to the species currently in light of long distances to known distributional areas and broad, human-modified intervening habitats. High uncertainty was in western and northern Iran, much of Turkey, Armenia, Georgia, southern Russia, eastern Afghanistan, eastern Pakistan, western India, and other parts of Central Asia (Fig 2B). Therefore, models identified suitable distributional areas with high confidence only in eastern and western Iran and central Afghanistan.

**Fig 3:**
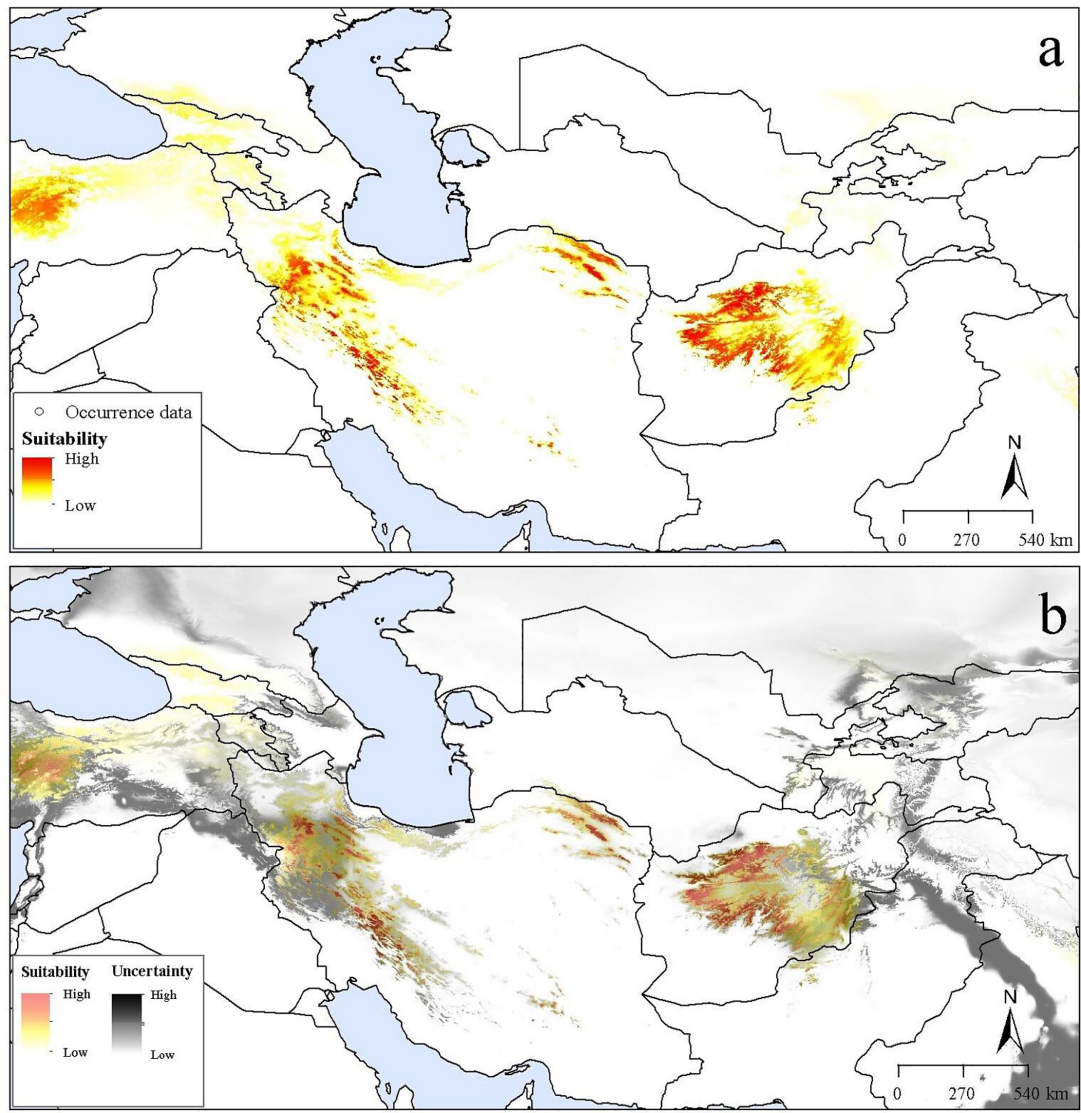
Potential future distribution of *Dianthus polylepis* under the Representative Concentration Pathway (RCP) 4.5 across the Iran-Turanian region. (a) Median prediction. (b) The uncertainty associated with the predicted suitable regions.

**Fig 4:**
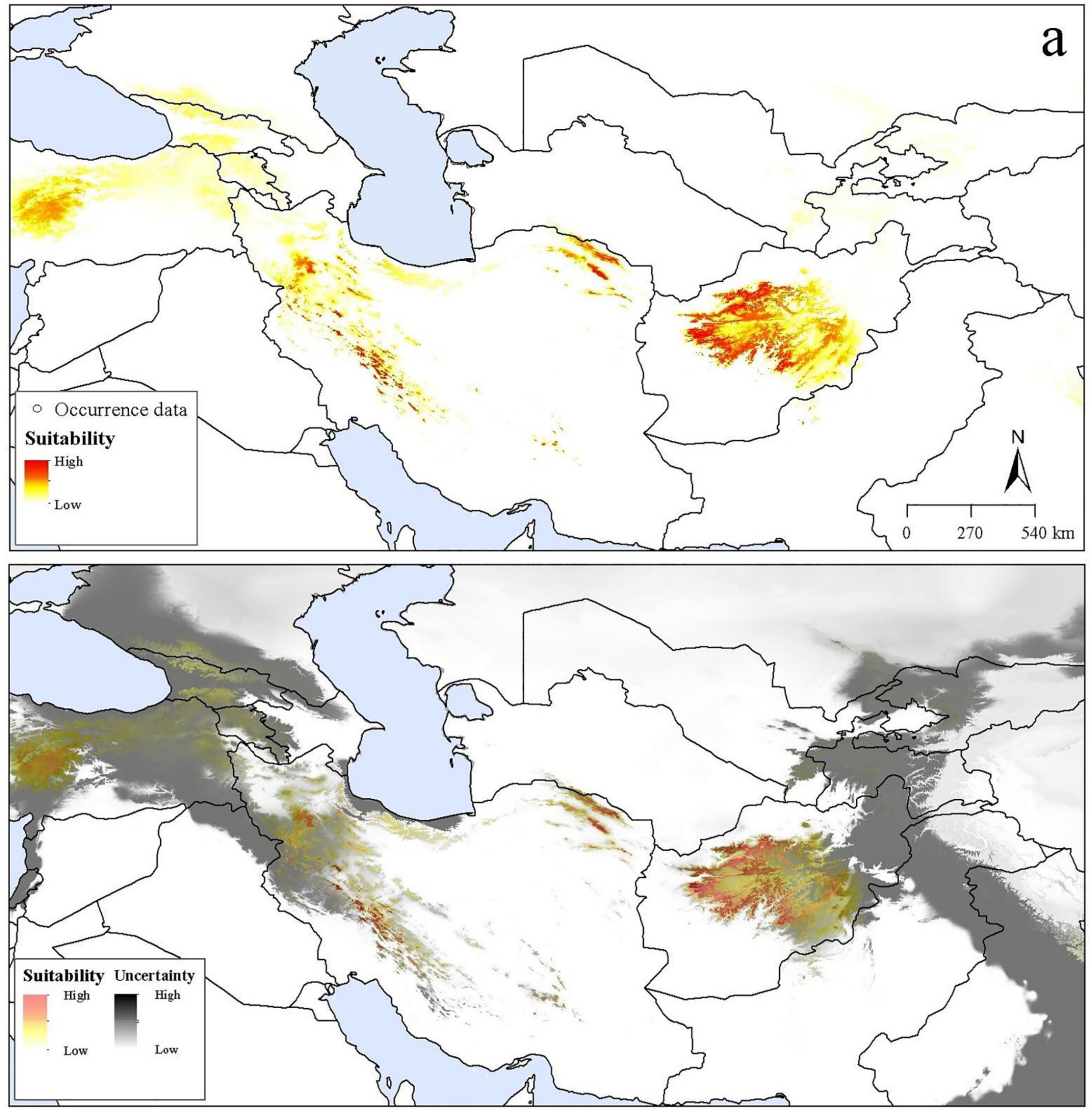
Potential future distribution of *Dianthus polylepis* under the Representative Concentration Pathway (RCP) 8.5 across the Iran-Turanian region. (a) Median prediction. (b) The uncertainty associated with the predicted suitable regions.

Transferring models to future conditions indicated an overall distributional pattern similar to that under present-day conditions; however, suitable areas shifted towards higher elevations under future conditions. The future potential distribution under RCP 4.5 was concentrated in montane areas in Iran and Afghanistan (Fig 3A). In contrast, suitable areas under RCP 8.5 were confined to the highest elevations in western and eastern Iran (Fig 4A). Uncertainty under future conditions was distributed similarly to that under present-day conditions (Figs 3B–4B).

**Fig 5:**
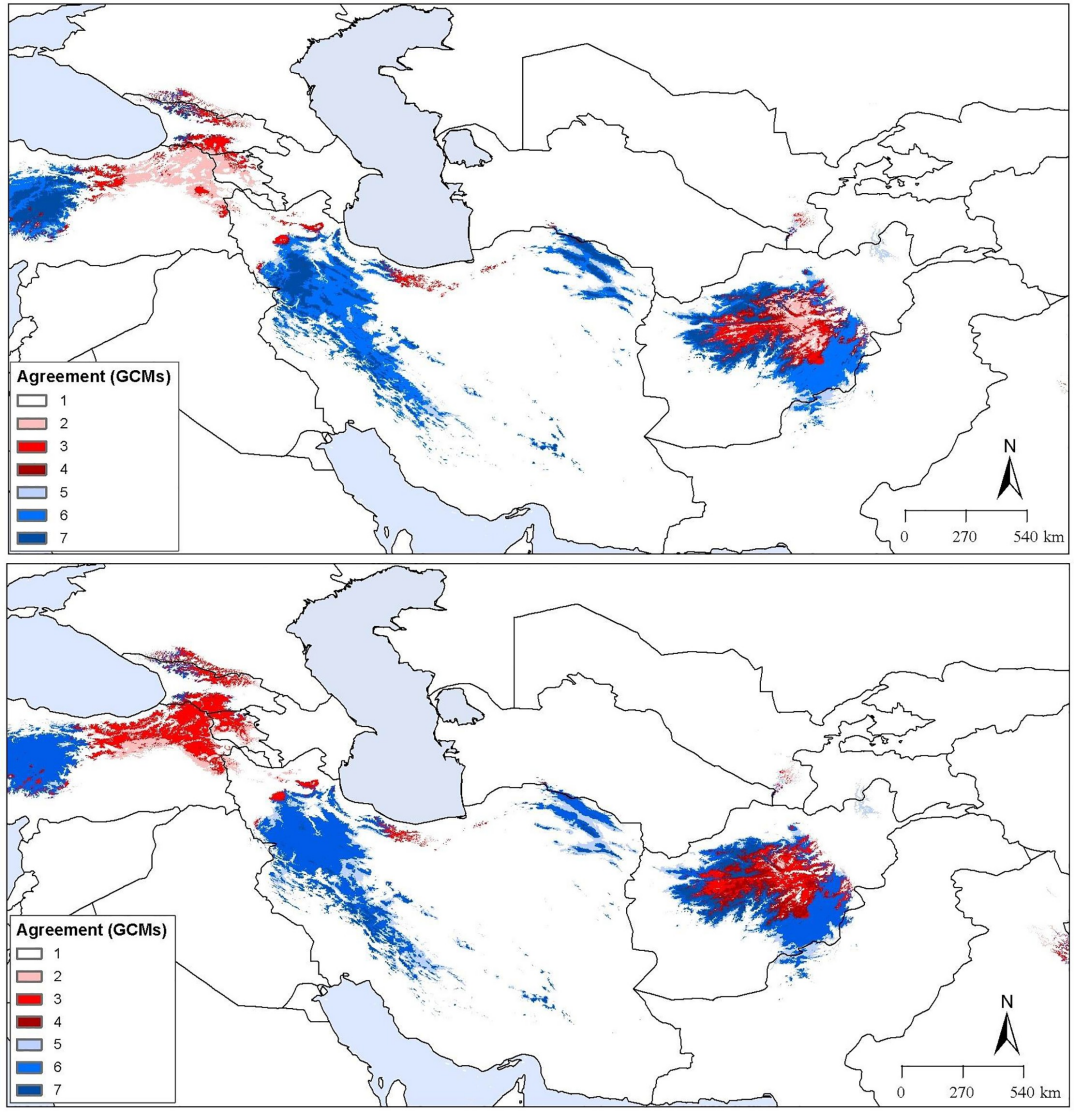
Predicted future suitable regions under current and future climate scenarios. (a) Prediction under the Representative Concentration Pathway (RCP) 4.5. (b) Prediction under the Representative Concentration Pathway (RCP) 8.5. Both panels illustrate the agreement between different global circulation models. White indicates areas not suitable under present or future conditions. Darkest blue indicates areas suitable under both present and future conditions. Redder shades indicate increasing possibility of future gain of suitability, whereas lighter blue shades indicate greater potential for less of suitability into the future.

Based on conditions anticipated for RCP 4.5 and RCP 8.5 (Fig 5A and 5B), an expansion of suitable areas was indicated in montane areas of sub-region IT2 in small parts of northeastern Turkey, northwestern Armenia, northern and western Georgia, northwestern and northern Iran, and central Afghanistan. Across the broader area of interest, the potential distribution of *D*. *polylepis* increased by 21.4% and 17.7% from present-day conditions to RCP 4.5 and RCP 8.5 conditions, respectively. However, across the model calibration area, which is more certainly accessible to the species, the distributional area of the species increased by only 2.9% from present-day conditions to RCP 4.5 conditions and 0.8% to RCR 8.5 conditions.

## Discussion

In this study, we estimated the potential geographic distribution of *D*. *polylepis* in the Irano-Turanian region under current and future climate conditions. *Dianthus polylepis* is restricted to montane areas, and the species’ distribution appears to have been molded by a combination of access by dispersal (i.e., it has not been able to colonize apparently suitable areas in montane areas that are relatively close by), past speciation history [67], and environmental conditions in terms of temperature and precipitation [30,68]. In particular, in our analyses, mean diurnal temperature range, iso-thermality, minimum temperature of coldest quarter, and annual precipitation, were factors of importance in delimiting the niche of this species. Although field-based information of this sort is not available for *D*. *polylepis*, a recent study by López-Jurado et al. [30] indicated that the distribution of another *Dianthus* species, the *D*. *broteri* complex, in the Mediterranean region is constrained by environmental variables related to temperature and drought stress (potential evapotranspiration in the driest and warmest quarters), along with edaphic properties. Distributions of *Dianthus* species in the *D*. *pungens* group depend on elevation and temperature [27].

The bioclimatic classification system [50,56] indicates a close relationship between vegetation distributions and climatic parameters in the Irano-Turanian region. Several such parameters differentiate the climate of this region from Mediterranean bioclimates, including lower annual precipitation, lower winter temperatures, slightly higher summer temperatures, distinctly higher continentality (diurnal temperature variation), and often a longer dry season (5 to 7 months) [50]. Annual precipitation and temperature annual range rank among the most important drivers of the distribution of *Artemisia aucheri* at a regional scale in the Alborz Mountains in northern Iran [69]. *Dianthus polylepis* is distributed in areas with two distinct Mediterranean bioclimates: long summer drought and low annual precipitation (MXC; Mediterranean xeric-continental) and high annual precipitation in winter months (MPC; Mediterranean pluviseasonal-continental). MXC covers broader areas in the Khorassan-Kopet Dagh floristic province than MPC, which is restricted to a small area between Kopet-Dagh and the Aladagh-Binalood Mountains [50]. Our results coincided with previous studies in showing the importance of climate parameters in montane habitats, especially temperature, as a crucial parameter in limiting local-to-regional-scale plant species’ distributions [27,70].

Our models indicated that almost all suitable habitats for *D*. *polylepis* are in montane areas in sub-region IT2, which has a distinctive climate. Accordingly, suitable areas identified in western Iran are climatically similar to the species’ distribution in the Khorassan-Kopet Dagh floristic province [50]. Our future range projections indicate potential for distributional expansion, yet an open question is whether the species will be able to access those all of those areas via dispersal, or whether some areas will prove uninhabitable owing to extreme conditions at higher elevations close to present distributional areas, such as possible effects of increased UV radiation [71].

Climate change could impact the availability of suitable areas for the species in the future. Both RCP scenarios showed considerable increases in suitable distributional areas mostly northward into the Euro-Siberian region [50]. However, on more local and more clearly accessible scales, changing and shifting suitability patterns were observed in current and future distribution areas, with upward shifts in elevation in areas adjacent to current distributional areas, which might ultimately lead to area reductions and local extinction (Figs 3 and 4). This decline of habitat suitability would be a consequence of a lack of adaptation of the species to changes in climate conditions, which appears to occur under limited circumstances with climate change [72]. In a recent experiment, solar radiation, wind speed, and minimum temperature were the climatic variables with the greatest effects on survival and stomatal conductance of the related species *D*. *inoxianus* [71]. Abiotic factors such as UV radiation and daily temperature fluctuations will be also exacerbated with elevation, which can adversely affect growth in montane areas [73–75]. Recent studies showed low genetic diversity in populations of some *Dianthus* species in montane areas, where they are exposed to harsh environments [37,76], which may make the species more vulnerable to climate change stresses. Furthermore, our field studies suggest that the growth of *D*. *polylepis* is constrained by these environmental stresses and that the species shows stress tolerance in the face of harsh environmental conditions. Land-use change and human activities have fragmented the species’ populations, creating small populations with low adaptive potential and increased extirpation rates in remnant habitats.

Whether the species will be able to access these new regions depend on several factors. Dispersal is important in shifting species’ ranges, particularly along elevational gradients [77]. Although many plant species are capable of long-distance dispersal, *Dianthus* species disperse their seeds only by semachory, and as such disperse only over relatively short distances [78,79]. Thus, *D*. *polylepis* will likely not be able to colonize new regions as they become suitable for the species. Furthermore, the Irano-Turanian region has a complex topography due to its complex tectonic history which provided a suitable physiographic context for the development of the region’s flora; such complex topography forms strong barriers that further constrain dispersal [80,81]. The main mountain ranges of the Irano-Anatolian plateaus and Central Asia have a prevailing W-E and NW-SE orientation, which complicates responses to warming climate conditions [56,82].

One should take into account some common complications that affect such correlative niche models. For example, occurrence data deficiency is a major challenge that must be considered to avoid over- or under-characterizing species’ niches [83,84]. As a consequence, we applied robust approaches using Maxent, and tuning model settings via jackknife and model selection approaches [23,85]. In addition, occurrence data are influenced by biases in geographic sampling, so we processed our models to thin the occurrence data spatially via removing records that were clumped or clustered artificially [51].

Another important factor that has received limited attention is whether or not the distribution of *D*. *polylepis* is in equilibrium with its environments and accessible landscape. Biotic interactions, dispersal characteristics, and human activities may affect this equilibrium and limit niche quantification and transferability. Therefore, the niches that are estimated using these methods may under-characterize the full dimensions of the fundamental niches that genuinely determine distributional potential [86]. However, we applied Maxent as a correlative modeling method in our study to predict, and interpret and explore the potential distribution of *D*. *polylepis* on a broad spatial scale; we were careful to interpret our results appropriately and not in excessive or unsupported detail.

## Conclusions

We showed that distributional patterns for *D*. *polylepis* can be modeled under current and future climate scenarios using relatively small numbers of occurrence records. Suitable areas for the species are concentrated in a few isolated mountain ranges; suitability may be reduced at local scales owing to climate change, which may be exacerbated by human activities and land use transformation in these areas. For this reason, immediate conservation actions are needed to protect this species from extinction. These results may serve as a basis for conservation actions concerning additional endemic plant species in the Irano-Turanian region. In addition, it may provide a view of the influences of various climatic parameters affecting the occurrence and distribution of species, which will be useful in developing strategies for monitoring species. However, further research is necessary to validate the present modeling results using direct field studies, to consider factors such as interspecific interactions, the impact of land-use change, and the role of topographic factors and geographic barriers in constraining the species’ distributional potential.

## Supporting information

S1 Table Summary of occurrence data used in this study. (DOCX)

S2 Table Summary of model parameter settings explored and tested in this study. (DOCX)
