# Supplementary material for: Climate change influences on the potential distribution of Dianthus polylepis Bien. ex Boiss. (Caryophyllaceae), an endemic species in the Irano-Turanian region
Source: PLoS One. 2020 Aug 18;15(8):e0237527. doi: 10.1371/journal.pone.0237527 (PMC7437464; doi:10.1371/journal.pone.0237527)
Supplement: S1 Table — (DOCX) [file pone.0237527.s001.docx]

S1 Table. Summary of occurrence data used in this study

| *Longtitude* | *Latitude* | *Location* | *Sources* | *No. Herbarium* | *Date* |
| --- | --- | --- | --- | --- | --- |
| 59.8435 | 35.6243 | Fariman Dam | Field Survey |  | 5/6/2018 |
| 59.6 | 36.169 | S Mashhad, Deh Gheibi | Field Survey |  | 8/23/2018 |
| 59.5667 | 36.7219 | N Mashhad, Kardeh Dam | Field Survey |  | 5/6/2018 |
| 59.186 | 36.324 | W Mashhad, between Zoshk and Kang | Field Survey |  | 5/27/2018 |
| 58.963 | 36.671 | S Chenaran, Akhlamad | Field Survey |  | 6/9/2018 |
| 59.383 | 36.133 | S Mashhad, Moghan | Field Survey |  | 5/29/2018 |
| 58.4842 | 35.4088 | N Kashmar, S Rivash, km 9 | Field Survey |  | 5/30/2016 |
| 58.5519 | 36.768 | NW Neyshabour, between Hasan Abad and Chahab | Field Survey |  | 6/2/2016 |
| 59.3002 | 36.0836 | SW Mashhad, between GhasemAbad and Dizbad-e bala | Field Survey |  | 6/3/2016 |
| 59.3006 | 36.0834 | SW Mashhad, between GhasemAbad and Dizbad-e bala | Field Survey |  | 6/3/2016 |
| 59.3668 | 36.184 | S Mashhad, Moghan Mountains | Field Survey |  | 5/27/2016 |
| 59.3679 | 36.1533 | S Mashhad, Moghan Mountains | Field Survey |  | 5/27/2016 |
| 59.636 | 36.6549 | N Mashhad, Kardeh | Field Survey |  | 5/25/2016 |
| 59.8875 | 36.6432 | N Mashhad, Khowr Mountains | Field Survey |  | 5/24/2016 |
| 58.4702 | 37.4604 | N Ghouchan, Dorbadam | Field Survey |  | 5/30/2017 |
| 58.4953 | 35.4208 | N Kashmar, S Rivash, km 6 | Field Survey |  | 5/30/2017 |
| 58.6677 | 36.6921 | NW Neyshabour, Baharkish | Field Survey |  | 6/3/2017 |
| 58.9561 | 36.4794 | S Chenaran, Fereizi, Dahane Jaji | Field Survey |  | 5/25/2017 |
| 59.2106 | 36.3314 | W Mashhad, Zoshk | Field Survey |  | 5/9/2017 |
| 58.478 | 37.227 | N Ghouchan, Alamli Pass. | Flora Iranica | 1664 |  |
| 58.488 | 37.276 | N Ghouchan | Flora Iranica | 1641 |  |
| 59.365 | 36.815 | NE Chenaran, between Ardak and Talghour | Flora Iranica | 4901 |  |
| 59.393 | 35.695 | S Mashhad, south mountains of Robat Sefid | Flora Iranica | 1580 |  |
| 59.853 | 35.642 | Fariman Dam | Flora Iranica | 10934 |  |
| 60.363 | 35.185 | SW Torbat-e Jam, Bezd Mountain | Flora Iranica | 715 |  |
| 57.398 | 38.05 | Turkmenistan, SE Solukli | Flora Iranica | 870 |  |
| 57.461 | 37.987 | Turkmenistan, Sulukli Mountain | Flora Iranica | 709 |  |
| 57.621 | 38.027 | Turkmenistan, SW Kulkulab | Flora Iranica | 1082 |  |
| 59.352 | 36.249 | SW Mashhad, Kalate Zabetian | FUMH | 10473 | 6/16/1983 |
| 59.843 | 36.629 | N Mashhad, on Kalat Road, 50 km towards Kalat | FUMH | 11005 | 5/13/1984 |
| 59.897 | 35.5 | Fariman, Chahar Tekab | FUMH | 11298 | 5/28/1984 |
| 59.053 | 36.344 | S Chenaran, Cheshmeh Sabz | FUMH | 13572 | 8/13/1985 |
| 60.071 | 35.411 | NW Torbate- Jam, Bardo Forest | FUMH | 15406 | 6/28/1989 |
| 59.348 | 36.118 | S Mashhad, Moghan, mountains of Moghan cave | FUMH | 18710 | 6/2/1990 |
| 58.496 | 35.442 | N Kashmar, 25 km towards Neyshabour | FUMH | 20733 | 6/12/1991 |
| 58.724 | 36.939 | SE Ghouchan, between Gholmakharan and Borselan | FUMH | 44419 | 3/17/1991 |
| 59.34 | 36.064 | SW Mashhad, north mountains of Pivejan | FUMH | 23377 | 6/16/1993 |
| 58.933 | 36.45 | S Chenaran, Dahane Jaji | FUMH | 24075 | 6/1/1994 |
| 58.906 | 36.507 | S Chenaran, Fereizi, Ghorghi | FUMH | 24116 | 6/1/1994 |
| 59.203 | 36.296 | W Mashhad, Kang Mountains | FUMH | 27624 | 6/25/1996 |
| 59.179 | 35.518 | Torbat-e Heydariyeh towards Mashhad, km 30 | FUMH | 27677 | 7/1/1996 |
| 59.613 | 36.837 | N Mashhad, Balghour | FUMH | 27535 | 6/23/1996 |
| 58.249 | 37.435 | N Faruj, Ghale Safa Mountains | FUMH | 29326 | 6/23/1997 |
| 58.942 | 35.301 | NE Kashmar, Ghale Joogh Mountains | FUMH | 28888 | 5/28/1997 |
| 59.341 | 36.107 | S Mashhad, Moghan | FUMH | 33675 | 6/3/2001 |
| 60.049 | 35.424 | NW Torbat-e Jam, Bardo Forest | FUMH | 34805 | 5/4/2002 |
| 58.751 | 36.515 | NW Neyshabour, Bar waterfall. | FUMH | 34885 | 6/29/2003 |
| 58.919 | 36.592 | S Chenaran, Akhlamad Fall | FUMH | 36553 | 6/8/2005 |
| 60.353 | 35.194 | SW Torbat-e Jam, Bezd Mountain | FUMH | 36866 | 7/12/2005 |
| 58.934 | 36.483 | S Chenaran, Fereizi towards Mahale Sorkhe, km 2 | FUMH | 36481 | 5/29/2005 |
| 59.255 | 36.267 | W Mashhad, Kordineh | FUMH | 36532 | 6/7/2005 |
| 59.189 | 35.501 | N Torbat-e Heydariyeh, Khomari | FUMH | 39170 | 7/15/2007 |
| 59.329 | 36.109 | SW Mashhad, North mountains of Pivejan | FUMH | 40344 | 5/24/2008 |
| 59.39 | 36.121 | S Mashhad, between Moghan and Moghan Cave | FUMH | 43037 | 6/14/2009 |
| 59.317 | 36.1 | SW Mashhad, North mountains of Pivejan | FUMH | 43070 | 6/16/2009 |
| 59.5 | 36.848 | NW Mashhad, KarimAbad | FUMH | 44210 | 8/5/2010 |
| 59.691 | 36.148 | SE Mashhad, Khajeh Morad | FUMH | 44218 | 8/29/2010 |
| 58.734 | 37.469 | N Ghouchan, Dorbadam Valley | FUMH | 44428 | 5/4/2011 |
| 59.176 | 36.316 | W Mashhad, Cheshme Ghoghli | FUMH | 44456 | 6/19/2011 |
| 57.9575 | 37.8104 | N Shirvan, Sarani Mountains | FUMH | 44951 | 7/9/2012 |
| 58.7278 | 36.5147 | NW Neyshabour, Bar Waterfall. | FUMH | 45109 | 6/28/2013 |
| 58.4602 | 35.525 | Kashmar, N Rivash, Aria Mountain | FUMH | 45753 | 8/5/2016 |
| 58.965 | 36.234 | Neyshabour, Bujan | TARI | 48955 |  |
| 59.217 | 35.95 | SW Mashhad, Dizbad | TARI | 48932 |  |
| 58.461 | 35.299 | Kashmar towards Neyshabour, km 14 | TARI , Assadi | 35585 |  |
| 58.468 | 35.331 | Kashmar towards Neyshabour, km 20 | TARI , Assadi | 35536 |  |
| 58.63 | 35.628 | Kashmar towards Neyshabour, km 50 | TARI , Assadi | 35738 |  |
| 59.168 | 36.337 | W Mashhad, N. Zoshk village | TARI , Assadi | 48826 |  |
| 59.399 | 35.796 | S Mashhad, Robat Sefid | TARI , Assadi | 21305 |  |
| 59.814 | 36.579 | Mashhad towards Kalat, km 45 | TARI , Assadi | 21372 |  |
