# Supplementary material for: Climate change influences on the potential distribution of Dianthus polylepis Bien. ex Boiss. (Caryophyllaceae), an endemic species in the Irano-Turanian region
Source: PLoS One. 2020 Aug 18;15(8):e0237527. doi: 10.1371/journal.pone.0237527 (PMC7437464; doi:10.1371/journal.pone.0237527)
Supplement: S2 Table — (DOCX) [file pone.0237527.s002.docx]

S2 Table. Summary of model parameter settings explored and tested in this study

| *Regularization multiplier* | *Feature classes* | *Environmental sets* | *Partial_ROC* | *Omission rate (E=7%)* | *AICc* | delta_AICc | W_AICc | *Number of parameters* |
| --- | --- | --- | --- | --- | --- | --- | --- | --- |
| 0.1 | l | set_1 | 0.00 | 0.16 | 1173.4 | 61.5 | 2.6 | 5 |
| 0.1 | l | set_2 | 0.00 | 0.24 | 1178.8 | 66.9 | 1.8 | 4 |
| 0.1 | l | set_3 | 0.00 | 0.2 | 1181.6 | 69.7 | 4.4 | 3 |
| 0.1 | q | set_1 | 0.00 | 0.32 | 1169.5 | 57.6 | 1.8 | 5 |
| 0.1 | q | set_2 | 0.00 | 0.2 | 1169.5 | 57.6 | 1.9 | 4 |
| 0.1 | q | set_3 | 0.00 | 0.28 | 1186.1 | 74.2 | 4.7 | 3 |
| 0.1 | p | set_1 | 0.00 | 0.24 | 1153.7 | 41.7 | 5.3 | 9 |
| 0.1 | p | set_2 | 0.00 | 0.24 | 1156.3 | 44.4 | 1.4 | 6 |
| 0.1 | p | set_3 | 0.00 | 0.12 | 1176.3 | 64.4 | 6.3 | 3 |
| 0.1 | t | set_1 | 0.25 | 0.68 | NA | NA | NA | 82 |
| 0.1 | t | set_2 | 0.27 | 0.64 | NA | NA | NA | 69 |
| 0.1 | t | set_3 | 0.25 | 0.56 | NA | NA | NA | 66 |
| 0.1 | h | set_1 | 0.00 | 0.32 | 3408.7 | 2296.8 | 0.0 | 47 |
| 0.1 | h | set_2 | 0.00 | 0.24 | 1576.5 | 464.5 | 8.2 | 41 |
| 0.1 | h | set_3 | 0.00 | 0.24 | 2208.9 | 1097.0 | 3.8 | 45 |
| 0.1 | lq | set_1 | 0.00 | 0.2 | 1134.7 | 22.8 | 6.9 | 10 |
| 0.1 | lq | set_2 | 0.00 | 0.16 | 1126.8 | 14.9 | 3.5 | 8 |
| 0.1 | lq | set_3 | 0.00 | 0.16 | 1125.3 | 13.4 | 7.6 | 6 |
| 0.1 | lp | set_1 | 0.00 | 0.2 | 1154.6 | 42.7 | 3.2 | 12 |
| 0.1 | lp | set_2 | 0.00 | 0.24 | 1161.8 | 49.9 | 9.1 | 8 |
| 0.1 | lp | set_3 | 0.00 | 0.12 | 1165.3 | 53.4 | 1.5 | 5 |
| 0.1 | lt | set_1 | 0.23 | 0.68 | NA | NA | NA | 82 |
| 0.1 | lt | set_2 | 0.24 | 0.64 | NA | NA | NA | 69 |
| 0.1 | lt | set_3 | 0.23 | 0.56 | NA | NA | NA | 66 |
| 0.1 | lh | set_1 | 0.00 | 0.32 | NA | NA | NA | 49 |
| 0.1 | lh | set_2 | 0.00 | 0.24 | 1576.5 | 464.5 | 8.2 | 41 |
| 0.1 | lh | set_3 | 0.00 | 0.24 | 2208.9 | 1097.0 | 3.8 | 45 |
| 0.1 | qp | set_1 | 0.00 | 0.16 | 1126.9 | 15.0 | 3.4 | 10 |
| 0.1 | qp | set_2 | 0.00 | 0.12 | 1123.0 | 11.1 | 2.4 | 7 |
| 0.1 | qp | set_3 | 0.00 | 0.12 | 1120.0 | 8.1 | 1.0 | 6 |
| 0.1 | qt | set_1 | 0.25 | 0.68 | NA | NA | NA | 83 |
| 0.1 | qt | set_2 | 0.00 | 0.64 | NA | NA | NA | 69 |
| 0.1 | qt | set_3 | 0.41 | 0.56 | NA | NA | NA | 69 |
| 0.1 | qh | set_1 | 0.00 | 0.36 | NA | NA | NA | 49 |
| 0.1 | qh | set_2 | 0.00 | 0.24 | 1664.5 | 552.5 | 6.4 | 42 |
| 0.1 | qh | set_3 | 0.00 | 0.24 | 1473.5 | 361.6 | 1.9 | 39 |
| 0.1 | pt | set_1 | 0.22 | 0.68 | NA | NA | NA | 82 |
| 0.1 | pt | set_2 | 0.27 | 0.64 | NA | NA | NA | 69 |
| 0.1 | pt | set_3 | 0.24 | 0.56 | NA | NA | NA | 66 |
| 0.1 | ph | set_1 | 0.00 | 0.32 | NA | NA | NA | 52 |
| 0.1 | ph | set_2 | 0.00 | 0.24 | 2180.3 | 1068.4 | 6.1 | 45 |
| 0.1 | ph | set_3 | 0.00 | 0.24 | 1589.4 | 477.5 | 1.2 | 41 |
| 0.1 | th | set_1 | 0.23 | 0.72 | NA | NA | NA | 87 |
| 0.1 | th | set_2 | 0.00 | 0.6 | NA | NA | NA | 76 |
| 0.1 | th | set_3 | 0.23 | 0.6 | NA | NA | NA | 73 |
| 0.1 | lqp | set_1 | 0.00 | 0.16 | 1130.9 | 19.0 | 4.6 | 11 |
| 0.1 | lqp | set_2 | 0.00 | 0.12 | 1128.5 | 16.5 | 1.5 | 9 |
| 0.1 | lqp | set_3 | 0.00 | 0.12 | 1120.0 | 8.1 | 1.0 | 6 |
| 0.1 | lqt | set_1 | 0.25 | 0.68 | NA | NA | NA | 83 |
| 0.1 | lqt | set_2 | 0.00 | 0.64 | NA | NA | NA | 69 |
| 0.1 | lqt | set_3 | 0.39 | 0.56 | NA | NA | NA | 69 |
| 0.1 | lqh | set_1 | 0.00 | 0.36 | NA | NA | NA | 51 |
| 0.1 | lqh | set_2 | 0.00 | 0.24 | 1944.6 | 832.6 | 9.7 | 44 |
| 0.1 | lqh | set_3 | 0.00 | 0.24 | 1473.5 | 361.6 | 1.9 | 39 |
| 0.1 | lpt | set_1 | 0.28 | 0.68 | NA | NA | NA | 82 |
| 0.1 | lpt | set_2 | 0.25 | 0.64 | NA | NA | NA | 69 |
| 0.1 | lpt | set_3 | 0.27 | 0.56 | NA | NA | NA | 66 |
| 0.1 | lph | set_1 | 0.00 | 0.32 | NA | NA | NA | 53 |
| 0.1 | lph | set_2 | 0.00 | 0.24 | 2180.8 | 1068.9 | 4.8 | 45 |
| 0.1 | lph | set_3 | 0.00 | 0.24 | 1589.4 | 477.5 | 1.2 | 41 |
| 0.1 | qpt | set_1 | 0.22 | 0.68 | NA | NA | NA | 82 |
| 0.1 | qpt | set_2 | 0.00 | 0.64 | NA | NA | NA | 70 |
| 0.1 | qpt | set_3 | 0.39 | 0.56 | NA | NA | NA | 68 |
| 0.1 | qph | set_1 | 0.00 | 0.32 | NA | NA | NA | 56 |
| 0.1 | qph | set_2 | 0.00 | 0.24 | 2589.1 | 1477.1 | 0.0 | 46 |
| 0.1 | qph | set_3 | 0.00 | 0.28 | 1466.5 | 354.6 | 6.2 | 39 |
| 0.1 | qth | set_1 | 0.24 | 0.72 | NA | NA | NA | 87 |
| 0.1 | qth | set_2 | 0.00 | 0.6 | NA | NA | NA | 76 |
| 0.1 | qth | set_3 | 0.27 | 0.6 | NA | NA | NA | 73 |
| 0.1 | pth | set_1 | 0.26 | 0.72 | NA | NA | NA | 87 |
| 0.1 | pth | set_2 | 0.00 | 0.6 | NA | NA | NA | 76 |
| 0.1 | pth | set_3 | 0.23 | 0.6 | NA | NA | NA | 73 |
| 0.1 | lqpt | set_1 | 0.22 | 0.68 | NA | NA | NA | 82 |
| 0.1 | lqpt | set_2 | 0.00 | 0.64 | NA | NA | NA | 70 |
| 0.1 | lqpt | set_3 | 0.39 | 0.56 | NA | NA | NA | 68 |
| 0.1 | lqph | set_1 | 0.00 | 0.32 | NA | NA | NA | 53 |
| 0.1 | lqph | set_2 | 0.00 | 0.24 | NA | NA | NA | 51 |
| 0.1 | lqph | set_3 | 0.00 | 0.28 | 1589.8 | 477.9 | 1.0 | 41 |
| 0.1 | lqth | set_1 | 0.30 | 0.72 | NA | NA | NA | 87 |
| 0.1 | lqth | set_2 | 0.00 | 0.6 | NA | NA | NA | 76 |
| 0.1 | lqth | set_3 | 0.22 | 0.6 | NA | NA | NA | 73 |
| 0.1 | lpth | set_1 | 0.24 | 0.72 | NA | NA | NA | 87 |
| 0.1 | lpth | set_2 | 0.00 | 0.6 | NA | NA | NA | 76 |
| 0.1 | lpth | set_3 | 0.20 | 0.6 | NA | NA | NA | 73 |
| 0.1 | lqpth | set_1 | 0.24 | 0.72 | NA | NA | NA | 88 |
| 0.1 | lqpth | set_2 | 0.00 | 0.6 | NA | NA | NA | 76 |
| 0.1 | lqpth | set_3 | 0.25 | 0.6 | NA | NA | NA | 73 |
| 0.2 | l | set_1 | 0.00 | 0.16 | 1173.5 | 61.6 | 2.6 | 5 |
| 0.2 | l | set_2 | 0.00 | 0.24 | 1178.8 | 66.9 | 1.8 | 4 |
| 0.2 | l | set_3 | 0.00 | 0.2 | 1181.6 | 69.7 | 4.5 | 3 |
| 0.2 | q | set_1 | 0.00 | 0.32 | 1169.6 | 57.6 | 1.9 | 5 |
| 0.2 | q | set_2 | 0.00 | 0.2 | 1169.5 | 57.6 | 1.9 | 4 |
| 0.2 | q | set_3 | 0.00 | 0.28 | 1186.1 | 74.2 | 4.8 | 3 |
| 0.2 | p | set_1 | 0.00 | 0.24 | 1150.0 | 38.1 | 3.3 | 7 |
| 0.2 | p | set_2 | 0.00 | 0.24 | 1159.3 | 47.4 | 3.2 | 5 |
| 0.2 | p | set_3 | 0.00 | 0.12 | 1176.6 | 64.7 | 5.5 | 3 |
| 0.2 | t | set_1 | 0.00 | 0.68 | NA | NA | NA | 65 |
| 0.2 | t | set_2 | 0.00 | 0.6 | NA | NA | NA | 53 |
| 0.2 | t | set_3 | 0.00 | 0.56 | NA | NA | NA | 50 |
| 0.2 | h | set_1 | 0.00 | 0.28 | 1240.0 | 128.1 | 9.7 | 31 |
| 0.2 | h | set_2 | 0.00 | 0.24 | 1195.1 | 83.2 | 5.4 | 27 |
| 0.2 | h | set_3 | 0.00 | 0.28 | 1173.0 | 61.0 | 3.5 | 22 |
| 0.2 | lq | set_1 | 0.00 | 0.16 | 1133.0 | 21.1 | 1.6 | 8 |
| 0.2 | lq | set_2 | 0.00 | 0.12 | 1132.0 | 20.1 | 2.7 | 8 |
| 0.2 | lq | set_3 | 0.00 | 0.12 | 1129.6 | 17.7 | 8.9 | 6 |
| 0.2 | lp | set_1 | 0.00 | 0.24 | 1148.3 | 36.3 | 8.1 | 7 |
| 0.2 | lp | set_2 | 0.00 | 0.24 | 1158.7 | 46.8 | 4.3 | 5 |
| 0.2 | lp | set_3 | 0.00 | 0.16 | 1163.4 | 51.4 | 4.3 | 4 |
| 0.2 | lt | set_1 | 0.00 | 0.64 | NA | NA | NA | 66 |
| 0.2 | lt | set_2 | 0.00 | 0.6 | NA | NA | NA | 56 |
| 0.2 | lt | set_3 | 0.00 | 0.56 | NA | NA | NA | 52 |
| 0.2 | lh | set_1 | 0.00 | 0.28 | 1256.0 | 144.0 | 3.3 | 32 |
| 0.2 | lh | set_2 | 0.00 | 0.24 | 1195.1 | 83.2 | 5.4 | 27 |
| 0.2 | lh | set_3 | 0.00 | 0.28 | 1173.2 | 61.3 | 3.0 | 22 |
| 0.2 | qp | set_1 | 0.00 | 0.16 | 1128.3 | 16.4 | 1.7 | 8 |
| 0.2 | qp | set_2 | 0.00 | 0.12 | 1126.1 | 14.2 | 5.2 | 6 |
| 0.2 | qp | set_3 | 0.00 | 0.12 | 1123.6 | 11.7 | 1.8 | 5 |
| 0.2 | qt | set_1 | 0.00 | 0.6 | NA | NA | NA | 67 |
| 0.2 | qt | set_2 | 0.00 | 0.6 | NA | NA | NA | 57 |
| 0.2 | qt | set_3 | 0.00 | 0.56 | NA | NA | NA | 53 |
| 0.2 | qh | set_1 | 0.00 | 0.28 | 1273.6 | 161.6 | 5.0 | 33 |
| 0.2 | qh | set_2 | 0.00 | 0.24 | 1195.5 | 83.6 | 4.4 | 27 |
| 0.2 | qh | set_3 | 0.00 | 0.28 | 1195.9 | 83.9 | 3.7 | 25 |
| 0.2 | pt | set_1 | 0.00 | 0.68 | NA | NA | NA | 64 |
| 0.2 | pt | set_2 | 0.00 | 0.6 | NA | NA | NA | 56 |
| 0.2 | pt | set_3 | 0.00 | 0.56 | NA | NA | NA | 52 |
| 0.2 | ph | set_1 | 0.00 | 0.28 | 1372.2 | 260.2 | 1.9 | 37 |
| 0.2 | ph | set_2 | 0.00 | 0.24 | 1215.8 | 103.8 | 1.8 | 29 |
| 0.2 | ph | set_3 | 0.00 | 0.28 | 1169.5 | 57.6 | 1.9 | 22 |
| 0.2 | th | set_1 | 0.00 | 0.64 | NA | NA | NA | 69 |
| 0.2 | th | set_2 | 0.00 | 0.64 | NA | NA | NA | 60 |
| 0.2 | th | set_3 | 0.00 | 0.56 | NA | NA | NA | 58 |
| 0.2 | lqp | set_1 | 0.00 | 0.16 | 1128.6 | 16.7 | 1.5 | 8 |
| 0.2 | lqp | set_2 | 0.00 | 0.12 | 1126.2 | 14.3 | 4.9 | 6 |
| 0.2 | lqp | set_3 | 0.00 | 0.12 | 1123.7 | 11.8 | 1.7 | 5 |
| 0.2 | lqt | set_1 | 0.00 | 0.6 | NA | NA | NA | 67 |
| 0.2 | lqt | set_2 | 0.00 | 0.6 | NA | NA | NA | 56 |
| 0.2 | lqt | set_3 | 0.00 | 0.56 | NA | NA | NA | 54 |
| 0.2 | lqh | set_1 | 0.00 | 0.28 | 1376.2 | 264.3 | 2.5 | 37 |
| 0.2 | lqh | set_2 | 0.00 | 0.24 | 1185.7 | 73.8 | 6.1 | 26 |
| 0.2 | lqh | set_3 | 0.00 | 0.28 | 1204.7 | 92.8 | 4.5 | 26 |
| 0.2 | lpt | set_1 | 0.00 | 0.68 | NA | NA | NA | 64 |
| 0.2 | lpt | set_2 | 0.00 | 0.6 | NA | NA | NA | 56 |
| 0.2 | lpt | set_3 | 0.00 | 0.56 | NA | NA | NA | 52 |
| 0.2 | lph | set_1 | 0.00 | 0.28 | 1370.9 | 259.0 | 3.6 | 37 |
| 0.2 | lph | set_2 | 0.00 | 0.24 | 1203.2 | 91.3 | 9.6 | 28 |
| 0.2 | lph | set_3 | 0.00 | 0.28 | 1169.5 | 57.6 | 2.0 | 22 |
| 0.2 | qpt | set_1 | 0.00 | 0.6 | NA | NA | NA | 67 |
| 0.2 | qpt | set_2 | 0.00 | 0.6 | NA | NA | NA | 57 |
| 0.2 | qpt | set_3 | 0.00 | 0.56 | NA | NA | NA | 53 |
| 0.2 | qph | set_1 | 0.00 | 0.28 | 1409.8 | 297.9 | 1.3 | 38 |
| 0.2 | qph | set_2 | 0.00 | 0.24 | 1175.0 | 63.1 | 1.2 | 25 |
| 0.2 | qph | set_3 | 0.00 | 0.28 | 1177.1 | 65.2 | 4.5 | 23 |
| 0.2 | qth | set_1 | 0.00 | 0.6 | NA | NA | NA | 70 |
| 0.2 | qth | set_2 | 0.00 | 0.6 | NA | NA | NA | 60 |
| 0.2 | qth | set_3 | 0.00 | 0.56 | NA | NA | NA | 58 |
| 0.2 | pth | set_1 | 0.00 | 0.6 | NA | NA | NA | 68 |
| 0.2 | pth | set_2 | 0.00 | 0.64 | NA | NA | NA | 59 |
| 0.2 | pth | set_3 | 0.00 | 0.56 | NA | NA | NA | 62 |
| 0.2 | lqpt | set_1 | 0.00 | 0.6 | NA | NA | NA | 67 |
| 0.2 | lqpt | set_2 | 0.00 | 0.6 | NA | NA | NA | 57 |
| 0.2 | lqpt | set_3 | 0.00 | 0.56 | NA | NA | NA | 53 |
| 0.2 | lqph | set_1 | 0.00 | 0.28 | 1410.1 | 298.2 | 1.1 | 38 |
| 0.2 | lqph | set_2 | 0.00 | 0.24 | 1204.4 | 92.5 | 5.3 | 28 |
| 0.2 | lqph | set_3 | 0.00 | 0.28 | 1184.5 | 72.6 | 1.1 | 24 |
| 0.2 | lqth | set_1 | 0.00 | 0.6 | NA | NA | NA | 70 |
| 0.2 | lqth | set_2 | 0.00 | 0.6 | NA | NA | NA | 60 |
| 0.2 | lqth | set_3 | 0.00 | 0.56 | NA | NA | NA | 58 |
| 0.2 | lpth | set_1 | 0.00 | 0.6 | NA | NA | NA | 68 |
| 0.2 | lpth | set_2 | 0.00 | 0.64 | NA | NA | NA | 59 |
| 0.2 | lpth | set_3 | 0.00 | 0.56 | NA | NA | NA | 62 |
| 0.2 | lqpth | set_1 | 0.00 | 0.6 | NA | NA | NA | 70 |
| 0.2 | lqpth | set_2 | 0.00 | 0.64 | NA | NA | NA | 59 |
| 0.2 | lqpth | set_3 | 0.00 | 0.56 | NA | NA | NA | 62 |
| 0.3 | l | set_1 | 0.00 | 0.16 | 1173.6 | 61.6 | 2.6 | 5 |
| 0.3 | l | set_2 | 0.00 | 0.24 | 1178.9 | 66.9 | 1.8 | 4 |
| 0.3 | l | set_3 | 0.00 | 0.2 | 1181.6 | 69.7 | 4.7 | 3 |
| 0.3 | q | set_1 | 0.00 | 0.32 | 1169.6 | 57.7 | 1.9 | 5 |
| 0.3 | q | set_2 | 0.00 | 0.2 | 1169.5 | 57.6 | 1.9 | 4 |
| 0.3 | q | set_3 | 0.00 | 0.28 | 1186.1 | 74.2 | 4.9 | 3 |
| 0.3 | p | set_1 | 0.00 | 0.24 | 1151.9 | 39.9 | 1.3 | 7 |
| 0.3 | p | set_2 | 0.00 | 0.24 | 1160.1 | 48.2 | 2.2 | 5 |
| 0.3 | p | set_3 | 0.00 | 0.12 | 1177.1 | 65.2 | 4.4 | 3 |
| 0.3 | t | set_1 | 0.00 | 0.48 | 2559.8 | 1447.9 | 2.5 | 46 |
| 0.3 | t | set_2 | 0.00 | 0.56 | 1283.9 | 172.0 | 2.9 | 35 |
| 0.3 | t | set_3 | 0.00 | 0.48 | 1300.4 | 188.5 | 7.6 | 35 |
| 0.3 | h | set_1 | 0.00 | 0.28 | 1162.4 | 50.5 | 6.9 | 23 |
| 0.3 | h | set_2 | 0.00 | 0.24 | 1140.5 | 28.5 | 4.1 | 19 |
| 0.3 | h | set_3 | 0.00 | 0.32 | 1140.9 | 29.0 | 3.3 | 15 |
| 0.3 | lq | set_1 | 0.00 | 0.16 | 1131.5 | 19.6 | 3.6 | 7 |
| 0.3 | lq | set_2 | 0.00 | 0.12 | 1130.5 | 18.6 | 5.9 | 6 |
| 0.3 | lq | set_3 | 0.00 | 0.12 | 1128.1 | 16.2 | 1.9 | 5 |
| 0.3 | lp | set_1 | 0.00 | 0.24 | 1146.4 | 34.4 | 2.1 | 6 |
| 0.3 | lp | set_2 | 0.00 | 0.24 | 1159.5 | 47.6 | 2.9 | 5 |
| 0.3 | lp | set_3 | 0.00 | 0.16 | 1164.1 | 52.1 | 3.1 | 4 |
| 0.3 | lt | set_1 | 0.00 | 0.52 | NA | NA | NA | 52 |
| 0.3 | lt | set_2 | 0.00 | 0.56 | 1477.9 | 365.9 | 2.2 | 40 |
| 0.3 | lt | set_3 | 0.00 | 0.48 | 1327.3 | 215.3 | 1.1 | 36 |
| 0.3 | lh | set_1 | 0.00 | 0.28 | 1162.2 | 50.2 | 8.0 | 23 |
| 0.3 | lh | set_2 | 0.00 | 0.24 | 1135.5 | 23.6 | 4.8 | 18 |
| 0.3 | lh | set_3 | 0.00 | 0.32 | 1150.1 | 38.2 | 3.3 | 17 |
| 0.3 | qp | set_1 | 0.00 | 0.16 | 1130.6 | 18.7 | 5.7 | 8 |
| 0.3 | qp | set_2 | 0.00 | 0.12 | 1127.9 | 15.9 | 2.2 | 6 |
| 0.3 | qp | set_3 | 0.00 | 0.12 | 1125.3 | 13.3 | 8.2 | 5 |
| 0.3 | qt | set_1 | 0.00 | 0.6 | NA | NA | NA | 49 |
| 0.3 | qt | set_2 | 0.00 | 0.48 | 1545.8 | 433.9 | 3.9 | 41 |
| 0.3 | qt | set_3 | 0.00 | 0.52 | 1327.4 | 215.5 | 1.0 | 36 |
| 0.3 | qh | set_1 | 0.00 | 0.28 | 1148.9 | 36.9 | 6.2 | 21 |
| 0.3 | qh | set_2 | 0.00 | 0.24 | 1141.0 | 29.1 | 3.1 | 19 |
| 0.3 | qh | set_3 | 0.00 | 0.32 | 1133.1 | 21.2 | 1.6 | 13 |
| 0.3 | pt | set_1 | 0.00 | 0.52 | 2557.2 | 1445.3 | 9.5 | 46 |
| 0.3 | pt | set_2 | 0.00 | 0.56 | 1308.7 | 196.8 | 1.2 | 36 |
| 0.3 | pt | set_3 | 0.00 | 0.48 | 1357.2 | 245.3 | 3.5 | 37 |
| 0.3 | ph | set_1 | 0.00 | 0.28 | 1216.5 | 104.6 | 1.2 | 29 |
| 0.3 | ph | set_2 | 0.00 | 0.24 | 1143.4 | 31.5 | 9.4 | 20 |
| 0.3 | ph | set_3 | 0.00 | 0.32 | 1134.2 | 22.3 | 9.6 | 14 |
| 0.3 | th | set_1 | 0.00 | 0.6 | NA | NA | NA | 54 |
| 0.3 | th | set_2 | 0.00 | 0.52 | 2156.1 | 1044.1 | 1.2 | 45 |
| 0.3 | th | set_3 | 0.00 | 0.52 | 1562.6 | 450.6 | 9.2 | 41 |
| 0.3 | lqp | set_1 | 0.00 | 0.16 | 1127.6 | 15.7 | 2.5 | 7 |
| 0.3 | lqp | set_2 | 0.00 | 0.12 | 1127.9 | 16.0 | 2.2 | 6 |
| 0.3 | lqp | set_3 | 0.00 | 0.12 | 1125.4 | 13.5 | 7.8 | 5 |
| 0.3 | lqt | set_1 | 0.00 | 0.6 | NA | NA | NA | 52 |
| 0.3 | lqt | set_2 | 0.00 | 0.48 | 1633.2 | 521.3 | 4.1 | 42 |
| 0.3 | lqt | set_3 | 0.00 | 0.52 | 1358.5 | 246.5 | 1.9 | 37 |
| 0.3 | lqh | set_1 | 0.00 | 0.28 | 1155.5 | 43.6 | 2.2 | 22 |
| 0.3 | lqh | set_2 | 0.00 | 0.24 | 1152.9 | 41.0 | 8.3 | 21 |
| 0.3 | lqh | set_3 | 0.00 | 0.32 | 1137.1 | 25.1 | 2.3 | 14 |
| 0.3 | lpt | set_1 | 0.00 | 0.52 | 2557.3 | 1445.4 | 9.0 | 46 |
| 0.3 | lpt | set_2 | 0.00 | 0.56 | 1340.3 | 228.3 | 1.7 | 37 |
| 0.3 | lpt | set_3 | 0.00 | 0.48 | 1357.4 | 245.5 | 3.2 | 37 |
| 0.3 | lph | set_1 | 0.00 | 0.28 | 1216.6 | 104.7 | 1.2 | 29 |
| 0.3 | lph | set_2 | 0.00 | 0.24 | 1149.5 | 37.6 | 4.6 | 21 |
| 0.3 | lph | set_3 | 0.00 | 0.32 | 1134.2 | 22.3 | 9.7 | 14 |
| 0.3 | qpt | set_1 | 0.00 | 0.6 | 2556.9 | 1445.0 | 1.1 | 46 |
| 0.3 | qpt | set_2 | 0.00 | 0.52 | 1421.9 | 310.0 | 3.2 | 39 |
| 0.3 | qpt | set_3 | 0.00 | 0.52 | 1394.1 | 282.2 | 3.5 | 38 |
| 0.3 | qph | set_1 | 0.00 | 0.28 | 1194.0 | 82.1 | 1.0 | 27 |
| 0.3 | qph | set_2 | 0.00 | 0.24 | 1123.1 | 11.2 | 2.5 | 16 |
| 0.3 | qph | set_3 | 0.00 | 0.32 | 1130.4 | 18.5 | 6.5 | 13 |
| 0.3 | qth | set_1 | 0.00 | 0.6 | NA | NA | NA | 52 |
| 0.3 | qth | set_2 | 0.00 | 0.52 | 2564.1 | 1452.2 | 3.0 | 46 |
| 0.3 | qth | set_3 | 0.00 | 0.52 | 1766.8 | 654.8 | 4.2 | 43 |
| 0.3 | pth | set_1 | 0.00 | 0.6 | NA | NA | NA | 51 |
| 0.3 | pth | set_2 | 0.00 | 0.52 | 1629.3 | 517.3 | 3.0 | 42 |
| 0.3 | pth | set_3 | 0.00 | 0.52 | 1765.2 | 653.3 | 9.4 | 43 |
| 0.3 | lqpt | set_1 | 0.00 | 0.6 | 2556.8 | 1444.9 | 1.1 | 46 |
| 0.3 | lqpt | set_2 | 0.00 | 0.52 | 1377.4 | 265.5 | 1.4 | 38 |
| 0.3 | lqpt | set_3 | 0.00 | 0.52 | 1394.2 | 282.3 | 3.3 | 38 |
| 0.3 | lqph | set_1 | 0.00 | 0.28 | 1184.1 | 72.2 | 1.4 | 26 |
| 0.3 | lqph | set_2 | 0.00 | 0.24 | 1132.6 | 20.7 | 2.1 | 18 |
| 0.3 | lqph | set_3 | 0.00 | 0.32 | 1130.3 | 18.4 | 6.8 | 13 |
| 0.3 | lqth | set_1 | 0.00 | 0.6 | NA | NA | NA | 54 |
| 0.3 | lqth | set_2 | 0.00 | 0.52 | 3380.8 | 2268.8 | 0.0 | 47 |
| 0.3 | lqth | set_3 | 0.00 | 0.52 | 1650.0 | 538.0 | 9.9 | 42 |
| 0.3 | lpth | set_1 | 0.00 | 0.6 | NA | NA | NA | 51 |
| 0.3 | lpth | set_2 | 0.00 | 0.52 | 1629.3 | 517.3 | 3.0 | 42 |
| 0.3 | lpth | set_3 | 0.00 | 0.52 | 1765.2 | 653.3 | 9.4 | 43 |
| 0.3 | lqpth | set_1 | 0.00 | 0.6 | NA | NA | NA | 52 |
| 0.3 | lqpth | set_2 | 0.00 | 0.52 | 1745.7 | 633.8 | 1.6 | 43 |
| 0.3 | lqpth | set_3 | 0.00 | 0.52 | 1561.0 | 449.1 | 2.0 | 41 |
| 0.4 | l | set_1 | 0.00 | 0.16 | 1173.6 | 61.7 | 2.6 | 5 |
| 0.4 | l | set_2 | 0.00 | 0.24 | 1178.9 | 67.0 | 1.9 | 4 |
| 0.4 | l | set_3 | 0.00 | 0.2 | 1181.6 | 69.7 | 4.9 | 3 |
| 0.4 | q | set_1 | 0.00 | 0.32 | 1169.6 | 57.7 | 2.0 | 5 |
| 0.4 | q | set_2 | 0.00 | 0.2 | 1169.6 | 57.6 | 2.0 | 4 |
| 0.4 | q | set_3 | 0.00 | 0.28 | 1186.1 | 74.2 | 5.1 | 3 |
| 0.4 | p | set_1 | 0.00 | 0.24 | 1147.7 | 35.8 | 1.1 | 5 |
| 0.4 | p | set_2 | 0.00 | 0.24 | 1158.8 | 46.9 | 4.4 | 4 |
| 0.4 | p | set_3 | 0.00 | 0.12 | 1177.8 | 65.9 | 3.3 | 3 |
| 0.4 | t | set_1 | 0.00 | 0.48 | 1290.9 | 179.0 | 9.1 | 35 |
| 0.4 | t | set_2 | 0.00 | 0.44 | 1204.0 | 92.0 | 7.0 | 30 |
| 0.4 | t | set_3 | 0.00 | 0.44 | 1184.5 | 72.5 | 1.2 | 27 |
| 0.4 | h | set_1 | 0.00 | 0.24 | 1158.6 | 46.6 | 5.0 | 22 |
| 0.4 | h | set_2 | 0.00 | 0.2 | 1133.0 | 21.1 | 1.8 | 17 |
| 0.4 | h | set_3 | 0.00 | 0.32 | 1134.6 | 22.6 | 8.2 | 13 |
| 0.4 | lq | set_1 | 0.00 | 0.16 | 1129.1 | 17.2 | 1.2 | 6 |
| 0.4 | lq | set_2 | 0.00 | 0.12 | 1128.6 | 16.7 | 1.6 | 5 |
| 0.4 | lq | set_3 | 0.00 | 0.12 | 1126.4 | 14.5 | 4.9 | 4 |
| 0.4 | lp | set_1 | 0.00 | 0.24 | 1144.1 | 32.2 | 6.9 | 5 |
| 0.4 | lp | set_2 | 0.00 | 0.24 | 1163.6 | 51.6 | 4.2 | 6 |
| 0.4 | lp | set_3 | 0.00 | 0.16 | 1165.3 | 53.3 | 1.7 | 4 |
| 0.4 | lt | set_1 | 0.00 | 0.48 | 1319.5 | 207.6 | 5.7 | 36 |
| 0.4 | lt | set_2 | 0.00 | 0.44 | 1235.7 | 123.8 | 8.9 | 32 |
| 0.4 | lt | set_3 | 0.00 | 0.4 | 1186.1 | 74.2 | 5.2 | 27 |
| 0.4 | lh | set_1 | 0.00 | 0.24 | 1152.5 | 40.6 | 1.0 | 21 |
| 0.4 | lh | set_2 | 0.00 | 0.2 | 1128.6 | 16.7 | 1.6 | 16 |
| 0.4 | lh | set_3 | 0.00 | 0.32 | 1127.6 | 15.7 | 2.6 | 11 |
| 0.4 | qp | set_1 | 0.00 | 0.12 | 1129.4 | 17.5 | 1.0 | 7 |
| 0.4 | qp | set_2 | 0.00 | 0.12 | 1127.0 | 15.0 | 3.7 | 5 |
| 0.4 | qp | set_3 | 0.00 | 0.12 | 1124.6 | 12.7 | 1.2 | 4 |
| 0.4 | qt | set_1 | 0.00 | 0.56 | 1319.0 | 207.1 | 7.3 | 36 |
| 0.4 | qt | set_2 | 0.00 | 0.48 | 1235.4 | 123.5 | 1.0 | 32 |
| 0.4 | qt | set_3 | 0.00 | 0.48 | 1186.6 | 74.7 | 4.2 | 27 |
| 0.4 | qh | set_1 | 0.00 | 0.28 | 1147.5 | 35.6 | 1.2 | 20 |
| 0.4 | qh | set_2 | 0.00 | 0.2 | 1120.6 | 8.7 | 8.9 | 14 |
| 0.4 | qh | set_3 | 0.00 | 0.28 | 1128.7 | 16.8 | 1.5 | 11 |
| 0.4 | pt | set_1 | 0.00 | 0.48 | 1316.7 | 204.8 | 2.3 | 36 |
| 0.4 | pt | set_2 | 0.00 | 0.44 | 1217.5 | 105.6 | 8.1 | 31 |
| 0.4 | pt | set_3 | 0.00 | 0.4 | 1219.4 | 107.5 | 3.1 | 30 |
| 0.4 | ph | set_1 | 0.00 | 0.24 | 1132.5 | 20.6 | 2.3 | 18 |
| 0.4 | ph | set_2 | 0.00 | 0.2 | 1116.8 | 4.8 | 6.2 | 14 |
| 0.4 | ph | set_3 | 0.00 | 0.32 | 1128.3 | 16.3 | 1.9 | 12 |
| 0.4 | th | set_1 | 0.00 | 0.52 | 1428.2 | 316.2 | 1.5 | 39 |
| 0.4 | th | set_2 | 0.00 | 0.52 | 1233.1 | 121.2 | 3.3 | 32 |
| 0.4 | th | set_3 | 0.00 | 0.4 | 1235.0 | 123.1 | 1.3 | 31 |
| 0.4 | lqp | set_1 | 0.00 | 0.12 | 1129.4 | 17.5 | 1.1 | 7 |
| 0.4 | lqp | set_2 | 0.00 | 0.12 | 1126.5 | 14.6 | 4.8 | 5 |
| 0.4 | lqp | set_3 | 0.00 | 0.12 | 1124.1 | 12.2 | 1.5 | 4 |
| 0.4 | lqt | set_1 | 0.00 | 0.56 | 1350.9 | 239.0 | 8.9 | 37 |
| 0.4 | lqt | set_2 | 0.00 | 0.48 | 1253.9 | 142.0 | 1.0 | 33 |
| 0.4 | lqt | set_3 | 0.00 | 0.48 | 1221.3 | 109.4 | 1.2 | 30 |
| 0.4 | lqh | set_1 | 0.00 | 0.28 | 1147.4 | 35.5 | 1.3 | 20 |
| 0.4 | lqh | set_2 | 0.00 | 0.2 | 1129.1 | 17.2 | 1.3 | 16 |
| 0.4 | lqh | set_3 | 0.00 | 0.32 | 1140.0 | 28.1 | 5.5 | 14 |
| 0.4 | lpt | set_1 | 0.00 | 0.48 | 1316.7 | 204.8 | 2.3 | 36 |
| 0.4 | lpt | set_2 | 0.00 | 0.44 | 1217.5 | 105.6 | 8.1 | 31 |
| 0.4 | lpt | set_3 | 0.00 | 0.36 | 1219.5 | 107.6 | 3.0 | 30 |
| 0.4 | lph | set_1 | 0.00 | 0.24 | 1155.7 | 43.8 | 2.1 | 22 |
| 0.4 | lph | set_2 | 0.00 | 0.2 | 1116.8 | 4.8 | 6.3 | 14 |
| 0.4 | lph | set_3 | 0.00 | 0.32 | 1128.3 | 16.4 | 1.9 | 12 |
| 0.4 | qpt | set_1 | 0.00 | 0.56 | 1429.8 | 317.8 | 6.7 | 39 |
| 0.4 | qpt | set_2 | 0.00 | 0.48 | 1233.5 | 121.6 | 2.7 | 32 |
| 0.4 | qpt | set_3 | 0.00 | 0.52 | 1219.3 | 107.3 | 3.4 | 30 |
| 0.4 | qph | set_1 | 0.00 | 0.28 | 1170.8 | 58.8 | 1.1 | 24 |
| 0.4 | qph | set_2 | 0.00 | 0.2 | 1120.9 | 9.0 | 8.0 | 15 |
| 0.4 | qph | set_3 | 0.00 | 0.32 | 1124.7 | 12.8 | 1.1 | 11 |
| 0.4 | qth | set_1 | 0.00 | 0.56 | 1384.1 | 272.1 | 5.7 | 38 |
| 0.4 | qth | set_2 | 0.00 | 0.48 | 1233.5 | 121.6 | 2.7 | 32 |
| 0.4 | qth | set_3 | 0.00 | 0.48 | 1269.8 | 157.9 | 3.6 | 33 |
| 0.4 | pth | set_1 | 0.00 | 0.52 | 1481.5 | 369.5 | 4.0 | 40 |
| 0.4 | pth | set_2 | 0.00 | 0.52 | 1270.4 | 158.5 | 2.7 | 34 |
| 0.4 | pth | set_3 | 0.00 | 0.4 | 1250.0 | 138.1 | 7.4 | 32 |
| 0.4 | lqpt | set_1 | 0.00 | 0.56 | 1347.9 | 236.0 | 4.0 | 37 |
| 0.4 | lqpt | set_2 | 0.00 | 0.48 | 1233.5 | 121.5 | 2.9 | 32 |
| 0.4 | lqpt | set_3 | 0.00 | 0.52 | 1219.1 | 107.1 | 3.8 | 30 |
| 0.4 | lqph | set_1 | 0.00 | 0.28 | 1170.7 | 58.7 | 1.2 | 24 |
| 0.4 | lqph | set_2 | 0.00 | 0.2 | 1116.6 | 4.7 | 6.9 | 14 |
| 0.4 | lqph | set_3 | 0.00 | 0.32 | 1124.7 | 12.8 | 1.1 | 11 |
| 0.4 | lqth | set_1 | 0.00 | 0.56 | 1428.5 | 316.5 | 1.3 | 39 |
| 0.4 | lqth | set_2 | 0.00 | 0.48 | 1271.6 | 159.7 | 1.4 | 34 |
| 0.4 | lqth | set_3 | 0.00 | 0.48 | 1313.2 | 201.3 | 1.3 | 35 |
| 0.4 | lpth | set_1 | 0.00 | 0.52 | 1481.5 | 369.5 | 4.0 | 40 |
| 0.4 | lpth | set_2 | 0.00 | 0.52 | 1270.4 | 158.5 | 2.7 | 34 |
| 0.4 | lpth | set_3 | 0.00 | 0.4 | 1250.0 | 138.1 | 7.3 | 32 |
| 0.4 | lqpth | set_1 | 0.00 | 0.52 | 1549.2 | 437.3 | 7.9 | 41 |
| 0.4 | lqpth | set_2 | 0.00 | 0.52 | 1270.3 | 158.4 | 2.9 | 34 |
| 0.4 | lqpth | set_3 | 0.00 | 0.44 | 1219.2 | 107.3 | 3.6 | 30 |
| 0.5 | l | set_1 | 0.00 | 0.2 | 1173.7 | 61.8 | 2.7 | 5 |
| 0.5 | l | set_2 | 0.00 | 0.2 | 1178.9 | 67.0 | 2.0 | 4 |
| 0.5 | l | set_3 | 0.00 | 0.2 | 1181.6 | 69.7 | 5.2 | 3 |
| 0.5 | q | set_1 | 0.00 | 0.32 | 1169.7 | 57.7 | 2.0 | 5 |
| 0.5 | q | set_2 | 0.00 | 0.2 | 1169.6 | 57.7 | 2.1 | 4 |
| 0.5 | q | set_3 | 0.00 | 0.28 | 1186.2 | 74.2 | 5.4 | 3 |
| 0.5 | p | set_1 | 0.00 | 0.24 | 1148.0 | 36.1 | 1.0 | 5 |
| 0.5 | p | set_2 | 0.00 | 0.24 | 1160.1 | 48.1 | 2.5 | 4 |
| 0.5 | p | set_3 | 0.00 | 0.12 | 1178.7 | 66.8 | 2.2 | 3 |
| 0.5 | t | set_1 | 0.00 | 0.44 | 1239.5 | 127.6 | 1.4 | 32 |
| 0.5 | t | set_2 | 0.00 | 0.32 | 1187.3 | 75.4 | 3.1 | 28 |
| 0.5 | t | set_3 | 0.00 | 0.48 | 1157.7 | 45.7 | 8.4 | 23 |
| 0.5 | h | set_1 | 0.00 | 0.24 | 1137.7 | 25.8 | 1.8 | 18 |
| 0.5 | h | set_2 | 0.00 | 0.2 | 1130.4 | 18.4 | 7.2 | 16 |
| 0.5 | h | set_3 | 0.00 | 0.24 | 1125.2 | 13.2 | 9.7 | 10 |
| 0.5 | lq | set_1 | 0.00 | 0.12 | 1129.2 | 17.3 | 1.2 | 6 |
| 0.5 | lq | set_2 | 0.00 | 0.12 | 1128.7 | 16.7 | 1.6 | 5 |
| 0.5 | lq | set_3 | 0.00 | 0.12 | 1126.4 | 14.5 | 5.1 | 4 |
| 0.5 | lp | set_1 | 0.00 | 0.24 | 1144.5 | 32.6 | 6.0 | 5 |
| 0.5 | lp | set_2 | 0.00 | 0.24 | 1162.2 | 50.3 | 8.8 | 5 |
| 0.5 | lp | set_3 | 0.00 | 0.16 | 1166.4 | 54.5 | 1.0 | 4 |
| 0.5 | lt | set_1 | 0.00 | 0.44 | 1187.5 | 75.6 | 2.8 | 28 |
| 0.5 | lt | set_2 | 0.00 | 0.4 | 1170.0 | 58.1 | 1.8 | 26 |
| 0.5 | lt | set_3 | 0.00 | 0.4 | 1154.3 | 42.3 | 4.7 | 22 |
| 0.5 | lh | set_1 | 0.00 | 0.24 | 1128.5 | 16.6 | 1.8 | 16 |
| 0.5 | lh | set_2 | 0.00 | 0.2 | 1114.7 | 2.7 | 1.8 | 12 |
| 0.5 | lh | set_3 | 0.00 | 0.28 | 1121.9 | 10.0 | 4.8 | 9 |
| 0.5 | qp | set_1 | 0.00 | 0.12 | 1125.1 | 13.1 | 1.0 | 5 |
| 0.5 | qp | set_2 | 0.00 | 0.12 | 1127.2 | 15.3 | 3.5 | 5 |
| 0.5 | qp | set_3 | 0.00 | 0.12 | 1124.8 | 12.9 | 1.1 | 4 |
| 0.5 | qt | set_1 | 0.00 | 0.48 | 1241.7 | 129.8 | 4.8 | 32 |
| 0.5 | qt | set_2 | 0.00 | 0.48 | 1179.4 | 67.5 | 1.6 | 27 |
| 0.5 | qt | set_3 | 0.00 | 0.48 | 1168.7 | 56.7 | 3.5 | 24 |
| 0.5 | qh | set_1 | 0.00 | 0.24 | 1120.9 | 8.9 | 8.4 | 14 |
| 0.5 | qh | set_2 | 0.00 | 0.2 | 1119.2 | 7.3 | 1.9 | 13 |
| 0.5 | qh | set_3 | 0.00 | 0.28 | 1126.3 | 14.3 | 5.6 | 10 |
| 0.5 | pt | set_1 | 0.00 | 0.44 | 1197.0 | 85.1 | 2.4 | 29 |
| 0.5 | pt | set_2 | 0.00 | 0.4 | 1168.4 | 56.5 | 3.9 | 26 |
| 0.5 | pt | set_3 | 0.00 | 0.44 | 1167.7 | 55.8 | 5.7 | 24 |
| 0.5 | ph | set_1 | 0.00 | 0.24 | 1147.2 | 35.3 | 1.6 | 20 |
| 0.5 | ph | set_2 | 0.00 | 0.2 | 1114.7 | 2.8 | 1.8 | 13 |
| 0.5 | ph | set_3 | 0.00 | 0.24 | 1125.6 | 13.7 | 7.8 | 11 |
| 0.5 | th | set_1 | 0.00 | 0.48 | 1300.2 | 188.3 | 9.8 | 35 |
| 0.5 | th | set_2 | 0.00 | 0.36 | 1200.2 | 88.3 | 4.9 | 29 |
| 0.5 | th | set_3 | 0.00 | 0.36 | 1196.3 | 84.3 | 3.5 | 27 |
| 0.5 | lqp | set_1 | 0.00 | 0.12 | 1124.8 | 12.9 | 1.1 | 5 |
| 0.5 | lqp | set_2 | 0.00 | 0.12 | 1126.7 | 14.8 | 4.5 | 5 |
| 0.5 | lqp | set_3 | 0.00 | 0.12 | 1124.3 | 12.4 | 1.5 | 4 |
| 0.5 | lqt | set_1 | 0.00 | 0.48 | 1199.8 | 87.9 | 6.1 | 29 |
| 0.5 | lqt | set_2 | 0.00 | 0.48 | 1170.8 | 58.9 | 1.2 | 26 |
| 0.5 | lqt | set_3 | 0.00 | 0.48 | 1186.8 | 74.9 | 4.1 | 26 |
| 0.5 | lqh | set_1 | 0.00 | 0.24 | 1120.8 | 8.9 | 8.8 | 14 |
| 0.5 | lqh | set_2 | 0.00 | 0.2 | 1131.6 | 19.7 | 3.9 | 16 |
| 0.5 | lqh | set_3 | 0.00 | 0.28 | 1137.4 | 25.5 | 2.2 | 13 |
| 0.5 | lpt | set_1 | 0.00 | 0.44 | 1209.8 | 97.9 | 4.2 | 30 |
| 0.5 | lpt | set_2 | 0.00 | 0.4 | 1168.4 | 56.5 | 4.0 | 26 |
| 0.5 | lpt | set_3 | 0.00 | 0.44 | 1175.9 | 63.9 | 9.8 | 25 |
| 0.5 | lph | set_1 | 0.00 | 0.24 | 1147.2 | 35.2 | 1.6 | 20 |
| 0.5 | lph | set_2 | 0.00 | 0.2 | 1122.8 | 10.9 | 3.2 | 15 |
| 0.5 | lph | set_3 | 0.00 | 0.24 | 1125.6 | 13.7 | 7.9 | 11 |
| 0.5 | qpt | set_1 | 0.00 | 0.44 | 1210.2 | 98.2 | 3.5 | 30 |
| 0.5 | qpt | set_2 | 0.00 | 0.48 | 1189.0 | 77.1 | 1.3 | 28 |
| 0.5 | qpt | set_3 | 0.00 | 0.48 | 1176.3 | 64.4 | 7.9 | 25 |
| 0.5 | qph | set_1 | 0.00 | 0.24 | 1147.2 | 35.2 | 1.6 | 20 |
| 0.5 | qph | set_2 | 0.00 | 0.2 | 1118.8 | 6.9 | 2.3 | 14 |
| 0.5 | qph | set_3 | 0.00 | 0.24 | 1125.7 | 13.7 | 7.8 | 11 |
| 0.5 | qth | set_1 | 0.00 | 0.48 | 1239.5 | 127.6 | 1.4 | 32 |
| 0.5 | qth | set_2 | 0.00 | 0.4 | 1189.6 | 77.7 | 1.0 | 28 |
| 0.5 | qth | set_3 | 0.00 | 0.44 | 1179.0 | 67.0 | 2.0 | 25 |
| 0.5 | pth | set_1 | 0.00 | 0.48 | 1299.7 | 187.8 | 1.2 | 35 |
| 0.5 | pth | set_2 | 0.00 | 0.36 | 1199.7 | 87.7 | 6.7 | 29 |
| 0.5 | pth | set_3 | 0.00 | 0.36 | 1177.7 | 65.8 | 3.8 | 25 |
| 0.5 | lqpt | set_1 | 0.00 | 0.44 | 1210.1 | 98.1 | 3.7 | 30 |
| 0.5 | lqpt | set_2 | 0.00 | 0.48 | 1189.0 | 77.1 | 1.3 | 28 |
| 0.5 | lqpt | set_3 | 0.00 | 0.48 | 1176.3 | 64.4 | 7.9 | 25 |
| 0.5 | lqph | set_1 | 0.00 | 0.24 | 1153.6 | 41.7 | 6.7 | 21 |
| 0.5 | lqph | set_2 | 0.00 | 0.2 | 1126.9 | 15.0 | 4.1 | 16 |
| 0.5 | lqph | set_3 | 0.00 | 0.24 | 1136.9 | 25.0 | 2.8 | 14 |
| 0.5 | lqth | set_1 | 0.00 | 0.48 | 1257.7 | 145.8 | 1.6 | 33 |
| 0.5 | lqth | set_2 | 0.00 | 0.4 | 1214.1 | 102.2 | 4.8 | 30 |
| 0.5 | lqth | set_3 | 0.00 | 0.44 | 1187.7 | 75.8 | 2.6 | 26 |
| 0.5 | lpth | set_1 | 0.00 | 0.48 | 1299.7 | 187.8 | 1.2 | 35 |
| 0.5 | lpth | set_2 | 0.00 | 0.36 | 1199.7 | 87.7 | 6.7 | 29 |
| 0.5 | lpth | set_3 | 0.00 | 0.36 | 1177.7 | 65.8 | 3.9 | 25 |
| 0.5 | lqpth | set_1 | 0.00 | 0.48 | 1326.6 | 214.7 | 1.8 | 36 |
| 0.5 | lqpth | set_2 | 0.00 | 0.32 | 1188.2 | 76.2 | 2.1 | 28 |
| 0.5 | lqpth | set_3 | 0.00 | 0.44 | 1177.6 | 65.7 | 4.1 | 25 |
| 0.6 | l | set_1 | 0.00 | 0.2 | 1173.8 | 61.9 | 2.7 | 5 |
| 0.6 | l | set_2 | 0.00 | 0.2 | 1179.0 | 67.1 | 2.1 | 4 |
| 0.6 | l | set_3 | 0.00 | 0.2 | 1181.6 | 69.7 | 5.5 | 3 |
| 0.6 | q | set_1 | 0.00 | 0.32 | 1169.7 | 57.8 | 2.1 | 5 |
| 0.6 | q | set_2 | 0.00 | 0.2 | 1169.7 | 57.8 | 2.2 | 4 |
| 0.6 | q | set_3 | 0.00 | 0.28 | 1186.2 | 74.2 | 5.8 | 3 |
| 0.6 | p | set_1 | 0.00 | 0.24 | 1145.9 | 34.0 | 3.2 | 4 |
| 0.6 | p | set_2 | 0.00 | 0.24 | 1161.5 | 49.5 | 1.3 | 4 |
| 0.6 | p | set_3 | 0.00 | 0.12 | 1179.8 | 67.9 | 1.4 | 3 |
| 0.6 | t | set_1 | 0.00 | 0.4 | 1182.3 | 70.4 | 4.0 | 27 |
| 0.6 | t | set_2 | 0.00 | 0.32 | 1156.1 | 44.2 | 1.9 | 24 |
| 0.6 | t | set_3 | 0.00 | 0.44 | 1163.1 | 51.2 | 5.8 | 23 |
| 0.6 | h | set_1 | 0.00 | 0.24 | 1126.6 | 14.6 | 5.1 | 15 |
| 0.6 | h | set_2 | 0.00 | 0.2 | 1124.1 | 12.2 | 1.7 | 14 |
| 0.6 | h | set_3 | 0.00 | 0.24 | 1129.5 | 17.6 | 1.1 | 11 |
| 0.6 | lq | set_1 | 0.00 | 0.12 | 1129.3 | 17.4 | 1.3 | 6 |
| 0.6 | lq | set_2 | 0.00 | 0.12 | 1128.8 | 16.8 | 1.7 | 5 |
| 0.6 | lq | set_3 | 0.00 | 0.12 | 1126.5 | 14.6 | 5.2 | 4 |
| 0.6 | lp | set_1 | 0.00 | 0.24 | 1145.0 | 33.0 | 5.2 | 5 |
| 0.6 | lp | set_2 | 0.00 | 0.24 | 1163.6 | 51.6 | 4.7 | 5 |
| 0.6 | lp | set_3 | 0.00 | 0.2 | 1168.0 | 56.1 | 5.2 | 4 |
| 0.6 | lt | set_1 | 0.00 | 0.44 | 1138.9 | 27.0 | 1.0 | 21 |
| 0.6 | lt | set_2 | 0.00 | 0.4 | 1134.0 | 22.1 | 1.2 | 20 |
| 0.6 | lt | set_3 | 0.00 | 0.44 | 1133.5 | 21.5 | 1.6 | 17 |
| 0.6 | lh | set_1 | 0.00 | 0.24 | 1130.7 | 18.8 | 6.5 | 16 |
| 0.6 | lh | set_2 | 0.00 | 0.2 | 1124.9 | 13.0 | 1.1 | 14 |
| 0.6 | lh | set_3 | 0.00 | 0.24 | 1122.7 | 10.8 | 3.5 | 9 |
| 0.6 | qp | set_1 | 0.00 | 0.12 | 1125.3 | 13.4 | 9.5 | 5 |
| 0.6 | qp | set_2 | 0.00 | 0.12 | 1130.1 | 18.1 | 8.9 | 6 |
| 0.6 | qp | set_3 | 0.00 | 0.12 | 1125.0 | 13.1 | 1.1 | 4 |
| 0.6 | qt | set_1 | 0.00 | 0.48 | 1152.9 | 40.9 | 1.0 | 23 |
| 0.6 | qt | set_2 | 0.00 | 0.48 | 1140.3 | 28.4 | 5.4 | 21 |
| 0.6 | qt | set_3 | 0.00 | 0.48 | 1143.7 | 31.8 | 9.7 | 19 |
| 0.6 | qh | set_1 | 0.00 | 0.24 | 1115.7 | 3.7 | 1.2 | 12 |
| 0.6 | qh | set_2 | 0.00 | 0.2 | 1114.6 | 2.7 | 2.0 | 11 |
| 0.6 | qh | set_3 | 0.00 | 0.2 | 1127.1 | 15.2 | 3.9 | 10 |
| 0.6 | pt | set_1 | 0.00 | 0.44 | 1157.7 | 45.7 | 9.1 | 24 |
| 0.6 | pt | set_2 | 0.00 | 0.44 | 1138.2 | 26.2 | 1.5 | 21 |
| 0.6 | pt | set_3 | 0.00 | 0.44 | 1142.1 | 30.2 | 2.2 | 19 |
| 0.6 | ph | set_1 | 0.00 | 0.24 | 1135.4 | 23.5 | 6.3 | 17 |
| 0.6 | ph | set_2 | 0.00 | 0.2 | 1131.8 | 19.9 | 3.8 | 16 |
| 0.6 | ph | set_3 | 0.00 | 0.24 | 1125.2 | 13.3 | 1.0 | 10 |
| 0.6 | th | set_1 | 0.00 | 0.44 | 1166.1 | 54.2 | 1.3 | 25 |
| 0.6 | th | set_2 | 0.00 | 0.32 | 1169.0 | 57.1 | 3.1 | 25 |
| 0.6 | th | set_3 | 0.00 | 0.4 | 1151.9 | 40.0 | 1.6 | 20 |
| 0.6 | lqp | set_1 | 0.00 | 0.12 | 1125.1 | 13.2 | 1.1 | 5 |
| 0.6 | lqp | set_2 | 0.00 | 0.12 | 1129.6 | 17.6 | 1.1 | 6 |
| 0.6 | lqp | set_3 | 0.00 | 0.12 | 1124.5 | 12.6 | 1.4 | 4 |
| 0.6 | lqt | set_1 | 0.00 | 0.44 | 1161.7 | 49.8 | 1.2 | 24 |
| 0.6 | lqt | set_2 | 0.00 | 0.48 | 1155.3 | 43.3 | 3.0 | 23 |
| 0.6 | lqt | set_3 | 0.00 | 0.48 | 1145.8 | 33.9 | 3.4 | 19 |
| 0.6 | lqh | set_1 | 0.00 | 0.24 | 1127.3 | 15.4 | 3.6 | 15 |
| 0.6 | lqh | set_2 | 0.00 | 0.2 | 1125.9 | 13.9 | 7.4 | 14 |
| 0.6 | lqh | set_3 | 0.00 | 0.2 | 1131.4 | 19.5 | 4.6 | 11 |
| 0.6 | lpt | set_1 | 0.00 | 0.44 | 1165.9 | 53.9 | 1.5 | 25 |
| 0.6 | lpt | set_2 | 0.00 | 0.44 | 1151.5 | 39.6 | 2.0 | 23 |
| 0.6 | lpt | set_3 | 0.00 | 0.44 | 1153.8 | 41.9 | 6.3 | 21 |
| 0.6 | lph | set_1 | 0.00 | 0.24 | 1145.6 | 33.6 | 3.9 | 19 |
| 0.6 | lph | set_2 | 0.00 | 0.2 | 1131.3 | 19.4 | 4.9 | 16 |
| 0.6 | lph | set_3 | 0.00 | 0.24 | 1125.2 | 13.3 | 1.0 | 10 |
| 0.6 | qpt | set_1 | 0.00 | 0.44 | 1167.0 | 55.1 | 8.6 | 25 |
| 0.6 | qpt | set_2 | 0.00 | 0.44 | 1145.8 | 33.8 | 3.6 | 22 |
| 0.6 | qpt | set_3 | 0.00 | 0.48 | 1149.0 | 37.1 | 7.0 | 20 |
| 0.6 | qph | set_1 | 0.00 | 0.24 | 1135.8 | 23.8 | 5.3 | 17 |
| 0.6 | qph | set_2 | 0.00 | 0.2 | 1132.2 | 20.2 | 3.2 | 16 |
| 0.6 | qph | set_3 | 0.00 | 0.2 | 1133.3 | 21.4 | 1.8 | 12 |
| 0.6 | qth | set_1 | 0.00 | 0.36 | 1185.2 | 73.3 | 9.9 | 27 |
| 0.6 | qth | set_2 | 0.00 | 0.28 | 1140.9 | 29.0 | 4.0 | 21 |
| 0.6 | qth | set_3 | 0.00 | 0.48 | 1141.8 | 29.9 | 2.6 | 18 |
| 0.6 | pth | set_1 | 0.00 | 0.44 | 1174.9 | 63.0 | 1.6 | 26 |
| 0.6 | pth | set_2 | 0.00 | 0.32 | 1161.1 | 49.2 | 1.6 | 24 |
| 0.6 | pth | set_3 | 0.00 | 0.4 | 1141.2 | 29.2 | 3.6 | 18 |
| 0.6 | lqpt | set_1 | 0.00 | 0.44 | 1167.1 | 55.2 | 8.3 | 25 |
| 0.6 | lqpt | set_2 | 0.00 | 0.44 | 1145.7 | 33.8 | 3.6 | 22 |
| 0.6 | lqpt | set_3 | 0.00 | 0.48 | 1149.1 | 37.2 | 6.8 | 20 |
| 0.6 | lqph | set_1 | 0.00 | 0.24 | 1136.1 | 24.2 | 4.5 | 17 |
| 0.6 | lqph | set_2 | 0.00 | 0.2 | 1137.6 | 25.6 | 2.2 | 17 |
| 0.6 | lqph | set_3 | 0.00 | 0.2 | 1133.1 | 21.2 | 2.0 | 12 |
| 0.6 | lqth | set_1 | 0.00 | 0.36 | 1195.8 | 83.8 | 5.0 | 28 |
| 0.6 | lqth | set_2 | 0.00 | 0.28 | 1169.9 | 57.9 | 2.1 | 25 |
| 0.6 | lqth | set_3 | 0.00 | 0.48 | 1146.9 | 35.0 | 2.0 | 19 |
| 0.6 | lpth | set_1 | 0.00 | 0.44 | 1174.9 | 63.0 | 1.7 | 26 |
| 0.6 | lpth | set_2 | 0.00 | 0.32 | 1161.1 | 49.2 | 1.6 | 24 |
| 0.6 | lpth | set_3 | 0.00 | 0.4 | 1141.2 | 29.2 | 3.6 | 18 |
| 0.6 | lqpth | set_1 | 0.00 | 0.4 | 1195.4 | 83.5 | 6.0 | 28 |
| 0.6 | lqpth | set_2 | 0.00 | 0.32 | 1169.3 | 57.3 | 2.9 | 25 |
| 0.6 | lqpth | set_3 | 0.00 | 0.44 | 1158.1 | 46.2 | 7.6 | 21 |
| 0.7 | l | set_1 | 0.00 | 0.2 | 1173.9 | 62.0 | 2.8 | 5 |
| 0.7 | l | set_2 | 0.00 | 0.2 | 1176.7 | 64.7 | 7.1 | 3 |
| 0.7 | l | set_3 | 0.00 | 0.2 | 1181.6 | 69.7 | 5.9 | 3 |
| 0.7 | q | set_1 | 0.00 | 0.32 | 1169.8 | 57.9 | 2.2 | 5 |
| 0.7 | q | set_2 | 0.00 | 0.2 | 1169.8 | 57.8 | 2.2 | 4 |
| 0.7 | q | set_3 | 0.00 | 0.28 | 1186.2 | 74.3 | 6.1 | 3 |
| 0.7 | p | set_1 | 0.00 | 0.24 | 1146.2 | 34.3 | 3.0 | 4 |
| 0.7 | p | set_2 | 0.00 | 0.24 | 1163.0 | 51.1 | 6.7 | 4 |
| 0.7 | p | set_3 | 0.00 | 0.12 | 1181.1 | 69.2 | 7.8 | 3 |
| 0.7 | t | set_1 | 0.00 | 0.4 | 1160.5 | 48.6 | 2.3 | 24 |
| 0.7 | t | set_2 | 0.00 | 0.32 | 1146.3 | 34.4 | 2.7 | 22 |
| 0.7 | t | set_3 | 0.00 | 0.4 | 1161.0 | 49.1 | 1.7 | 22 |
| 0.7 | h | set_1 | 0.00 | 0.24 | 1129.0 | 17.1 | 1.6 | 15 |
| 0.7 | h | set_2 | 0.00 | 0.2 | 1118.9 | 7.0 | 2.5 | 12 |
| 0.7 | h | set_3 | 0.00 | 0.2 | 1127.1 | 15.1 | 4.2 | 10 |
| 0.7 | lq | set_1 | 0.00 | 0.12 | 1129.4 | 17.5 | 1.3 | 6 |
| 0.7 | lq | set_2 | 0.00 | 0.12 | 1128.8 | 16.9 | 1.7 | 5 |
| 0.7 | lq | set_3 | 0.00 | 0.12 | 1126.6 | 14.7 | 5.4 | 4 |
| 0.7 | lp | set_1 | 0.00 | 0.2 | 1145.5 | 33.6 | 4.3 | 5 |
| 0.7 | lp | set_2 | 0.00 | 0.24 | 1165.0 | 53.1 | 2.4 | 5 |
| 0.7 | lp | set_3 | 0.00 | 0.2 | 1169.6 | 57.7 | 2.5 | 4 |
| 0.7 | lt | set_1 | 0.00 | 0.44 | 1133.3 | 21.4 | 1.9 | 19 |
| 0.7 | lt | set_2 | 0.00 | 0.36 | 1128.5 | 16.6 | 2.1 | 18 |
| 0.7 | lt | set_3 | 0.00 | 0.44 | 1133.6 | 21.7 | 1.6 | 16 |
| 0.7 | lh | set_1 | 0.00 | 0.24 | 1128.9 | 17.0 | 1.7 | 15 |
| 0.7 | lh | set_2 | 0.00 | 0.2 | 1119.4 | 7.5 | 1.9 | 12 |
| 0.7 | lh | set_3 | 0.00 | 0.2 | 1129.9 | 18.0 | 1.0 | 11 |
| 0.7 | qp | set_1 | 0.00 | 0.12 | 1125.7 | 13.7 | 8.7 | 5 |
| 0.7 | qp | set_2 | 0.00 | 0.12 | 1133.1 | 21.2 | 2.0 | 7 |
| 0.7 | qp | set_3 | 0.00 | 0.12 | 1125.3 | 13.4 | 1.0 | 4 |
| 0.7 | qt | set_1 | 0.00 | 0.44 | 1138.0 | 26.1 | 1.8 | 20 |
| 0.7 | qt | set_2 | 0.00 | 0.44 | 1127.5 | 15.6 | 3.4 | 18 |
| 0.7 | qt | set_3 | 0.00 | 0.48 | 1132.0 | 20.1 | 3.6 | 16 |
| 0.7 | qh | set_1 | 0.00 | 0.24 | 1118.0 | 6.0 | 4.1 | 12 |
| 0.7 | qh | set_2 | 0.00 | 0.16 | 1114.2 | 2.3 | 2.7 | 10 |
| 0.7 | qh | set_3 | 0.00 | 0.2 | 1121.9 | 9.9 | 5.8 | 8 |
| 0.7 | pt | set_1 | 0.00 | 0.44 | 1135.9 | 23.9 | 5.3 | 20 |
| 0.7 | pt | set_2 | 0.00 | 0.44 | 1131.5 | 19.6 | 4.7 | 19 |
| 0.7 | pt | set_3 | 0.00 | 0.44 | 1131.6 | 19.6 | 4.5 | 16 |
| 0.7 | ph | set_1 | 0.00 | 0.24 | 1124.9 | 12.9 | 1.3 | 14 |
| 0.7 | ph | set_2 | 0.00 | 0.2 | 1122.6 | 10.7 | 4.0 | 13 |
| 0.7 | ph | set_3 | 0.00 | 0.2 | 1127.1 | 15.2 | 4.3 | 10 |
| 0.7 | th | set_1 | 0.00 | 0.4 | 1152.5 | 40.6 | 1.3 | 22 |
| 0.7 | th | set_2 | 0.00 | 0.24 | 1161.1 | 49.2 | 1.7 | 23 |
| 0.7 | th | set_3 | 0.00 | 0.32 | 1142.1 | 30.2 | 2.3 | 17 |
| 0.7 | lqp | set_1 | 0.00 | 0.16 | 1128.0 | 16.1 | 2.7 | 6 |
| 0.7 | lqp | set_2 | 0.00 | 0.16 | 1132.7 | 20.7 | 2.6 | 7 |
| 0.7 | lqp | set_3 | 0.00 | 0.12 | 1124.8 | 12.9 | 1.3 | 4 |
| 0.7 | lqt | set_1 | 0.00 | 0.44 | 1136.2 | 24.3 | 4.5 | 19 |
| 0.7 | lqt | set_2 | 0.00 | 0.36 | 1131.6 | 19.7 | 4.4 | 18 |
| 0.7 | lqt | set_3 | 0.00 | 0.48 | 1136.8 | 24.9 | 3.3 | 16 |
| 0.7 | lqh | set_1 | 0.00 | 0.24 | 1130.5 | 18.5 | 8.1 | 15 |
| 0.7 | lqh | set_2 | 0.00 | 0.16 | 1120.9 | 9.0 | 9.5 | 12 |
| 0.7 | lqh | set_3 | 0.00 | 0.2 | 1129.1 | 17.2 | 1.6 | 10 |
| 0.7 | lpt | set_1 | 0.00 | 0.44 | 1130.4 | 18.5 | 8.4 | 19 |
| 0.7 | lpt | set_2 | 0.00 | 0.44 | 1131.5 | 19.6 | 4.8 | 19 |
| 0.7 | lpt | set_3 | 0.00 | 0.44 | 1131.8 | 19.8 | 4.2 | 16 |
| 0.7 | lph | set_1 | 0.00 | 0.24 | 1133.4 | 21.5 | 1.8 | 16 |
| 0.7 | lph | set_2 | 0.00 | 0.2 | 1122.7 | 10.8 | 3.8 | 13 |
| 0.7 | lph | set_3 | 0.00 | 0.2 | 1127.1 | 15.2 | 4.4 | 10 |
| 0.7 | qpt | set_1 | 0.00 | 0.44 | 1143.9 | 31.9 | 9.9 | 21 |
| 0.7 | qpt | set_2 | 0.00 | 0.44 | 1133.3 | 21.4 | 1.9 | 19 |
| 0.7 | qpt | set_3 | 0.00 | 0.44 | 1138.1 | 26.2 | 1.7 | 17 |
| 0.7 | qph | set_1 | 0.00 | 0.24 | 1115.1 | 3.1 | 1.8 | 11 |
| 0.7 | qph | set_2 | 0.00 | 0.2 | 1124.1 | 12.2 | 1.9 | 13 |
| 0.7 | qph | set_3 | 0.00 | 0.2 | 1125.4 | 13.4 | 1.0 | 9 |
| 0.7 | qth | set_1 | 0.00 | 0.36 | 1146.2 | 34.3 | 3.0 | 21 |
| 0.7 | qth | set_2 | 0.00 | 0.24 | 1136.8 | 24.8 | 3.4 | 19 |
| 0.7 | qth | set_3 | 0.00 | 0.48 | 1121.7 | 9.8 | 6.4 | 12 |
| 0.7 | pth | set_1 | 0.00 | 0.4 | 1146.2 | 34.2 | 3.1 | 21 |
| 0.7 | pth | set_2 | 0.00 | 0.24 | 1141.6 | 29.7 | 3.0 | 20 |
| 0.7 | pth | set_3 | 0.00 | 0.32 | 1129.3 | 17.4 | 1.4 | 14 |
| 0.7 | lqpt | set_1 | 0.00 | 0.44 | 1143.9 | 32.0 | 9.7 | 21 |
| 0.7 | lqpt | set_2 | 0.00 | 0.44 | 1133.3 | 21.4 | 1.9 | 19 |
| 0.7 | lqpt | set_3 | 0.00 | 0.44 | 1138.2 | 26.3 | 1.7 | 17 |
| 0.7 | lqph | set_1 | 0.00 | 0.24 | 1122.0 | 10.1 | 5.6 | 13 |
| 0.7 | lqph | set_2 | 0.00 | 0.2 | 1120.3 | 8.4 | 1.3 | 12 |
| 0.7 | lqph | set_3 | 0.00 | 0.2 | 1122.4 | 10.5 | 4.7 | 8 |
| 0.7 | lqth | set_1 | 0.00 | 0.32 | 1146.1 | 34.2 | 3.3 | 21 |
| 0.7 | lqth | set_2 | 0.00 | 0.24 | 1136.7 | 24.8 | 3.5 | 19 |
| 0.7 | lqth | set_3 | 0.00 | 0.48 | 1133.4 | 21.5 | 1.8 | 15 |
| 0.7 | lpth | set_1 | 0.00 | 0.4 | 1146.2 | 34.2 | 3.2 | 21 |
| 0.7 | lpth | set_2 | 0.00 | 0.24 | 1141.6 | 29.7 | 3.1 | 20 |
| 0.7 | lpth | set_3 | 0.00 | 0.32 | 1129.3 | 17.4 | 1.4 | 14 |
| 0.7 | lqpth | set_1 | 0.00 | 0.36 | 1146.2 | 34.3 | 3.1 | 21 |
| 0.7 | lqpth | set_2 | 0.00 | 0.24 | 1141.6 | 29.6 | 3.2 | 20 |
| 0.7 | lqpth | set_3 | 0.00 | 0.44 | 1133.4 | 21.5 | 1.9 | 15 |
| 0.8 | l | set_1 | 0.00 | 0.2 | 1174.0 | 62.1 | 2.9 | 5 |
| 0.8 | l | set_2 | 0.00 | 0.2 | 1176.7 | 64.8 | 7.6 | 3 |
| 0.8 | l | set_3 | 0.00 | 0.2 | 1181.7 | 69.7 | 6.4 | 3 |
| 0.8 | q | set_1 | 0.00 | 0.32 | 1169.9 | 58.0 | 2.3 | 5 |
| 0.8 | q | set_2 | 0.00 | 0.2 | 1169.9 | 57.9 | 2.3 | 4 |
| 0.8 | q | set_3 | 0.00 | 0.28 | 1186.2 | 74.3 | 6.5 | 3 |
| 0.8 | p | set_1 | 0.00 | 0.24 | 1146.5 | 34.6 | 2.7 | 4 |
| 0.8 | p | set_2 | 0.00 | 0.24 | 1164.6 | 52.7 | 3.1 | 4 |
| 0.8 | p | set_3 | 0.00 | 0.12 | 1182.6 | 70.6 | 4.0 | 3 |
| 0.8 | t | set_1 | 0.00 | 0.4 | 1150.9 | 39.0 | 3.0 | 22 |
| 0.8 | t | set_2 | 0.00 | 0.32 | 1150.9 | 39.0 | 3.0 | 22 |
| 0.8 | t | set_3 | 0.00 | 0.4 | 1151.9 | 40.0 | 1.8 | 20 |
| 0.8 | h | set_1 | 0.00 | 0.24 | 1131.7 | 19.8 | 4.5 | 15 |
| 0.8 | h | set_2 | 0.00 | 0.2 | 1124.9 | 13.0 | 1.3 | 13 |
| 0.8 | h | set_3 | 0.00 | 0.16 | 1131.4 | 19.4 | 5.4 | 11 |
| 0.8 | lq | set_1 | 0.00 | 0.12 | 1129.5 | 17.6 | 1.3 | 6 |
| 0.8 | lq | set_2 | 0.00 | 0.12 | 1128.9 | 17.0 | 1.8 | 5 |
| 0.8 | lq | set_3 | 0.00 | 0.12 | 1126.7 | 14.8 | 5.5 | 4 |
| 0.8 | lp | set_1 | 0.00 | 0.24 | 1146.0 | 34.1 | 3.5 | 5 |
| 0.8 | lp | set_2 | 0.00 | 0.24 | 1166.7 | 54.8 | 1.1 | 5 |
| 0.8 | lp | set_3 | 0.00 | 0.2 | 1171.3 | 59.3 | 1.1 | 4 |
| 0.8 | lt | set_1 | 0.00 | 0.4 | 1124.7 | 12.7 | 1.5 | 16 |
| 0.8 | lt | set_2 | 0.00 | 0.36 | 1119.6 | 7.7 | 1.9 | 15 |
| 0.8 | lt | set_3 | 0.00 | 0.44 | 1134.1 | 22.2 | 1.3 | 15 |
| 0.8 | lh | set_1 | 0.00 | 0.24 | 1127.2 | 15.2 | 4.4 | 14 |
| 0.8 | lh | set_2 | 0.00 | 0.2 | 1125.3 | 13.4 | 1.1 | 13 |
| 0.8 | lh | set_3 | 0.00 | 0.16 | 1127.4 | 15.5 | 3.8 | 10 |
| 0.8 | qp | set_1 | 0.00 | 0.12 | 1128.7 | 16.7 | 2.1 | 6 |
| 0.8 | qp | set_2 | 0.00 | 0.16 | 1133.5 | 21.6 | 1.8 | 7 |
| 0.8 | qp | set_3 | 0.00 | 0.12 | 1128.1 | 16.2 | 2.8 | 5 |
| 0.8 | qt | set_1 | 0.00 | 0.36 | 1131.8 | 19.8 | 4.4 | 18 |
| 0.8 | qt | set_2 | 0.00 | 0.4 | 1126.9 | 14.9 | 5.1 | 17 |
| 0.8 | qt | set_3 | 0.00 | 0.48 | 1130.9 | 19.0 | 6.8 | 15 |
| 0.8 | qh | set_1 | 0.00 | 0.24 | 1116.8 | 4.9 | 7.8 | 11 |
| 0.8 | qh | set_2 | 0.00 | 0.16 | 1112.6 | 0.7 | 6.4 | 9 |
| 0.8 | qh | set_3 | 0.00 | 0.2 | 1119.9 | 8.0 | 1.6 | 7 |
| 0.8 | pt | set_1 | 0.00 | 0.4 | 1141.2 | 29.2 | 4.0 | 20 |
| 0.8 | pt | set_2 | 0.00 | 0.4 | 1136.7 | 24.8 | 3.8 | 19 |
| 0.8 | pt | set_3 | 0.00 | 0.44 | 1131.8 | 19.9 | 4.3 | 15 |
| 0.8 | ph | set_1 | 0.00 | 0.24 | 1127.6 | 15.6 | 3.6 | 14 |
| 0.8 | ph | set_2 | 0.00 | 0.2 | 1121.4 | 9.5 | 8.0 | 12 |
| 0.8 | ph | set_3 | 0.00 | 0.16 | 1128.0 | 16.1 | 2.9 | 10 |
| 0.8 | th | set_1 | 0.00 | 0.32 | 1149.9 | 38.0 | 5.2 | 21 |
| 0.8 | th | set_2 | 0.00 | 0.24 | 1129.6 | 17.7 | 1.3 | 17 |
| 0.8 | th | set_3 | 0.00 | 0.32 | 1135.8 | 23.8 | 6.0 | 15 |
| 0.8 | lqp | set_1 | 0.00 | 0.2 | 1128.4 | 16.5 | 2.3 | 6 |
| 0.8 | lqp | set_2 | 0.00 | 0.2 | 1130.3 | 18.4 | 9.3 | 6 |
| 0.8 | lqp | set_3 | 0.00 | 0.12 | 1127.6 | 15.6 | 3.7 | 5 |
| 0.8 | lqt | set_1 | 0.00 | 0.4 | 1138.1 | 26.2 | 1.9 | 18 |
| 0.8 | lqt | set_2 | 0.00 | 0.32 | 1128.6 | 16.7 | 2.1 | 16 |
| 0.8 | lqt | set_3 | 0.00 | 0.48 | 1138.5 | 26.6 | 1.5 | 15 |
| 0.8 | lqh | set_1 | 0.00 | 0.24 | 1124.6 | 12.6 | 1.6 | 13 |
| 0.8 | lqh | set_2 | 0.00 | 0.16 | 1116.6 | 4.6 | 9.1 | 10 |
| 0.8 | lqh | set_3 | 0.00 | 0.2 | 1129.9 | 17.9 | 1.1 | 10 |
| 0.8 | lpt | set_1 | 0.00 | 0.4 | 1135.6 | 23.7 | 6.6 | 19 |
| 0.8 | lpt | set_2 | 0.00 | 0.4 | 1126.4 | 14.5 | 6.6 | 17 |
| 0.8 | lpt | set_3 | 0.00 | 0.44 | 1132.0 | 20.1 | 4.0 | 15 |
| 0.8 | lph | set_1 | 0.00 | 0.24 | 1127.6 | 15.6 | 3.7 | 14 |
| 0.8 | lph | set_2 | 0.00 | 0.2 | 1121.5 | 9.6 | 7.7 | 12 |
| 0.8 | lph | set_3 | 0.00 | 0.16 | 1124.8 | 12.9 | 1.4 | 9 |
| 0.8 | qpt | set_1 | 0.00 | 0.44 | 1138.3 | 26.4 | 1.7 | 19 |
| 0.8 | qpt | set_2 | 0.00 | 0.4 | 1133.8 | 21.9 | 1.6 | 18 |
| 0.8 | qpt | set_3 | 0.00 | 0.44 | 1134.0 | 22.1 | 1.5 | 15 |
| 0.8 | qph | set_1 | 0.00 | 0.24 | 1117.7 | 5.8 | 5.2 | 11 |
| 0.8 | qph | set_2 | 0.00 | 0.2 | 1119.0 | 7.0 | 2.7 | 11 |
| 0.8 | qph | set_3 | 0.00 | 0.2 | 1126.3 | 14.4 | 7.1 | 9 |
| 0.8 | qth | set_1 | 0.00 | 0.32 | 1124.8 | 12.9 | 1.4 | 16 |
| 0.8 | qth | set_2 | 0.00 | 0.24 | 1117.9 | 5.9 | 4.8 | 14 |
| 0.8 | qth | set_3 | 0.00 | 0.48 | 1121.6 | 9.6 | 7.6 | 11 |
| 0.8 | pth | set_1 | 0.00 | 0.28 | 1128.5 | 16.5 | 2.4 | 17 |
| 0.8 | pth | set_2 | 0.00 | 0.24 | 1125.6 | 13.6 | 1.0 | 16 |
| 0.8 | pth | set_3 | 0.00 | 0.32 | 1124.5 | 12.5 | 1.7 | 12 |
| 0.8 | lqpt | set_1 | 0.00 | 0.44 | 1138.3 | 26.4 | 1.7 | 19 |
| 0.8 | lqpt | set_2 | 0.00 | 0.4 | 1133.8 | 21.9 | 1.6 | 18 |
| 0.8 | lqpt | set_3 | 0.00 | 0.44 | 1130.0 | 18.0 | 1.1 | 14 |
| 0.8 | lqph | set_1 | 0.00 | 0.24 | 1121.0 | 9.1 | 1.0 | 12 |
| 0.8 | lqph | set_2 | 0.00 | 0.2 | 1119.2 | 7.3 | 2.5 | 11 |
| 0.8 | lqph | set_3 | 0.00 | 0.2 | 1126.3 | 14.3 | 7.3 | 9 |
| 0.8 | lqth | set_1 | 0.00 | 0.28 | 1134.4 | 22.5 | 1.2 | 18 |
| 0.8 | lqth | set_2 | 0.00 | 0.24 | 1131.1 | 19.2 | 6.4 | 17 |
| 0.8 | lqth | set_3 | 0.00 | 0.4 | 1133.7 | 21.8 | 1.7 | 14 |
| 0.8 | lpth | set_1 | 0.00 | 0.28 | 1128.5 | 16.5 | 2.4 | 17 |
| 0.8 | lpth | set_2 | 0.00 | 0.24 | 1125.6 | 13.6 | 1.0 | 16 |
| 0.8 | lpth | set_3 | 0.00 | 0.32 | 1124.5 | 12.5 | 1.8 | 12 |
| 0.8 | lqpth | set_1 | 0.00 | 0.28 | 1128.7 | 16.8 | 2.1 | 17 |
| 0.8 | lqpth | set_2 | 0.00 | 0.24 | 1121.2 | 9.3 | 9.1 | 15 |
| 0.8 | lqpth | set_3 | 0.00 | 0.44 | 1121.4 | 9.5 | 8.2 | 11 |
| 0.9 | l | set_1 | 0.00 | 0.24 | 1174.1 | 62.2 | 2.9 | 5 |
| 0.9 | l | set_2 | 0.00 | 0.2 | 1176.7 | 64.8 | 8.2 | 3 |
| 0.9 | l | set_3 | 0.00 | 0.2 | 1181.7 | 69.7 | 6.9 | 3 |
| 0.9 | q | set_1 | 0.00 | 0.32 | 1170.0 | 58.0 | 2.3 | 5 |
| 0.9 | q | set_2 | 0.00 | 0.2 | 1169.9 | 58.0 | 2.4 | 4 |
| 0.9 | q | set_3 | 0.00 | 0.28 | 1186.2 | 74.3 | 7.0 | 3 |
| 0.9 | p | set_1 | 0.00 | 0.24 | 1146.8 | 34.9 | 2.5 | 4 |
| 0.9 | p | set_2 | 0.00 | 0.2 | 1166.4 | 54.4 | 1.4 | 4 |
| 0.9 | p | set_3 | 0.00 | 0.12 | 1184.2 | 72.2 | 1.9 | 3 |
| 0.9 | t | set_1 | 0.00 | 0.4 | 1149.5 | 37.6 | 6.5 | 21 |
| 0.9 | t | set_2 | 0.00 | 0.32 | 1149.5 | 37.6 | 6.5 | 21 |
| 0.9 | t | set_3 | 0.00 | 0.4 | 1154.8 | 42.9 | 4.6 | 20 |
| 0.9 | h | set_1 | 0.00 | 0.24 | 1134.4 | 22.5 | 1.2 | 15 |
| 0.9 | h | set_2 | 0.00 | 0.2 | 1131.4 | 19.5 | 5.7 | 14 |
| 0.9 | h | set_3 | 0.00 | 0.16 | 1132.4 | 20.5 | 3.4 | 11 |
| 0.9 | lq | set_1 | 0.00 | 0.12 | 1129.6 | 17.7 | 1.4 | 6 |
| 0.9 | lq | set_2 | 0.00 | 0.12 | 1129.0 | 17.1 | 1.8 | 5 |
| 0.9 | lq | set_3 | 0.00 | 0.12 | 1126.8 | 14.9 | 5.7 | 4 |
| 0.9 | lp | set_1 | 0.00 | 0.24 | 1143.9 | 32.0 | 1.0 | 4 |
| 0.9 | lp | set_2 | 0.00 | 0.24 | 1168.4 | 56.5 | 5.2 | 5 |
| 0.9 | lp | set_3 | 0.00 | 0.2 | 1172.9 | 61.0 | 5.6 | 4 |
| 0.9 | lt | set_1 | 0.00 | 0.44 | 1125.2 | 13.3 | 1.2 | 15 |
| 0.9 | lt | set_2 | 0.00 | 0.36 | 1115.4 | 3.5 | 1.7 | 13 |
| 0.9 | lt | set_3 | 0.00 | 0.44 | 1133.8 | 21.9 | 1.7 | 14 |
| 0.9 | lh | set_1 | 0.00 | 0.24 | 1129.0 | 17.1 | 1.8 | 14 |
| 0.9 | lh | set_2 | 0.00 | 0.2 | 1124.0 | 12.1 | 2.3 | 12 |
| 0.9 | lh | set_3 | 0.00 | 0.16 | 1135.1 | 23.1 | 9.2 | 12 |
| 0.9 | qp | set_1 | 0.00 | 0.16 | 1129.2 | 17.2 | 1.7 | 6 |
| 0.9 | qp | set_2 | 0.00 | 0.16 | 1131.2 | 19.3 | 6.4 | 6 |
| 0.9 | qp | set_3 | 0.00 | 0.12 | 1128.5 | 16.5 | 2.5 | 5 |
| 0.9 | qt | set_1 | 0.00 | 0.32 | 1127.1 | 15.2 | 4.8 | 16 |
| 0.9 | qt | set_2 | 0.00 | 0.44 | 1127.2 | 15.2 | 4.8 | 16 |
| 0.9 | qt | set_3 | 0.00 | 0.44 | 1130.3 | 18.3 | 1.0 | 14 |
| 0.9 | qh | set_1 | 0.00 | 0.24 | 1118.6 | 6.7 | 3.4 | 11 |
| 0.9 | qh | set_2 | 0.00 | 0.16 | 1111.9 | 0.0 | 9.8 | 8 |
| 0.9 | qh | set_3 | 0.00 | 0.2 | 1123.6 | 11.7 | 2.8 | 8 |
| 0.9 | pt | set_1 | 0.00 | 0.36 | 1136.4 | 24.4 | 4.8 | 18 |
| 0.9 | pt | set_2 | 0.00 | 0.36 | 1137.4 | 25.4 | 2.9 | 18 |
| 0.9 | pt | set_3 | 0.00 | 0.44 | 1124.8 | 12.9 | 1.5 | 12 |
| 0.9 | ph | set_1 | 0.00 | 0.24 | 1126.3 | 14.4 | 7.3 | 13 |
| 0.9 | ph | set_2 | 0.00 | 0.2 | 1123.6 | 11.6 | 2.9 | 12 |
| 0.9 | ph | set_3 | 0.00 | 0.16 | 1125.8 | 13.8 | 9.8 | 9 |
| 0.9 | th | set_1 | 0.00 | 0.24 | 1147.5 | 35.6 | 1.8 | 20 |
| 0.9 | th | set_2 | 0.00 | 0.24 | 1137.8 | 25.9 | 2.3 | 18 |
| 0.9 | th | set_3 | 0.00 | 0.28 | 1134.0 | 22.1 | 1.6 | 14 |
| 0.9 | lqp | set_1 | 0.00 | 0.2 | 1128.9 | 17.0 | 2.0 | 6 |
| 0.9 | lqp | set_2 | 0.00 | 0.2 | 1130.7 | 18.8 | 8.2 | 6 |
| 0.9 | lqp | set_3 | 0.00 | 0.16 | 1130.6 | 18.6 | 9.0 | 6 |
| 0.9 | lqt | set_1 | 0.00 | 0.36 | 1126.8 | 14.8 | 6.0 | 14 |
| 0.9 | lqt | set_2 | 0.00 | 0.32 | 1118.6 | 6.6 | 3.6 | 12 |
| 0.9 | lqt | set_3 | 0.00 | 0.36 | 1131.8 | 19.9 | 4.7 | 12 |
| 0.9 | lqh | set_1 | 0.00 | 0.24 | 1127.0 | 15.0 | 5.4 | 13 |
| 0.9 | lqh | set_2 | 0.00 | 0.16 | 1122.4 | 10.5 | 5.3 | 11 |
| 0.9 | lqh | set_3 | 0.00 | 0.2 | 1130.9 | 19.0 | 7.5 | 10 |
| 0.9 | lpt | set_1 | 0.00 | 0.36 | 1131.2 | 19.3 | 6.5 | 17 |
| 0.9 | lpt | set_2 | 0.00 | 0.36 | 1127.6 | 15.7 | 3.9 | 16 |
| 0.9 | lpt | set_3 | 0.00 | 0.44 | 1129.2 | 17.3 | 1.7 | 13 |
| 0.9 | lph | set_1 | 0.00 | 0.24 | 1134.5 | 22.6 | 1.2 | 15 |
| 0.9 | lph | set_2 | 0.00 | 0.2 | 1120.3 | 8.4 | 1.5 | 11 |
| 0.9 | lph | set_3 | 0.00 | 0.16 | 1125.7 | 13.8 | 1.0 | 9 |
| 0.9 | qpt | set_1 | 0.00 | 0.4 | 1133.9 | 21.9 | 1.7 | 17 |
| 0.9 | qpt | set_2 | 0.00 | 0.36 | 1129.8 | 17.9 | 1.3 | 16 |
| 0.9 | qpt | set_3 | 0.00 | 0.44 | 1134.3 | 22.3 | 1.4 | 14 |
| 0.9 | qph | set_1 | 0.00 | 0.24 | 1120.0 | 8.1 | 1.7 | 11 |
| 0.9 | qph | set_2 | 0.00 | 0.2 | 1115.1 | 3.2 | 2.0 | 9 |
| 0.9 | qph | set_3 | 0.00 | 0.2 | 1133.7 | 21.8 | 1.9 | 11 |
| 0.9 | qth | set_1 | 0.00 | 0.24 | 1133.0 | 21.1 | 2.7 | 17 |
| 0.9 | qth | set_2 | 0.00 | 0.2 | 1134.5 | 22.5 | 1.3 | 17 |
| 0.9 | qth | set_3 | 0.00 | 0.28 | 1124.6 | 12.6 | 1.8 | 11 |
| 0.9 | pth | set_1 | 0.00 | 0.24 | 1127.4 | 15.5 | 4.3 | 16 |
| 0.9 | pth | set_2 | 0.00 | 0.24 | 1124.4 | 12.5 | 2.0 | 15 |
| 0.9 | pth | set_3 | 0.00 | 0.32 | 1123.2 | 11.3 | 3.5 | 11 |
| 0.9 | lqpt | set_1 | 0.00 | 0.4 | 1129.3 | 17.4 | 1.7 | 16 |
| 0.9 | lqpt | set_2 | 0.00 | 0.36 | 1125.4 | 13.5 | 1.2 | 15 |
| 0.9 | lqpt | set_3 | 0.00 | 0.44 | 1134.3 | 22.3 | 1.4 | 14 |
| 0.9 | lqph | set_1 | 0.00 | 0.24 | 1120.0 | 8.1 | 1.8 | 11 |
| 0.9 | lqph | set_2 | 0.00 | 0.2 | 1114.8 | 2.9 | 2.4 | 9 |
| 0.9 | lqph | set_3 | 0.00 | 0.2 | 1121.4 | 9.5 | 9.0 | 7 |
| 0.9 | lqth | set_1 | 0.00 | 0.24 | 1156.0 | 44.1 | 2.7 | 21 |
| 0.9 | lqth | set_2 | 0.00 | 0.24 | 1135.6 | 23.7 | 7.4 | 17 |
| 0.9 | lqth | set_3 | 0.00 | 0.28 | 1133.1 | 21.1 | 2.6 | 13 |
| 0.9 | lpth | set_1 | 0.00 | 0.24 | 1127.4 | 15.5 | 4.4 | 16 |
| 0.9 | lpth | set_2 | 0.00 | 0.24 | 1124.4 | 12.5 | 2.0 | 15 |
| 0.9 | lpth | set_3 | 0.00 | 0.32 | 1123.2 | 11.3 | 3.6 | 11 |
| 0.9 | lqpth | set_1 | 0.00 | 0.28 | 1137.8 | 25.9 | 2.4 | 18 |
| 0.9 | lqpth | set_2 | 0.00 | 0.24 | 1125.0 | 13.0 | 1.5 | 15 |
| 0.9 | lqpth | set_3 | 0.00 | 0.32 | 1124.1 | 12.1 | 2.4 | 11 |
| 1 | l | set_1 | 0.00 | 0.24 | 1174.3 | 62.3 | 3.0 | 5 |
| 1 | l | set_2 | 0.00 | 0.2 | 1176.7 | 64.8 | 8.9 | 3 |
| 1 | l | set_3 | 0.00 | 0.16 | 1181.7 | 69.7 | 7.5 | 3 |
| 1 | q | set_1 | 0.00 | 0.32 | 1170.1 | 58.1 | 2.4 | 5 |
| 1 | q | set_2 | 0.00 | 0.2 | 1170.1 | 58.1 | 2.5 | 4 |
| 1 | q | set_3 | 0.00 | 0.28 | 1186.3 | 74.3 | 7.5 | 3 |
| 1 | p | set_1 | 0.00 | 0.24 | 1147.1 | 35.2 | 2.3 | 4 |
| 1 | p | set_2 | 0.00 | 0.2 | 1168.1 | 56.2 | 6.5 | 4 |
| 1 | p | set_3 | 0.00 | 0.12 | 1185.9 | 73.9 | 9.2 | 3 |
| 1 | t | set_1 | 0.00 | 0.4 | 1147.4 | 35.5 | 2.0 | 20 |
| 1 | t | set_2 | 0.00 | 0.32 | 1147.4 | 35.5 | 2.0 | 20 |
| 1 | t | set_3 | 0.00 | 0.4 | 1146.9 | 35.0 | 2.6 | 18 |
| 1 | h | set_1 | 0.00 | 0.24 | 1125.4 | 13.5 | 1.2 | 12 |
| 1 | h | set_2 | 0.00 | 0.2 | 1122.8 | 10.9 | 4.5 | 11 |
| 1 | h | set_3 | 0.00 | 0.12 | 1130.1 | 18.2 | 1.1 | 10 |
| 1 | lq | set_1 | 0.00 | 0.12 | 1129.7 | 17.8 | 1.4 | 6 |
| 1 | lq | set_2 | 0.00 | 0.12 | 1129.1 | 17.2 | 1.9 | 5 |
| 1 | lq | set_3 | 0.00 | 0.12 | 1126.9 | 14.9 | 6.0 | 4 |
| 1 | lp | set_1 | 0.00 | 0.24 | 1144.3 | 32.4 | 9.8 | 4 |
| 1 | lp | set_2 | 0.00 | 0.2 | 1170.2 | 58.3 | 2.3 | 5 |
| 1 | lp | set_3 | 0.00 | 0.2 | 1177.9 | 65.9 | 5.1 | 5 |
| 1 | lt | set_1 | 0.00 | 0.4 | 1122.2 | 10.2 | 6.4 | 13 |
| 1 | lt | set_2 | 0.00 | 0.4 | 1123.6 | 11.7 | 3.0 | 14 |
| 1 | lt | set_3 | 0.00 | 0.36 | 1126.6 | 14.7 | 6.9 | 11 |
| 1 | lh | set_1 | 0.00 | 0.24 | 1135.8 | 23.9 | 6.8 | 15 |
| 1 | lh | set_2 | 0.00 | 0.2 | 1128.8 | 16.9 | 2.2 | 13 |
| 1 | lh | set_3 | 0.00 | 0.12 | 1136.1 | 24.1 | 6.1 | 12 |
| 1 | qp | set_1 | 0.00 | 0.16 | 1129.7 | 17.8 | 1.4 | 6 |
| 1 | qp | set_2 | 0.00 | 0.16 | 1131.6 | 19.7 | 5.7 | 6 |
| 1 | qp | set_3 | 0.00 | 0.12 | 1128.8 | 16.9 | 2.2 | 5 |
| 1 | qt | set_1 | 0.00 | 0.32 | 1126.9 | 15.0 | 6.0 | 15 |
| 1 | qt | set_2 | 0.00 | 0.44 | 1122.7 | 10.8 | 4.9 | 14 |
| 1 | qt | set_3 | 0.00 | 0.44 | 1129.4 | 17.4 | 1.7 | 13 |
| 1 | qh | set_1 | 0.00 | 0.24 | 1120.9 | 8.9 | 1.2 | 11 |
| 1 | qh | set_2 | 0.00 | 0.16 | 1116.1 | 4.1 | 1.3 | 9 |
| 1 | qh | set_3 | 0.00 | 0.2 | 1121.6 | 9.7 | 8.6 | 7 |
| 1 | pt | set_1 | 0.00 | 0.36 | 1132.1 | 20.2 | 4.4 | 16 |
| 1 | pt | set_2 | 0.00 | 0.36 | 1129.1 | 17.2 | 2.0 | 15 |
| 1 | pt | set_3 | 0.00 | 0.4 | 1120.5 | 8.5 | 1.5 | 10 |
| 1 | ph | set_1 | 0.00 | 0.24 | 1125.2 | 13.3 | 1.4 | 12 |
| 1 | ph | set_2 | 0.00 | 0.2 | 1122.6 | 10.7 | 5.1 | 11 |
| 1 | ph | set_3 | 0.00 | 0.12 | 1133.2 | 21.3 | 2.6 | 11 |
| 1 | th | set_1 | 0.00 | 0.24 | 1135.8 | 23.9 | 7.1 | 17 |
| 1 | th | set_2 | 0.00 | 0.2 | 1131.7 | 19.7 | 5.6 | 16 |
| 1 | th | set_3 | 0.00 | 0.2 | 1128.8 | 16.9 | 2.3 | 12 |
| 1 | lqp | set_1 | 0.00 | 0.2 | 1132.1 | 20.2 | 4.5 | 7 |
| 1 | lqp | set_2 | 0.00 | 0.2 | 1131.1 | 19.2 | 7.3 | 6 |
| 1 | lqp | set_3 | 0.00 | 0.2 | 1131.0 | 19.1 | 7.9 | 6 |
| 1 | lqt | set_1 | 0.00 | 0.36 | 1128.0 | 16.1 | 3.4 | 13 |
| 1 | lqt | set_2 | 0.00 | 0.32 | 1123.5 | 11.6 | 3.3 | 12 |
| 1 | lqt | set_3 | 0.00 | 0.28 | 1132.1 | 20.1 | 4.6 | 11 |
| 1 | lqh | set_1 | 0.00 | 0.24 | 1129.9 | 18.0 | 1.3 | 13 |
| 1 | lqh | set_2 | 0.00 | 0.16 | 1123.8 | 11.9 | 2.8 | 11 |
| 1 | lqh | set_3 | 0.00 | 0.2 | 1125.7 | 13.8 | 1.0 | 8 |
| 1 | lpt | set_1 | 0.00 | 0.36 | 1131.7 | 19.8 | 5.4 | 16 |
| 1 | lpt | set_2 | 0.00 | 0.36 | 1124.0 | 12.1 | 2.6 | 14 |
| 1 | lpt | set_3 | 0.00 | 0.4 | 1125.4 | 13.4 | 1.3 | 11 |
| 1 | lph | set_1 | 0.00 | 0.24 | 1125.2 | 13.3 | 1.4 | 12 |
| 1 | lph | set_2 | 0.00 | 0.2 | 1126.2 | 14.3 | 8.6 | 12 |
| 1 | lph | set_3 | 0.00 | 0.12 | 1133.2 | 21.2 | 2.7 | 11 |
| 1 | qpt | set_1 | 0.00 | 0.36 | 1130.1 | 18.2 | 1.2 | 15 |
| 1 | qpt | set_2 | 0.00 | 0.36 | 1130.1 | 18.2 | 1.2 | 15 |
| 1 | qpt | set_3 | 0.00 | 0.36 | 1129.5 | 17.6 | 1.6 | 12 |
| 1 | qph | set_1 | 0.00 | 0.24 | 1122.5 | 10.5 | 5.7 | 11 |
| 1 | qph | set_2 | 0.00 | 0.2 | 1114.3 | 2.4 | 3.4 | 8 |
| 1 | qph | set_3 | 0.00 | 0.2 | 1122.3 | 10.4 | 6.0 | 7 |
| 1 | qth | set_1 | 0.00 | 0.2 | 1132.4 | 20.5 | 4.0 | 16 |
| 1 | qth | set_2 | 0.00 | 0.2 | 1125.0 | 13.1 | 1.6 | 14 |
| 1 | qth | set_3 | 0.00 | 0.24 | 1123.7 | 11.8 | 3.0 | 10 |
| 1 | pth | set_1 | 0.00 | 0.24 | 1131.3 | 19.4 | 6.9 | 16 |
| 1 | pth | set_2 | 0.00 | 0.24 | 1127.5 | 15.6 | 4.5 | 15 |
| 1 | pth | set_3 | 0.00 | 0.2 | 1125.4 | 13.4 | 1.3 | 11 |
| 1 | lqpt | set_1 | 0.00 | 0.36 | 1129.9 | 18.0 | 1.4 | 15 |
| 1 | lqpt | set_2 | 0.00 | 0.36 | 1125.7 | 13.8 | 1.1 | 14 |
| 1 | lqpt | set_3 | 0.00 | 0.36 | 1129.8 | 17.8 | 1.5 | 12 |
| 1 | lqph | set_1 | 0.00 | 0.24 | 1122.5 | 10.5 | 5.8 | 11 |
| 1 | lqph | set_2 | 0.00 | 0.2 | 1114.3 | 2.4 | 3.4 | 8 |
| 1 | lqph | set_3 | 0.00 | 0.2 | 1125.2 | 13.3 | 1.4 | 8 |
| 1 | lqth | set_1 | 0.00 | 0.24 | 1143.5 | 31.6 | 1.5 | 18 |
| 1 | lqth | set_2 | 0.00 | 0.2 | 1139.3 | 27.3 | 1.3 | 17 |
| 1 | lqth | set_3 | 0.00 | 0.24 | 1136.0 | 24.1 | 6.5 | 13 |
| 1 | lpth | set_1 | 0.00 | 0.24 | 1131.3 | 19.4 | 7.0 | 16 |
| 1 | lpth | set_2 | 0.00 | 0.24 | 1127.5 | 15.6 | 4.6 | 15 |
| 1 | lpth | set_3 | 0.00 | 0.2 | 1125.4 | 13.4 | 1.3 | 11 |
| 1 | lqpth | set_1 | 0.00 | 0.28 | 1132.2 | 20.3 | 4.4 | 16 |
| 1 | lqpth | set_2 | 0.00 | 0.24 | 1124.4 | 12.5 | 2.1 | 14 |
| 1 | lqpth | set_3 | 0.00 | 0.24 | 1126.4 | 14.4 | 8.3 | 11 |
| 1.25 | l | set_1 | 0.00 | 0.24 | 1174.6 | 62.7 | 2.7 | 5 |
| 1.25 | l | set_2 | 0.00 | 0.2 | 1176.8 | 64.8 | 9.5 | 3 |
| 1.25 | l | set_3 | 0.00 | 0.16 | 1181.7 | 69.8 | 8.1 | 3 |
| 1.25 | q | set_1 | 0.00 | 0.4 | 1170.4 | 58.4 | 2.3 | 5 |
| 1.25 | q | set_2 | 0.00 | 0.24 | 1170.4 | 58.4 | 2.3 | 4 |
| 1.25 | q | set_3 | 0.00 | 0.28 | 1186.3 | 74.4 | 7.9 | 3 |
| 1.25 | p | set_1 | 0.00 | 0.24 | 1148.1 | 36.1 | 1.6 | 4 |
| 1.25 | p | set_2 | 0.00 | 0.2 | 1172.9 | 61.0 | 6.6 | 4 |
| 1.25 | p | set_3 | 0.00 | 0.12 | 1185.8 | 73.9 | 1.0 | 2 |
| 1.25 | t | set_1 | 0.00 | 0.28 | 1135.2 | 23.3 | 1.0 | 16 |
| 1.25 | t | set_2 | 0.00 | 0.32 | 1135.2 | 23.3 | 1.0 | 16 |
| 1.25 | t | set_3 | 0.00 | 0.28 | 1148.6 | 36.7 | 1.2 | 17 |
| 1.25 | h | set_1 | 0.00 | 0.24 | 1137.2 | 25.2 | 3.8 | 14 |
| 1.25 | h | set_2 | 0.00 | 0.2 | 1133.3 | 21.3 | 2.7 | 13 |
| 1.25 | h | set_3 | 0.00 | 0.12 | 1129.6 | 17.7 | 1.6 | 9 |
| 1.25 | lq | set_1 | 0.00 | 0.12 | 1130.0 | 18.1 | 1.3 | 6 |
| 1.25 | lq | set_2 | 0.00 | 0.12 | 1129.4 | 17.5 | 1.8 | 5 |
| 1.25 | lq | set_3 | 0.00 | 0.12 | 1127.1 | 15.2 | 5.7 | 4 |
| 1.25 | lp | set_1 | 0.00 | 0.24 | 1145.3 | 33.4 | 6.6 | 4 |
| 1.25 | lp | set_2 | 0.00 | 0.2 | 1175.1 | 63.2 | 2.2 | 5 |
| 1.25 | lp | set_3 | 0.00 | 0.2 | 1182.8 | 70.9 | 4.8 | 5 |
| 1.25 | lt | set_1 | 0.00 | 0.36 | 1126.2 | 14.3 | 9.1 | 11 |
| 1.25 | lt | set_2 | 0.00 | 0.36 | 1124.2 | 12.2 | 2.5 | 11 |
| 1.25 | lt | set_3 | 0.00 | 0.36 | 1123.3 | 11.4 | 3.8 | 8 |
| 1.25 | lh | set_1 | 0.00 | 0.24 | 1139.6 | 27.7 | 1.1 | 15 |
| 1.25 | lh | set_2 | 0.00 | 0.12 | 1131.8 | 19.8 | 5.7 | 13 |
| 1.25 | lh | set_3 | 0.00 | 0.12 | 1131.5 | 19.6 | 6.6 | 10 |
| 1.25 | qp | set_1 | 0.00 | 0.16 | 1131.2 | 19.3 | 7.6 | 6 |
| 1.25 | qp | set_2 | 0.00 | 0.16 | 1132.7 | 20.8 | 3.5 | 6 |
| 1.25 | qp | set_3 | 0.00 | 0.16 | 1129.9 | 18.0 | 1.4 | 5 |
| 1.25 | qt | set_1 | 0.00 | 0.36 | 1129.2 | 17.2 | 2.1 | 13 |
| 1.25 | qt | set_2 | 0.00 | 0.32 | 1125.3 | 13.3 | 1.4 | 12 |
| 1.25 | qt | set_3 | 0.00 | 0.32 | 1130.1 | 18.2 | 1.3 | 11 |
| 1.25 | qh | set_1 | 0.00 | 0.24 | 1118.7 | 6.8 | 3.9 | 9 |
| 1.25 | qh | set_2 | 0.00 | 0.16 | 1115.9 | 4.0 | 1.6 | 8 |
| 1.25 | qh | set_3 | 0.00 | 0.16 | 1123.7 | 11.8 | 3.3 | 7 |
| 1.25 | pt | set_1 | 0.00 | 0.36 | 1135.5 | 23.6 | 8.9 | 14 |
| 1.25 | pt | set_2 | 0.00 | 0.36 | 1131.2 | 19.3 | 7.7 | 13 |
| 1.25 | pt | set_3 | 0.00 | 0.36 | 1128.4 | 16.4 | 3.2 | 11 |
| 1.25 | ph | set_1 | 0.00 | 0.24 | 1136.8 | 24.9 | 4.6 | 14 |
| 1.25 | ph | set_2 | 0.00 | 0.2 | 1136.9 | 25.0 | 4.4 | 14 |
| 1.25 | ph | set_3 | 0.00 | 0.12 | 1132.3 | 20.4 | 4.4 | 10 |
| 1.25 | th | set_1 | 0.00 | 0.24 | 1122.9 | 11.0 | 5.0 | 12 |
| 1.25 | th | set_2 | 0.00 | 0.2 | 1122.9 | 11.0 | 5.0 | 12 |
| 1.25 | th | set_3 | 0.00 | 0.12 | 1122.9 | 11.0 | 5.0 | 9 |
| 1.25 | lqp | set_1 | 0.00 | 0.2 | 1131.0 | 19.1 | 8.6 | 6 |
| 1.25 | lqp | set_2 | 0.00 | 0.2 | 1132.2 | 20.3 | 4.8 | 6 |
| 1.25 | lqp | set_3 | 0.00 | 0.16 | 1132.2 | 20.2 | 4.8 | 6 |
| 1.25 | lqt | set_1 | 0.00 | 0.2 | 1131.0 | 19.1 | 8.6 | 11 |
| 1.25 | lqt | set_2 | 0.00 | 0.2 | 1125.8 | 13.8 | 1.1 | 10 |
| 1.25 | lqt | set_3 | 0.00 | 0.2 | 1128.2 | 16.2 | 3.6 | 8 |
| 1.25 | lqh | set_1 | 0.00 | 0.24 | 1127.2 | 15.2 | 5.9 | 11 |
| 1.25 | lqh | set_2 | 0.00 | 0.16 | 1120.8 | 8.9 | 1.4 | 9 |
| 1.25 | lqh | set_3 | 0.00 | 0.16 | 1125.3 | 13.4 | 1.4 | 7 |
| 1.25 | lpt | set_1 | 0.00 | 0.36 | 1131.6 | 19.6 | 6.6 | 13 |
| 1.25 | lpt | set_2 | 0.00 | 0.36 | 1127.3 | 15.4 | 5.6 | 12 |
| 1.25 | lpt | set_3 | 0.00 | 0.36 | 1132.6 | 20.7 | 3.9 | 11 |
| 1.25 | lph | set_1 | 0.00 | 0.24 | 1145.4 | 33.4 | 6.6 | 16 |
| 1.25 | lph | set_2 | 0.00 | 0.2 | 1141.1 | 29.2 | 5.6 | 15 |
| 1.25 | lph | set_3 | 0.00 | 0.12 | 1132.3 | 20.4 | 4.5 | 10 |
| 1.25 | qpt | set_1 | 0.00 | 0.28 | 1125.0 | 13.1 | 1.7 | 11 |
| 1.25 | qpt | set_2 | 0.00 | 0.32 | 1131.1 | 19.2 | 8.2 | 13 |
| 1.25 | qpt | set_3 | 0.00 | 0.32 | 1133.1 | 21.2 | 3.1 | 11 |
| 1.25 | qph | set_1 | 0.00 | 0.24 | 1117.1 | 5.1 | 9.4 | 8 |
| 1.25 | qph | set_2 | 0.00 | 0.16 | 1120.2 | 8.3 | 1.9 | 9 |
| 1.25 | qph | set_3 | 0.00 | 0.16 | 1124.5 | 12.6 | 2.2 | 7 |
| 1.25 | qth | set_1 | 0.00 | 0.2 | 1113.5 | 1.6 | 5.5 | 9 |
| 1.25 | qth | set_2 | 0.00 | 0.16 | 1113.5 | 1.6 | 5.6 | 9 |
| 1.25 | qth | set_3 | 0.00 | 0.12 | 1118.1 | 6.1 | 5.7 | 7 |
| 1.25 | pth | set_1 | 0.00 | 0.24 | 1122.8 | 10.9 | 5.2 | 12 |
| 1.25 | pth | set_2 | 0.00 | 0.2 | 1122.9 | 10.9 | 5.2 | 12 |
| 1.25 | pth | set_3 | 0.00 | 0.12 | 1122.7 | 10.8 | 5.7 | 9 |
| 1.25 | lqpt | set_1 | 0.00 | 0.28 | 1128.2 | 16.3 | 3.6 | 12 |
| 1.25 | lqpt | set_2 | 0.00 | 0.32 | 1127.5 | 15.6 | 5.2 | 12 |
| 1.25 | lqpt | set_3 | 0.00 | 0.32 | 1133.3 | 21.4 | 2.8 | 11 |
| 1.25 | lqph | set_1 | 0.00 | 0.24 | 1117.1 | 5.1 | 9.5 | 8 |
| 1.25 | lqph | set_2 | 0.00 | 0.2 | 1123.4 | 11.5 | 4.0 | 10 |
| 1.25 | lqph | set_3 | 0.00 | 0.16 | 1124.5 | 12.6 | 2.3 | 7 |
| 1.25 | lqth | set_1 | 0.00 | 0.2 | 1123.0 | 11.1 | 4.8 | 11 |
| 1.25 | lqth | set_2 | 0.00 | 0.16 | 1126.7 | 14.8 | 7.6 | 12 |
| 1.25 | lqth | set_3 | 0.00 | 0.2 | 1130.1 | 18.1 | 1.4 | 10 |
| 1.25 | lpth | set_1 | 0.00 | 0.24 | 1122.8 | 10.9 | 5.3 | 12 |
| 1.25 | lpth | set_2 | 0.00 | 0.2 | 1122.9 | 10.9 | 5.2 | 12 |
| 1.25 | lpth | set_3 | 0.00 | 0.12 | 1122.7 | 10.8 | 5.8 | 9 |
| 1.25 | lqpth | set_1 | 0.00 | 0.24 | 1120.4 | 8.5 | 1.8 | 11 |
| 1.25 | lqpth | set_2 | 0.00 | 0.2 | 1120.3 | 8.3 | 1.9 | 11 |
| 1.25 | lqpth | set_3 | 0.00 | 0.16 | 1121.0 | 9.0 | 1.3 | 8 |
| 1.5 | l | set_1 | 0.00 | 0.24 | 1172.5 | 60.6 | 8.8 | 4 |
| 1.5 | l | set_2 | 0.00 | 0.2 | 1176.8 | 64.9 | 1.0 | 3 |
| 1.5 | l | set_3 | 0.00 | 0.16 | 1181.7 | 69.8 | 8.9 | 3 |
| 1.5 | q | set_1 | 0.00 | 0.4 | 1170.7 | 58.8 | 2.1 | 5 |
| 1.5 | q | set_2 | 0.00 | 0.24 | 1170.7 | 58.8 | 2.1 | 4 |
| 1.5 | q | set_3 | 0.00 | 0.28 | 1186.4 | 74.5 | 8.4 | 3 |
| 1.5 | p | set_1 | 0.00 | 0.24 | 1149.1 | 37.2 | 1.0 | 4 |
| 1.5 | p | set_2 | 0.00 | 0.2 | 1178.0 | 66.1 | 5.7 | 4 |
| 1.5 | p | set_3 | 0.00 | 0.12 | 1185.9 | 73.9 | 1.1 | 2 |
| 1.5 | t | set_1 | 0.00 | 0.28 | 1139.3 | 27.4 | 1.4 | 15 |
| 1.5 | t | set_2 | 0.00 | 0.28 | 1139.3 | 27.4 | 1.4 | 15 |
| 1.5 | t | set_3 | 0.00 | 0.28 | 1151.2 | 39.3 | 3.8 | 16 |
| 1.5 | h | set_1 | 0.00 | 0.24 | 1129.5 | 17.6 | 1.9 | 11 |
| 1.5 | h | set_2 | 0.00 | 0.12 | 1129.5 | 17.6 | 1.9 | 11 |
| 1.5 | h | set_3 | 0.00 | 0.12 | 1138.2 | 26.3 | 2.4 | 11 |
| 1.5 | lq | set_1 | 0.00 | 0.12 | 1130.3 | 18.4 | 1.2 | 6 |
| 1.5 | lq | set_2 | 0.00 | 0.12 | 1129.7 | 17.8 | 1.8 | 5 |
| 1.5 | lq | set_3 | 0.00 | 0.08 | 1127.4 | 15.5 | 5.5 | 4 |
| 1.5 | lp | set_1 | 0.00 | 0.24 | 1146.4 | 34.5 | 4.2 | 4 |
| 1.5 | lp | set_2 | 0.00 | 0.2 | 1177.1 | 65.1 | 9.2 | 4 |
| 1.5 | lp | set_3 | 0.00 | 0.2 | 1184.2 | 72.3 | 2.6 | 4 |
| 1.5 | lt | set_1 | 0.00 | 0.32 | 1127.3 | 15.4 | 6.0 | 9 |
| 1.5 | lt | set_2 | 0.00 | 0.32 | 1133.8 | 21.9 | 2.3 | 11 |
| 1.5 | lt | set_3 | 0.00 | 0.32 | 1127.7 | 15.8 | 4.8 | 8 |
| 1.5 | lh | set_1 | 0.00 | 0.24 | 1138.5 | 26.5 | 2.2 | 14 |
| 1.5 | lh | set_2 | 0.00 | 0.08 | 1134.8 | 22.8 | 1.4 | 13 |
| 1.5 | lh | set_3 | 0.00 | 0.12 | 1133.8 | 21.9 | 2.2 | 10 |
| 1.5 | qp | set_1 | 0.00 | 0.16 | 1135.6 | 23.7 | 9.3 | 7 |
| 1.5 | qp | set_2 | 0.00 | 0.16 | 1134.1 | 22.2 | 1.9 | 6 |
| 1.5 | qp | set_3 | 0.00 | 0.16 | 1131.1 | 19.1 | 9.2 | 5 |
| 1.5 | qt | set_1 | 0.00 | 0.36 | 1130.2 | 18.3 | 1.4 | 10 |
| 1.5 | qt | set_2 | 0.00 | 0.32 | 1133.6 | 21.7 | 2.5 | 11 |
| 1.5 | qt | set_3 | 0.00 | 0.32 | 1129.7 | 17.8 | 1.8 | 9 |
| 1.5 | qh | set_1 | 0.00 | 0.24 | 1124.8 | 12.9 | 2.0 | 10 |
| 1.5 | qh | set_2 | 0.00 | 0.12 | 1122.0 | 10.0 | 8.7 | 9 |
| 1.5 | qh | set_3 | 0.00 | 0.12 | 1123.1 | 11.2 | 4.9 | 6 |
| 1.5 | pt | set_1 | 0.00 | 0.36 | 1125.9 | 13.9 | 1.2 | 9 |
| 1.5 | pt | set_2 | 0.00 | 0.36 | 1123.2 | 11.3 | 4.7 | 8 |
| 1.5 | pt | set_3 | 0.00 | 0.32 | 1130.7 | 18.8 | 1.1 | 10 |
| 1.5 | ph | set_1 | 0.00 | 0.24 | 1136.1 | 24.2 | 7.5 | 13 |
| 1.5 | ph | set_2 | 0.00 | 0.12 | 1132.5 | 20.6 | 4.4 | 12 |
| 1.5 | ph | set_3 | 0.00 | 0.12 | 1141.8 | 29.9 | 4.3 | 12 |
| 1.5 | th | set_1 | 0.00 | 0.24 | 1133.7 | 21.8 | 2.5 | 14 |
| 1.5 | th | set_2 | 0.00 | 0.08 | 1133.7 | 21.8 | 2.5 | 14 |
| 1.5 | th | set_3 | 0.00 | 0.12 | 1135.6 | 23.7 | 9.7 | 12 |
| 1.5 | lqp | set_1 | 0.00 | 0.16 | 1135.5 | 23.6 | 1.0 | 7 |
| 1.5 | lqp | set_2 | 0.00 | 0.16 | 1133.6 | 21.6 | 2.6 | 6 |
| 1.5 | lqp | set_3 | 0.00 | 0.16 | 1130.9 | 19.0 | 1.0 | 5 |
| 1.5 | lqt | set_1 | 0.00 | 0.16 | 1124.7 | 12.7 | 2.3 | 7 |
| 1.5 | lqt | set_2 | 0.00 | 0.16 | 1127.6 | 15.7 | 5.3 | 8 |
| 1.5 | lqt | set_3 | 0.00 | 0.16 | 1125.8 | 13.9 | 1.2 | 6 |
| 1.5 | lqh | set_1 | 0.00 | 0.24 | 1133.6 | 21.6 | 2.7 | 12 |
| 1.5 | lqh | set_2 | 0.00 | 0.16 | 1123.7 | 11.8 | 3.7 | 9 |
| 1.5 | lqh | set_3 | 0.00 | 0.16 | 1124.7 | 12.7 | 2.3 | 6 |
| 1.5 | lpt | set_1 | 0.00 | 0.36 | 1129.5 | 17.6 | 2.0 | 10 |
| 1.5 | lpt | set_2 | 0.00 | 0.36 | 1126.5 | 14.5 | 9.4 | 9 |
| 1.5 | lpt | set_3 | 0.00 | 0.36 | 1130.8 | 18.8 | 1.1 | 9 |
| 1.5 | lph | set_1 | 0.00 | 0.24 | 1136.2 | 24.2 | 7.4 | 13 |
| 1.5 | lph | set_2 | 0.00 | 0.12 | 1132.5 | 20.6 | 4.7 | 12 |
| 1.5 | lph | set_3 | 0.00 | 0.12 | 1141.7 | 29.7 | 4.7 | 12 |
| 1.5 | qpt | set_1 | 0.00 | 0.2 | 1128.4 | 16.5 | 3.6 | 10 |
| 1.5 | qpt | set_2 | 0.00 | 0.2 | 1129.0 | 17.1 | 2.6 | 10 |
| 1.5 | qpt | set_3 | 0.00 | 0.2 | 1128.7 | 16.8 | 3.0 | 8 |
| 1.5 | qph | set_1 | 0.00 | 0.24 | 1122.7 | 10.8 | 6.1 | 9 |
| 1.5 | qph | set_2 | 0.00 | 0.16 | 1126.1 | 14.1 | 1.1 | 10 |
| 1.5 | qph | set_3 | 0.00 | 0.12 | 1129.7 | 17.7 | 1.9 | 8 |
| 1.5 | qth | set_1 | 0.00 | 0.24 | 1122.7 | 10.8 | 6.1 | 11 |
| 1.5 | qth | set_2 | 0.00 | 0.16 | 1119.5 | 7.5 | 3.1 | 10 |
| 1.5 | qth | set_3 | 0.00 | 0.08 | 1120.3 | 8.4 | 2.0 | 7 |
| 1.5 | pth | set_1 | 0.00 | 0.24 | 1125.8 | 13.9 | 1.3 | 12 |
| 1.5 | pth | set_2 | 0.00 | 0.08 | 1129.6 | 17.6 | 2.0 | 13 |
| 1.5 | pth | set_3 | 0.00 | 0.12 | 1122.3 | 10.4 | 7.8 | 8 |
| 1.5 | lqpt | set_1 | 0.00 | 0.2 | 1128.6 | 16.7 | 3.3 | 10 |
| 1.5 | lqpt | set_2 | 0.00 | 0.2 | 1125.8 | 13.8 | 1.3 | 9 |
| 1.5 | lqpt | set_3 | 0.00 | 0.2 | 1128.2 | 16.3 | 4.0 | 8 |
| 1.5 | lqph | set_1 | 0.00 | 0.24 | 1125.9 | 14.0 | 1.3 | 10 |
| 1.5 | lqph | set_2 | 0.00 | 0.16 | 1122.9 | 11.0 | 5.7 | 9 |
| 1.5 | lqph | set_3 | 0.00 | 0.12 | 1129.6 | 17.7 | 2.0 | 8 |
| 1.5 | lqth | set_1 | 0.00 | 0.24 | 1113.5 | 1.6 | 6.3 | 7 |
| 1.5 | lqth | set_2 | 0.00 | 0.16 | 1113.5 | 1.6 | 6.4 | 7 |
| 1.5 | lqth | set_3 | 0.00 | 0.12 | 1120.8 | 8.9 | 1.6 | 6 |
| 1.5 | lpth | set_1 | 0.00 | 0.24 | 1125.8 | 13.9 | 1.3 | 12 |
| 1.5 | lpth | set_2 | 0.00 | 0.08 | 1129.6 | 17.6 | 2.0 | 13 |
| 1.5 | lpth | set_3 | 0.00 | 0.12 | 1122.3 | 10.4 | 7.9 | 8 |
| 1.5 | lqpth | set_1 | 0.00 | 0.24 | 1123.6 | 11.6 | 4.2 | 11 |
| 1.5 | lqpth | set_2 | 0.00 | 0.16 | 1123.5 | 11.6 | 4.2 | 11 |
| 1.5 | lqpth | set_3 | 0.00 | 0.12 | 1123.9 | 12.0 | 3.6 | 8 |
| 1.75 | l | set_1 | 0.00 | 0.2 | 1172.7 | 60.7 | 9.2 | 4 |
| 1.75 | l | set_2 | 0.00 | 0.2 | 1176.8 | 64.9 | 1.1 | 3 |
| 1.75 | l | set_3 | 0.00 | 0.16 | 1181.7 | 69.8 | 9.9 | 3 |
| 1.75 | q | set_1 | 0.00 | 0.32 | 1171.1 | 59.2 | 1.9 | 5 |
| 1.75 | q | set_2 | 0.00 | 0.24 | 1171.1 | 59.2 | 1.9 | 4 |
| 1.75 | q | set_3 | 0.00 | 0.28 | 1186.5 | 74.6 | 9.0 | 3 |
| 1.75 | p | set_1 | 0.00 | 0.24 | 1150.2 | 38.3 | 6.9 | 4 |
| 1.75 | p | set_2 | 0.00 | 0.2 | 1175.9 | 63.9 | 1.8 | 3 |
| 1.75 | p | set_3 | 0.00 | 0.12 | 1185.9 | 74.0 | 1.2 | 2 |
| 1.75 | t | set_1 | 0.00 | 0.28 | 1143.9 | 32.0 | 1.6 | 14 |
| 1.75 | t | set_2 | 0.00 | 0.28 | 1143.9 | 32.0 | 1.6 | 14 |
| 1.75 | t | set_3 | 0.00 | 0.28 | 1150.7 | 38.8 | 5.4 | 14 |
| 1.75 | h | set_1 | 0.00 | 0.24 | 1132.3 | 20.4 | 5.3 | 11 |
| 1.75 | h | set_2 | 0.00 | 0.12 | 1132.3 | 20.4 | 5.3 | 11 |
| 1.75 | h | set_3 | 0.00 | 0.08 | 1133.7 | 21.8 | 2.6 | 9 |
| 1.75 | lq | set_1 | 0.00 | 0.08 | 1130.7 | 18.8 | 1.2 | 6 |
| 1.75 | lq | set_2 | 0.00 | 0.08 | 1130.0 | 18.1 | 1.7 | 5 |
| 1.75 | lq | set_3 | 0.00 | 0.08 | 1127.8 | 15.8 | 5.3 | 4 |
| 1.75 | lp | set_1 | 0.00 | 0.24 | 1153.0 | 41.1 | 1.7 | 6 |
| 1.75 | lp | set_2 | 0.00 | 0.2 | 1177.4 | 65.4 | 9.0 | 4 |
| 1.75 | lp | set_3 | 0.00 | 0.2 | 1184.3 | 72.4 | 2.8 | 4 |
| 1.75 | lt | set_1 | 0.00 | 0.28 | 1130.4 | 18.4 | 1.4 | 9 |
| 1.75 | lt | set_2 | 0.00 | 0.28 | 1131.1 | 19.2 | 9.9 | 8 |
| 1.75 | lt | set_3 | 0.00 | 0.28 | 1129.1 | 17.2 | 2.7 | 7 |
| 1.75 | lh | set_1 | 0.00 | 0.24 | 1130.2 | 18.2 | 1.6 | 11 |
| 1.75 | lh | set_2 | 0.00 | 0.08 | 1137.8 | 25.8 | 3.6 | 13 |
| 1.75 | lh | set_3 | 0.00 | 0.08 | 1133.1 | 21.1 | 3.8 | 9 |
| 1.75 | qp | set_1 | 0.00 | 0.16 | 1134.7 | 22.7 | 1.7 | 6 |
| 1.75 | qp | set_2 | 0.00 | 0.16 | 1132.5 | 20.6 | 5.0 | 5 |
| 1.75 | qp | set_3 | 0.00 | 0.16 | 1132.3 | 20.4 | 5.5 | 5 |
| 1.75 | qt | set_1 | 0.00 | 0.36 | 1132.7 | 20.8 | 4.4 | 9 |
| 1.75 | qt | set_2 | 0.00 | 0.28 | 1130.3 | 18.3 | 1.5 | 8 |
| 1.75 | qt | set_3 | 0.00 | 0.28 | 1128.0 | 16.1 | 4.7 | 7 |
| 1.75 | qh | set_1 | 0.00 | 0.28 | 1124.6 | 12.7 | 2.6 | 9 |
| 1.75 | qh | set_2 | 0.00 | 0.08 | 1121.9 | 9.9 | 1.0 | 8 |
| 1.75 | qh | set_3 | 0.00 | 0.08 | 1122.1 | 10.2 | 9.2 | 5 |
| 1.75 | pt | set_1 | 0.00 | 0.36 | 1132.8 | 20.9 | 4.3 | 9 |
| 1.75 | pt | set_2 | 0.00 | 0.36 | 1133.3 | 21.3 | 3.4 | 9 |
| 1.75 | pt | set_3 | 0.00 | 0.32 | 1136.3 | 24.4 | 7.6 | 10 |
| 1.75 | ph | set_1 | 0.00 | 0.24 | 1143.2 | 31.2 | 2.4 | 14 |
| 1.75 | ph | set_2 | 0.00 | 0.12 | 1143.2 | 31.3 | 2.4 | 14 |
| 1.75 | ph | set_3 | 0.00 | 0.08 | 1131.4 | 19.5 | 8.7 | 8 |
| 1.75 | th | set_1 | 0.00 | 0.24 | 1129.6 | 17.6 | 2.2 | 12 |
| 1.75 | th | set_2 | 0.00 | 0.08 | 1129.6 | 17.6 | 2.2 | 12 |
| 1.75 | th | set_3 | 0.00 | 0.12 | 1128.5 | 16.6 | 3.8 | 9 |
| 1.75 | lqp | set_1 | 0.00 | 0.16 | 1134.4 | 22.5 | 1.9 | 6 |
| 1.75 | lqp | set_2 | 0.00 | 0.16 | 1132.1 | 20.2 | 6.4 | 5 |
| 1.75 | lqp | set_3 | 0.00 | 0.16 | 1132.4 | 20.5 | 5.3 | 5 |
| 1.75 | lqt | set_1 | 0.00 | 0.12 | 1127.1 | 15.2 | 7.6 | 7 |
| 1.75 | lqt | set_2 | 0.00 | 0.12 | 1127.0 | 15.1 | 7.9 | 6 |
| 1.75 | lqt | set_3 | 0.00 | 0.12 | 1124.8 | 12.9 | 2.4 | 5 |
| 1.75 | lqh | set_1 | 0.00 | 0.28 | 1126.3 | 14.3 | 1.1 | 9 |
| 1.75 | lqh | set_2 | 0.00 | 0.16 | 1120.6 | 8.7 | 1.9 | 7 |
| 1.75 | lqh | set_3 | 0.00 | 0.12 | 1123.8 | 11.8 | 4.1 | 5 |
| 1.75 | lpt | set_1 | 0.00 | 0.36 | 1132.8 | 20.9 | 4.4 | 9 |
| 1.75 | lpt | set_2 | 0.00 | 0.36 | 1130.5 | 18.6 | 1.4 | 8 |
| 1.75 | lpt | set_3 | 0.00 | 0.32 | 1130.9 | 19.0 | 1.1 | 8 |
| 1.75 | lph | set_1 | 0.00 | 0.24 | 1143.3 | 31.3 | 2.4 | 14 |
| 1.75 | lph | set_2 | 0.00 | 0.12 | 1143.3 | 31.4 | 2.4 | 14 |
| 1.75 | lph | set_3 | 0.00 | 0.08 | 1131.3 | 19.4 | 9.6 | 8 |
| 1.75 | qpt | set_1 | 0.00 | 0.2 | 1135.5 | 23.6 | 1.1 | 10 |
| 1.75 | qpt | set_2 | 0.00 | 0.2 | 1137.7 | 25.7 | 4.0 | 10 |
| 1.75 | qpt | set_3 | 0.00 | 0.2 | 1128.1 | 16.1 | 4.9 | 7 |
| 1.75 | qph | set_1 | 0.00 | 0.24 | 1128.7 | 16.7 | 3.6 | 10 |
| 1.75 | qph | set_2 | 0.00 | 0.08 | 1132.1 | 20.2 | 6.3 | 11 |
| 1.75 | qph | set_3 | 0.00 | 0.08 | 1123.1 | 11.1 | 6.0 | 5 |
| 1.75 | qth | set_1 | 0.00 | 0.24 | 1118.9 | 7.0 | 4.7 | 9 |
| 1.75 | qth | set_2 | 0.00 | 0.08 | 1116.1 | 4.1 | 1.9 | 8 |
| 1.75 | qth | set_3 | 0.00 | 0.08 | 1125.5 | 13.6 | 1.7 | 8 |
| 1.75 | pth | set_1 | 0.00 | 0.24 | 1125.5 | 13.6 | 1.7 | 11 |
| 1.75 | pth | set_2 | 0.00 | 0.08 | 1129.0 | 17.1 | 3.1 | 12 |
| 1.75 | pth | set_3 | 0.00 | 0.08 | 1125.0 | 13.1 | 2.2 | 8 |
| 1.75 | lqpt | set_1 | 0.00 | 0.2 | 1135.5 | 23.6 | 1.1 | 10 |
| 1.75 | lqpt | set_2 | 0.00 | 0.2 | 1134.2 | 22.3 | 2.2 | 9 |
| 1.75 | lqpt | set_3 | 0.00 | 0.2 | 1127.7 | 15.8 | 5.9 | 7 |
| 1.75 | lqph | set_1 | 0.00 | 0.24 | 1128.7 | 16.7 | 3.7 | 10 |
| 1.75 | lqph | set_2 | 0.00 | 0.08 | 1128.8 | 16.9 | 3.4 | 10 |
| 1.75 | lqph | set_3 | 0.00 | 0.08 | 1123.0 | 11.1 | 6.1 | 5 |
| 1.75 | lqth | set_1 | 0.00 | 0.24 | 1115.6 | 3.6 | 2.6 | 7 |
| 1.75 | lqth | set_2 | 0.00 | 0.16 | 1115.6 | 3.6 | 2.6 | 7 |
| 1.75 | lqth | set_3 | 0.00 | 0.12 | 1122.7 | 10.7 | 7.4 | 6 |
| 1.75 | lpth | set_1 | 0.00 | 0.24 | 1125.5 | 13.6 | 1.7 | 11 |
| 1.75 | lpth | set_2 | 0.00 | 0.08 | 1125.5 | 13.6 | 1.7 | 11 |
| 1.75 | lpth | set_3 | 0.00 | 0.08 | 1125.0 | 13.1 | 2.3 | 8 |
| 1.75 | lqpth | set_1 | 0.00 | 0.2 | 1116.8 | 4.9 | 1.3 | 8 |
| 1.75 | lqpth | set_2 | 0.00 | 0.08 | 1116.8 | 4.9 | 1.3 | 8 |
| 1.75 | lqpth | set_3 | 0.00 | 0.08 | 1120.4 | 8.5 | 2.3 | 6 |
| 2 | l | set_1 | 0.00 | 0.2 | 1172.8 | 60.9 | 9.6 | 4 |
| 2 | l | set_2 | 0.00 | 0.2 | 1176.9 | 65.0 | 1.2 | 3 |
| 2 | l | set_3 | 0.00 | 0.16 | 1181.8 | 69.8 | 1.1 | 3 |
| 2 | q | set_1 | 0.00 | 0.32 | 1171.6 | 59.7 | 1.7 | 5 |
| 2 | q | set_2 | 0.00 | 0.32 | 1171.6 | 59.7 | 1.7 | 4 |
| 2 | q | set_3 | 0.00 | 0.28 | 1186.7 | 74.7 | 9.6 | 3 |
| 2 | p | set_1 | 0.00 | 0.24 | 1151.4 | 39.5 | 4.3 | 4 |
| 2 | p | set_2 | 0.00 | 0.16 | 1176.1 | 64.1 | 1.9 | 3 |
| 2 | p | set_3 | 0.00 | 0.12 | 1186.0 | 74.0 | 1.3 | 2 |
| 2 | t | set_1 | 0.00 | 0.28 | 1145.5 | 33.6 | 8.4 | 12 |
| 2 | t | set_2 | 0.00 | 0.28 | 1145.5 | 33.6 | 8.4 | 12 |
| 2 | t | set_3 | 0.00 | 0.28 | 1155.5 | 43.5 | 5.8 | 13 |
| 2 | h | set_1 | 0.00 | 0.24 | 1131.5 | 19.6 | 9.2 | 10 |
| 2 | h | set_2 | 0.00 | 0.12 | 1131.5 | 19.6 | 9.2 | 10 |
| 2 | h | set_3 | 0.00 | 0.08 | 1132.6 | 20.6 | 5.4 | 8 |
| 2 | lq | set_1 | 0.00 | 0.08 | 1131.1 | 19.2 | 1.1 | 6 |
| 2 | lq | set_2 | 0.00 | 0.08 | 1130.4 | 18.4 | 1.6 | 5 |
| 2 | lq | set_3 | 0.00 | 0.08 | 1128.1 | 16.2 | 5.1 | 4 |
| 2 | lp | set_1 | 0.00 | 0.24 | 1154.7 | 42.8 | 8.4 | 6 |
| 2 | lp | set_2 | 0.00 | 0.2 | 1177.7 | 65.7 | 8.8 | 4 |
| 2 | lp | set_3 | 0.00 | 0.16 | 1184.4 | 72.5 | 3.0 | 4 |
| 2 | lt | set_1 | 0.00 | 0.2 | 1130.1 | 18.2 | 1.8 | 8 |
| 2 | lt | set_2 | 0.00 | 0.2 | 1131.2 | 19.2 | 1.1 | 7 |
| 2 | lt | set_3 | 0.00 | 0.28 | 1129.0 | 17.1 | 3.2 | 6 |
| 2 | lh | set_1 | 0.00 | 0.24 | 1132.9 | 21.0 | 4.7 | 11 |
| 2 | lh | set_2 | 0.00 | 0.08 | 1133.2 | 21.3 | 4.0 | 11 |
| 2 | lh | set_3 | 0.00 | 0.08 | 1132.5 | 20.5 | 5.8 | 8 |
| 2 | qp | set_1 | 0.00 | 0.16 | 1135.9 | 24.0 | 1.0 | 6 |
| 2 | qp | set_2 | 0.00 | 0.16 | 1133.4 | 21.5 | 3.6 | 5 |
| 2 | qp | set_3 | 0.00 | 0.16 | 1133.8 | 21.8 | 3.0 | 5 |
| 2 | qt | set_1 | 0.00 | 0.32 | 1128.8 | 16.9 | 3.7 | 6 |
| 2 | qt | set_2 | 0.00 | 0.28 | 1128.8 | 16.9 | 3.7 | 6 |
| 2 | qt | set_3 | 0.00 | 0.28 | 1128.8 | 16.9 | 3.7 | 6 |
| 2 | qh | set_1 | 0.00 | 0.28 | 1123.8 | 11.9 | 4.5 | 8 |
| 2 | qh | set_2 | 0.00 | 0.08 | 1124.9 | 13.0 | 2.6 | 8 |
| 2 | qh | set_3 | 0.00 | 0.08 | 1123.8 | 11.8 | 4.6 | 5 |
| 2 | pt | set_1 | 0.00 | 0.36 | 1136.6 | 24.6 | 7.7 | 9 |
| 2 | pt | set_2 | 0.00 | 0.36 | 1134.1 | 22.2 | 2.6 | 8 |
| 2 | pt | set_3 | 0.00 | 0.36 | 1139.3 | 27.4 | 1.9 | 9 |
| 2 | ph | set_1 | 0.00 | 0.24 | 1138.7 | 26.8 | 2.6 | 12 |
| 2 | ph | set_2 | 0.00 | 0.12 | 1135.3 | 23.4 | 1.4 | 11 |
| 2 | ph | set_3 | 0.00 | 0.08 | 1127.7 | 15.8 | 6.5 | 6 |
| 2 | th | set_1 | 0.00 | 0.24 | 1129.6 | 17.7 | 2.4 | 11 |
| 2 | th | set_2 | 0.00 | 0.08 | 1129.6 | 17.7 | 2.4 | 11 |
| 2 | th | set_3 | 0.00 | 0.08 | 1131.2 | 19.3 | 1.1 | 9 |
| 2 | lqp | set_1 | 0.00 | 0.16 | 1133.0 | 21.1 | 4.6 | 5 |
| 2 | lqp | set_2 | 0.00 | 0.16 | 1133.0 | 21.1 | 4.6 | 5 |
| 2 | lqp | set_3 | 0.00 | 0.16 | 1131.0 | 19.1 | 1.2 | 4 |
| 2 | lqt | set_1 | 0.00 | 0.12 | 1128.3 | 16.4 | 4.7 | 7 |
| 2 | lqt | set_2 | 0.00 | 0.12 | 1128.1 | 16.2 | 5.4 | 6 |
| 2 | lqt | set_3 | 0.00 | 0.12 | 1125.8 | 13.9 | 1.6 | 5 |
| 2 | lqh | set_1 | 0.00 | 0.28 | 1123.1 | 11.2 | 6.6 | 7 |
| 2 | lqh | set_2 | 0.00 | 0.16 | 1123.3 | 11.4 | 5.9 | 7 |
| 2 | lqh | set_3 | 0.00 | 0.08 | 1125.2 | 13.3 | 2.2 | 5 |
| 2 | lpt | set_1 | 0.00 | 0.32 | 1133.5 | 21.6 | 3.6 | 8 |
| 2 | lpt | set_2 | 0.00 | 0.32 | 1133.6 | 21.7 | 3.5 | 8 |
| 2 | lpt | set_3 | 0.00 | 0.28 | 1134.0 | 22.1 | 2.8 | 8 |
| 2 | lph | set_1 | 0.00 | 0.24 | 1142.4 | 30.5 | 4.2 | 13 |
| 2 | lph | set_2 | 0.00 | 0.12 | 1142.4 | 30.5 | 4.3 | 13 |
| 2 | lph | set_3 | 0.00 | 0.08 | 1127.7 | 15.8 | 6.7 | 6 |
| 2 | qpt | set_1 | 0.00 | 0.2 | 1137.1 | 25.1 | 6.2 | 9 |
| 2 | qpt | set_2 | 0.00 | 0.2 | 1134.9 | 23.0 | 1.8 | 8 |
| 2 | qpt | set_3 | 0.00 | 0.2 | 1130.6 | 18.7 | 1.5 | 7 |
| 2 | qph | set_1 | 0.00 | 0.24 | 1125.1 | 13.2 | 2.4 | 8 |
| 2 | qph | set_2 | 0.00 | 0.08 | 1122.5 | 10.6 | 8.9 | 7 |
| 2 | qph | set_3 | 0.00 | 0.08 | 1124.2 | 12.3 | 3.8 | 5 |
| 2 | qth | set_1 | 0.00 | 0.28 | 1121.6 | 9.7 | 1.4 | 9 |
| 2 | qth | set_2 | 0.00 | 0.08 | 1118.8 | 6.9 | 5.8 | 8 |
| 2 | qth | set_3 | 0.00 | 0.08 | 1125.2 | 13.3 | 2.3 | 7 |
| 2 | pth | set_1 | 0.00 | 0.24 | 1128.7 | 16.8 | 4.0 | 11 |
| 2 | pth | set_2 | 0.00 | 0.08 | 1128.8 | 16.9 | 4.0 | 11 |
| 2 | pth | set_3 | 0.00 | 0.08 | 1124.6 | 12.7 | 3.2 | 7 |
| 2 | lqpt | set_1 | 0.00 | 0.16 | 1136.8 | 24.8 | 7.4 | 9 |
| 2 | lqpt | set_2 | 0.00 | 0.16 | 1134.1 | 22.2 | 2.7 | 8 |
| 2 | lqpt | set_3 | 0.00 | 0.2 | 1130.7 | 18.8 | 1.5 | 7 |
| 2 | lqph | set_1 | 0.00 | 0.24 | 1125.1 | 13.2 | 2.5 | 8 |
| 2 | lqph | set_2 | 0.00 | 0.08 | 1125.4 | 13.5 | 2.1 | 8 |
| 2 | lqph | set_3 | 0.00 | 0.08 | 1124.2 | 12.3 | 4.0 | 5 |
| 2 | lqth | set_1 | 0.00 | 0.28 | 1117.8 | 5.8 | 9.9 | 7 |
| 2 | lqth | set_2 | 0.00 | 0.16 | 1117.8 | 5.8 | 1.0 | 7 |
| 2 | lqth | set_3 | 0.00 | 0.12 | 1124.6 | 12.7 | 3.2 | 6 |
| 2 | lpth | set_1 | 0.00 | 0.24 | 1128.8 | 16.9 | 4.0 | 11 |
| 2 | lpth | set_2 | 0.00 | 0.08 | 1125.5 | 13.5 | 2.1 | 10 |
| 2 | lpth | set_3 | 0.00 | 0.08 | 1124.6 | 12.7 | 3.3 | 7 |
| 2 | lqpth | set_1 | 0.00 | 0.24 | 1122.6 | 10.7 | 9.0 | 9 |
| 2 | lqpth | set_2 | 0.00 | 0.08 | 1122.6 | 10.7 | 9.0 | 9 |
| 2 | lqpth | set_3 | 0.00 | 0.08 | 1125.1 | 13.2 | 2.5 | 7 |
| 3 | l | set_1 | 0.00 | 0.16 | 1173.6 | 61.7 | 7.6 | 4 |
| 3 | l | set_2 | 0.00 | 0.16 | 1177.1 | 65.2 | 1.3 | 3 |
| 3 | l | set_3 | 0.00 | 0.16 | 1181.9 | 70.0 | 1.2 | 3 |
| 3 | q | set_1 | 0.00 | 0.28 | 1173.9 | 62.0 | 6.4 | 5 |
| 3 | q | set_2 | 0.00 | 0.28 | 1174.0 | 62.1 | 6.2 | 4 |
| 3 | q | set_3 | 0.00 | 0.28 | 1187.3 | 75.3 | 8.3 | 3 |
| 3 | p | set_1 | 0.00 | 0.16 | 1159.7 | 47.7 | 8.1 | 5 |
| 3 | p | set_2 | 0.00 | 0.16 | 1176.9 | 65.0 | 1.4 | 3 |
| 3 | p | set_3 | 0.00 | 0.12 | 1186.3 | 74.3 | 1.3 | 2 |
| 3 | t | set_1 | 0.00 | 0.28 | 1163.6 | 51.7 | 1.1 | 9 |
| 3 | t | set_2 | 0.00 | 0.28 | 1163.6 | 51.7 | 1.1 | 9 |
| 3 | t | set_3 | 0.00 | 0.28 | 1163.6 | 51.7 | 1.1 | 9 |
| 3 | h | set_1 | 0.00 | 0.12 | 1132.4 | 20.4 | 7.0 | 7 |
| 3 | h | set_2 | 0.00 | 0.08 | 1132.4 | 20.4 | 7.0 | 7 |
| 3 | h | set_3 | 0.00 | 0.08 | 1143.1 | 31.2 | 3.2 | 9 |
| 3 | lq | set_1 | 0.00 | 0.08 | 1130.4 | 18.5 | 1.8 | 5 |
| 3 | lq | set_2 | 0.00 | 0.08 | 1132.0 | 20.1 | 8.5 | 5 |
| 3 | lq | set_3 | 0.00 | 0.08 | 1129.7 | 17.8 | 2.6 | 4 |
| 3 | lp | set_1 | 0.00 | 0.16 | 1159.1 | 47.2 | 1.0 | 5 |
| 3 | lp | set_2 | 0.00 | 0.16 | 1179.2 | 67.3 | 4.7 | 4 |
| 3 | lp | set_3 | 0.00 | 0.16 | 1185.1 | 73.1 | 2.5 | 4 |
| 3 | lt | set_1 | 0.00 | 0.2 | 1138.9 | 27.0 | 2.6 | 8 |
| 3 | lt | set_2 | 0.00 | 0.2 | 1139.4 | 27.5 | 2.0 | 7 |
| 3 | lt | set_3 | 0.00 | 0.2 | 1137.4 | 25.5 | 5.7 | 6 |
| 3 | lh | set_1 | 0.00 | 0.12 | 1136.2 | 24.3 | 1.0 | 9 |
| 3 | lh | set_2 | 0.00 | 0.12 | 1133.4 | 21.4 | 4.4 | 8 |
| 3 | lh | set_3 | 0.00 | 0.08 | 1141.9 | 30.0 | 6.0 | 8 |
| 3 | qp | set_1 | 0.00 | 0.2 | 1137.6 | 25.6 | 5.4 | 5 |
| 3 | qp | set_2 | 0.00 | 0.2 | 1137.6 | 25.6 | 5.4 | 5 |
| 3 | qp | set_3 | 0.00 | 0.12 | 1135.6 | 23.6 | 1.4 | 4 |
| 3 | qt | set_1 | 0.00 | 0.24 | 1142.2 | 30.3 | 5.3 | 7 |
| 3 | qt | set_2 | 0.00 | 0.24 | 1142.2 | 30.3 | 5.3 | 7 |
| 3 | qt | set_3 | 0.00 | 0.24 | 1139.5 | 27.6 | 2.0 | 6 |
| 3 | qh | set_1 | 0.00 | 0.28 | 1128.6 | 16.7 | 4.8 | 7 |
| 3 | qh | set_2 | 0.00 | 0.08 | 1126.6 | 14.7 | 1.3 | 6 |
| 3 | qh | set_3 | 0.00 | 0.08 | 1131.3 | 19.3 | 1.2 | 5 |
| 3 | pt | set_1 | 0.00 | 0.24 | 1142.3 | 30.4 | 5.0 | 7 |
| 3 | pt | set_2 | 0.00 | 0.24 | 1142.4 | 30.4 | 5.0 | 7 |
| 3 | pt | set_3 | 0.00 | 0.32 | 1145.1 | 33.2 | 1.2 | 5 |
| 3 | ph | set_1 | 0.00 | 0.16 | 1136.3 | 24.3 | 1.0 | 8 |
| 3 | ph | set_2 | 0.00 | 0.08 | 1133.5 | 21.5 | 4.3 | 7 |
| 3 | ph | set_3 | 0.00 | 0.08 | 1140.4 | 28.4 | 1.3 | 8 |
| 3 | th | set_1 | 0.00 | 0.12 | 1136.5 | 24.6 | 9.4 | 9 |
| 3 | th | set_2 | 0.00 | 0.08 | 1136.5 | 24.6 | 9.4 | 9 |
| 3 | th | set_3 | 0.00 | 0.08 | 1141.8 | 29.9 | 6.7 | 9 |
| 3 | lqp | set_1 | 0.00 | 0.2 | 1137.2 | 25.2 | 6.8 | 5 |
| 3 | lqp | set_2 | 0.00 | 0.2 | 1137.1 | 25.2 | 6.9 | 5 |
| 3 | lqp | set_3 | 0.00 | 0.12 | 1135.3 | 23.4 | 1.7 | 4 |
| 3 | lqt | set_1 | 0.00 | 0.08 | 1133.9 | 21.9 | 3.5 | 7 |
| 3 | lqt | set_2 | 0.00 | 0.08 | 1133.0 | 21.1 | 5.4 | 6 |
| 3 | lqt | set_3 | 0.00 | 0.08 | 1130.7 | 18.8 | 1.7 | 5 |
| 3 | lqh | set_1 | 0.00 | 0.28 | 1126.8 | 14.9 | 1.2 | 6 |
| 3 | lqh | set_2 | 0.00 | 0.08 | 1126.8 | 14.9 | 1.2 | 6 |
| 3 | lqh | set_3 | 0.00 | 0.08 | 1132.0 | 20.1 | 9.0 | 5 |
| 3 | lpt | set_1 | 0.00 | 0.2 | 1138.8 | 26.9 | 3.0 | 6 |
| 3 | lpt | set_2 | 0.00 | 0.2 | 1138.8 | 26.9 | 3.0 | 6 |
| 3 | lpt | set_3 | 0.00 | 0.2 | 1142.5 | 30.5 | 4.9 | 7 |
| 3 | lph | set_1 | 0.00 | 0.16 | 1133.5 | 21.5 | 4.4 | 7 |
| 3 | lph | set_2 | 0.00 | 0.08 | 1130.8 | 18.8 | 1.7 | 6 |
| 3 | lph | set_3 | 0.00 | 0.08 | 1140.6 | 28.7 | 1.2 | 8 |
| 3 | qpt | set_1 | 0.00 | 0.2 | 1139.6 | 27.7 | 2.0 | 7 |
| 3 | qpt | set_2 | 0.00 | 0.2 | 1139.6 | 27.7 | 2.0 | 7 |
| 3 | qpt | set_3 | 0.00 | 0.16 | 1137.5 | 25.5 | 6.0 | 6 |
| 3 | qph | set_1 | 0.00 | 0.16 | 1133.2 | 21.3 | 5.0 | 8 |
| 3 | qph | set_2 | 0.00 | 0.08 | 1133.3 | 21.3 | 4.9 | 8 |
| 3 | qph | set_3 | 0.00 | 0.08 | 1135.1 | 23.2 | 1.9 | 7 |
| 3 | qth | set_1 | 0.00 | 0.28 | 1130.1 | 18.2 | 2.4 | 8 |
| 3 | qth | set_2 | 0.00 | 0.08 | 1127.3 | 15.4 | 9.8 | 7 |
| 3 | qth | set_3 | 0.00 | 0.08 | 1133.0 | 21.1 | 5.7 | 6 |
| 3 | pth | set_1 | 0.00 | 0.16 | 1132.4 | 20.4 | 7.9 | 8 |
| 3 | pth | set_2 | 0.00 | 0.08 | 1132.4 | 20.4 | 7.8 | 8 |
| 3 | pth | set_3 | 0.00 | 0.08 | 1134.4 | 22.5 | 2.9 | 7 |
| 3 | lqpt | set_1 | 0.00 | 0.2 | 1139.3 | 27.4 | 2.4 | 7 |
| 3 | lqpt | set_2 | 0.00 | 0.2 | 1139.3 | 27.4 | 2.4 | 7 |
| 3 | lqpt | set_3 | 0.00 | 0.12 | 1137.2 | 25.3 | 7.0 | 6 |
| 3 | lqph | set_1 | 0.00 | 0.16 | 1136.3 | 24.4 | 1.1 | 9 |
| 3 | lqph | set_2 | 0.00 | 0.08 | 1133.3 | 21.3 | 5.1 | 8 |
| 3 | lqph | set_3 | 0.00 | 0.08 | 1135.6 | 23.7 | 1.5 | 7 |
| 3 | lqth | set_1 | 0.00 | 0.28 | 1127.6 | 15.7 | 8.6 | 7 |
| 3 | lqth | set_2 | 0.00 | 0.08 | 1127.6 | 15.7 | 8.6 | 7 |
| 3 | lqth | set_3 | 0.00 | 0.08 | 1130.7 | 18.8 | 1.8 | 5 |
| 3 | lpth | set_1 | 0.00 | 0.16 | 1132.4 | 20.5 | 7.9 | 8 |
| 3 | lpth | set_2 | 0.00 | 0.08 | 1132.4 | 20.5 | 7.9 | 8 |
| 3 | lpth | set_3 | 0.00 | 0.08 | 1137.4 | 25.5 | 6.5 | 8 |
| 3 | lqpth | set_1 | 0.00 | 0.16 | 1128.7 | 16.7 | 5.2 | 7 |
| 3 | lqpth | set_2 | 0.00 | 0.08 | 1128.7 | 16.7 | 5.2 | 7 |
| 3 | lqpth | set_3 | 0.00 | 0.08 | 1137.7 | 25.8 | 5.7 | 8 |
| 4 | l | set_1 | 0.00 | 0.16 | 1174.6 | 62.7 | 5.5 | 4 |
| 4 | l | set_2 | 0.00 | 0.16 | 1177.4 | 65.5 | 1.3 | 3 |
| 4 | l | set_3 | 0.00 | 0.16 | 1182.1 | 70.1 | 1.3 | 3 |
| 4 | q | set_1 | 0.00 | 0.28 | 1177.0 | 65.0 | 1.7 | 5 |
| 4 | q | set_2 | 0.00 | 0.28 | 1177.1 | 65.2 | 1.6 | 4 |
| 4 | q | set_3 | 0.00 | 0.28 | 1188.0 | 76.1 | 6.8 | 3 |
| 4 | p | set_1 | 0.00 | 0.16 | 1168.2 | 56.3 | 1.3 | 5 |
| 4 | p | set_2 | 0.00 | 0.16 | 1178.1 | 66.2 | 9.8 | 3 |
| 4 | p | set_3 | 0.00 | 0.12 | 1186.7 | 74.8 | 1.3 | 2 |
| 4 | t | set_1 | 0.00 | 0.28 | 1166.9 | 55.0 | 2.6 | 5 |
| 4 | t | set_2 | 0.00 | 0.28 | 1166.9 | 55.0 | 2.6 | 5 |
| 4 | t | set_3 | 0.00 | 0.28 | 1166.9 | 55.0 | 2.6 | 5 |
| 4 | h | set_1 | 0.00 | 0.08 | 1131.5 | 19.5 | 1.3 | 4 |
| 4 | h | set_2 | 0.00 | 0.08 | 1131.5 | 19.5 | 1.3 | 4 |
| 4 | h | set_3 | 0.00 | 0.08 | 1151.6 | 39.7 | 5.6 | 9 |
| 4 | lq | set_1 | 0.00 | 0.08 | 1132.7 | 20.8 | 7.2 | 5 |
| 4 | lq | set_2 | 0.00 | 0.08 | 1133.9 | 22.0 | 3.9 | 5 |
| 4 | lq | set_3 | 0.00 | 0.08 | 1131.6 | 19.6 | 1.2 | 4 |
| 4 | lp | set_1 | 0.00 | 0.16 | 1163.6 | 51.7 | 1.4 | 4 |
| 4 | lp | set_2 | 0.00 | 0.16 | 1178.7 | 66.8 | 7.5 | 3 |
| 4 | lp | set_3 | 0.00 | 0.12 | 1183.5 | 71.6 | 6.6 | 3 |
| 4 | lt | set_1 | 0.00 | 0.16 | 1148.6 | 36.7 | 2.5 | 8 |
| 4 | lt | set_2 | 0.00 | 0.16 | 1148.6 | 36.7 | 2.5 | 7 |
| 4 | lt | set_3 | 0.00 | 0.16 | 1146.7 | 34.8 | 6.6 | 6 |
| 4 | lh | set_1 | 0.00 | 0.16 | 1142.7 | 30.8 | 4.8 | 8 |
| 4 | lh | set_2 | 0.00 | 0.12 | 1142.7 | 30.8 | 4.8 | 8 |
| 4 | lh | set_3 | 0.00 | 0.08 | 1142.9 | 31.0 | 4.4 | 6 |
| 4 | qp | set_1 | 0.00 | 0.2 | 1142.2 | 30.2 | 6.5 | 5 |
| 4 | qp | set_2 | 0.00 | 0.2 | 1142.2 | 30.2 | 6.5 | 5 |
| 4 | qp | set_3 | 0.00 | 0.16 | 1140.3 | 28.4 | 1.6 | 4 |
| 4 | qt | set_1 | 0.00 | 0.28 | 1152.3 | 40.3 | 4.2 | 6 |
| 4 | qt | set_2 | 0.00 | 0.28 | 1152.3 | 40.3 | 4.2 | 6 |
| 4 | qt | set_3 | 0.00 | 0.28 | 1152.3 | 40.3 | 4.2 | 6 |
| 4 | qh | set_1 | 0.00 | 0.16 | 1134.5 | 22.6 | 3.0 | 6 |
| 4 | qh | set_2 | 0.00 | 0.12 | 1134.6 | 22.7 | 2.9 | 6 |
| 4 | qh | set_3 | 0.00 | 0.12 | 1133.3 | 21.4 | 5.6 | 4 |
| 4 | pt | set_1 | 0.00 | 0.16 | 1150.8 | 38.9 | 8.9 | 6 |
| 4 | pt | set_2 | 0.00 | 0.16 | 1150.8 | 38.9 | 8.9 | 6 |
| 4 | pt | set_3 | 0.00 | 0.16 | 1155.5 | 43.6 | 8.3 | 5 |
| 4 | ph | set_1 | 0.00 | 0.16 | 1136.0 | 24.0 | 1.4 | 5 |
| 4 | ph | set_2 | 0.00 | 0.08 | 1136.0 | 24.0 | 1.4 | 5 |
| 4 | ph | set_3 | 0.00 | 0.08 | 1142.7 | 30.8 | 5.1 | 6 |
| 4 | th | set_1 | 0.00 | 0.08 | 1131.4 | 19.5 | 1.4 | 4 |
| 4 | th | set_2 | 0.00 | 0.08 | 1131.4 | 19.5 | 1.4 | 4 |
| 4 | th | set_3 | 0.00 | 0.08 | 1145.0 | 33.1 | 1.6 | 7 |
| 4 | lqp | set_1 | 0.00 | 0.2 | 1141.7 | 29.8 | 8.4 | 5 |
| 4 | lqp | set_2 | 0.00 | 0.2 | 1141.7 | 29.8 | 8.4 | 5 |
| 4 | lqp | set_3 | 0.00 | 0.16 | 1140.0 | 28.1 | 1.9 | 4 |
| 4 | lqt | set_1 | 0.00 | 0.08 | 1132.7 | 20.8 | 7.8 | 5 |
| 4 | lqt | set_2 | 0.00 | 0.08 | 1133.9 | 22.0 | 4.2 | 5 |
| 4 | lqt | set_3 | 0.00 | 0.08 | 1131.6 | 19.6 | 1.3 | 4 |
| 4 | lqh | set_1 | 0.00 | 0.2 | 1136.8 | 24.8 | 1.0 | 7 |
| 4 | lqh | set_2 | 0.00 | 0.08 | 1133.6 | 21.6 | 5.1 | 6 |
| 4 | lqh | set_3 | 0.00 | 0.08 | 1131.6 | 19.6 | 1.4 | 4 |
| 4 | lpt | set_1 | 0.00 | 0.16 | 1149.7 | 37.7 | 1.6 | 6 |
| 4 | lpt | set_2 | 0.00 | 0.16 | 1149.7 | 37.7 | 1.6 | 6 |
| 4 | lpt | set_3 | 0.00 | 0.12 | 1153.8 | 41.8 | 2.1 | 7 |
| 4 | lph | set_1 | 0.00 | 0.16 | 1136.0 | 24.0 | 1.5 | 5 |
| 4 | lph | set_2 | 0.00 | 0.08 | 1136.0 | 24.0 | 1.5 | 5 |
| 4 | lph | set_3 | 0.00 | 0.08 | 1147.3 | 35.3 | 5.5 | 7 |
| 4 | qpt | set_1 | 0.00 | 0.2 | 1144.5 | 32.6 | 2.1 | 6 |
| 4 | qpt | set_2 | 0.00 | 0.2 | 1144.5 | 32.6 | 2.1 | 6 |
| 4 | qpt | set_3 | 0.00 | 0.16 | 1142.4 | 30.5 | 6.3 | 5 |
| 4 | qph | set_1 | 0.00 | 0.16 | 1137.4 | 25.4 | 7.8 | 7 |
| 4 | qph | set_2 | 0.00 | 0.08 | 1137.2 | 25.3 | 8.4 | 7 |
| 4 | qph | set_3 | 0.00 | 0.08 | 1137.0 | 25.1 | 9.4 | 5 |
| 4 | qth | set_1 | 0.00 | 0.16 | 1134.5 | 22.6 | 3.2 | 6 |
| 4 | qth | set_2 | 0.00 | 0.12 | 1134.5 | 22.6 | 3.2 | 6 |
| 4 | qth | set_3 | 0.00 | 0.12 | 1133.3 | 21.4 | 6.1 | 4 |
| 4 | pth | set_1 | 0.00 | 0.16 | 1137.9 | 25.9 | 6.2 | 6 |
| 4 | pth | set_2 | 0.00 | 0.08 | 1137.8 | 25.9 | 6.3 | 6 |
| 4 | pth | set_3 | 0.00 | 0.08 | 1144.8 | 32.9 | 1.9 | 7 |
| 4 | lqpt | set_1 | 0.00 | 0.2 | 1144.1 | 32.2 | 2.7 | 6 |
| 4 | lqpt | set_2 | 0.00 | 0.2 | 1144.1 | 32.2 | 2.7 | 6 |
| 4 | lqpt | set_3 | 0.00 | 0.16 | 1142.2 | 30.3 | 7.2 | 5 |
| 4 | lqph | set_1 | 0.00 | 0.16 | 1138.0 | 26.0 | 6.0 | 7 |
| 4 | lqph | set_2 | 0.00 | 0.08 | 1140.7 | 28.7 | 1.5 | 8 |
| 4 | lqph | set_3 | 0.00 | 0.08 | 1137.8 | 25.9 | 6.5 | 5 |
| 4 | lqth | set_1 | 0.00 | 0.2 | 1136.9 | 24.9 | 1.0 | 7 |
| 4 | lqth | set_2 | 0.00 | 0.08 | 1133.5 | 21.6 | 5.6 | 6 |
| 4 | lqth | set_3 | 0.00 | 0.08 | 1131.6 | 19.6 | 1.5 | 4 |
| 4 | lpth | set_1 | 0.00 | 0.16 | 1137.9 | 25.9 | 6.4 | 6 |
| 4 | lpth | set_2 | 0.00 | 0.08 | 1137.9 | 25.9 | 6.4 | 6 |
| 4 | lpth | set_3 | 0.00 | 0.08 | 1152.3 | 40.4 | 4.6 | 9 |
| 4 | lqpth | set_1 | 0.00 | 0.16 | 1135.2 | 23.2 | 2.5 | 6 |
| 4 | lqpth | set_2 | 0.00 | 0.08 | 1137.8 | 25.9 | 6.7 | 7 |
| 4 | lqpth | set_3 | 0.00 | 0.08 | 1140.6 | 28.6 | 1.6 | 6 |
| 5 | l | set_1 | 0.00 | 0.16 | 1175.8 | 63.9 | 3.8 | 4 |
| 5 | l | set_2 | 0.00 | 0.16 | 1177.8 | 65.9 | 1.4 | 3 |
| 5 | l | set_3 | 0.00 | 0.16 | 1182.3 | 70.3 | 1.5 | 3 |
| 5 | q | set_1 | 0.00 | 0.32 | 1180.5 | 68.6 | 3.5 | 5 |
| 5 | q | set_2 | 0.00 | 0.32 | 1180.8 | 68.9 | 3.1 | 4 |
| 5 | q | set_3 | 0.00 | 0.32 | 1189.0 | 77.0 | 5.3 | 3 |
| 5 | p | set_1 | 0.00 | 0.16 | 1173.7 | 61.8 | 1.1 | 4 |
| 5 | p | set_2 | 0.00 | 0.16 | 1179.5 | 67.5 | 6.1 | 3 |
| 5 | p | set_3 | 0.00 | 0.12 | 1187.2 | 75.3 | 1.2 | 2 |
| 5 | t | set_1 | 0.00 | 0.28 | 1180.4 | 68.5 | 3.8 | 5 |
| 5 | t | set_2 | 0.00 | 0.28 | 1180.4 | 68.5 | 3.8 | 5 |
| 5 | t | set_3 | 0.00 | 0.28 | 1180.4 | 68.5 | 3.8 | 5 |
| 5 | h | set_1 | 0.00 | 0.08 | 1136.3 | 24.3 | 1.5 | 4 |
| 5 | h | set_2 | 0.00 | 0.08 | 1136.3 | 24.3 | 1.5 | 4 |
| 5 | h | set_3 | 0.00 | 0.08 | 1146.7 | 34.8 | 8.3 | 5 |
| 5 | lq | set_1 | 0.00 | 0.08 | 1135.3 | 23.4 | 2.4 | 5 |
| 5 | lq | set_2 | 0.00 | 0.08 | 1136.0 | 24.1 | 1.7 | 5 |
| 5 | lq | set_3 | 0.00 | 0.08 | 1133.6 | 21.7 | 5.8 | 4 |
| 5 | lp | set_1 | 0.00 | 0.16 | 1171.6 | 59.7 | 3.2 | 4 |
| 5 | lp | set_2 | 0.00 | 0.16 | 1179.7 | 67.8 | 5.7 | 3 |
| 5 | lp | set_3 | 0.00 | 0.12 | 1184.5 | 72.6 | 5.1 | 3 |
| 5 | lt | set_1 | 0.00 | 0.16 | 1159.2 | 47.3 | 1.6 | 8 |
| 5 | lt | set_2 | 0.00 | 0.16 | 1158.6 | 46.7 | 2.1 | 7 |
| 5 | lt | set_3 | 0.00 | 0.16 | 1156.9 | 44.9 | 5.2 | 6 |
| 5 | lh | set_1 | 0.00 | 0.12 | 1150.1 | 38.2 | 1.5 | 7 |
| 5 | lh | set_2 | 0.00 | 0.08 | 1152.9 | 40.9 | 3.8 | 8 |
| 5 | lh | set_3 | 0.00 | 0.08 | 1148.8 | 36.8 | 3.0 | 6 |
| 5 | qp | set_1 | 0.00 | 0.16 | 1147.0 | 35.1 | 7.2 | 5 |
| 5 | qp | set_2 | 0.00 | 0.16 | 1147.0 | 35.1 | 7.2 | 5 |
| 5 | qp | set_3 | 0.00 | 0.16 | 1145.3 | 33.4 | 1.7 | 4 |
| 5 | qt | set_1 | 0.00 | 0.32 | 1153.6 | 41.6 | 2.7 | 4 |
| 5 | qt | set_2 | 0.00 | 0.32 | 1153.6 | 41.6 | 2.8 | 4 |
| 5 | qt | set_3 | 0.00 | 0.32 | 1153.6 | 41.6 | 2.8 | 4 |
| 5 | qh | set_1 | 0.00 | 0.12 | 1138.1 | 26.2 | 6.4 | 5 |
| 5 | qh | set_2 | 0.00 | 0.12 | 1138.1 | 26.2 | 6.4 | 5 |
| 5 | qh | set_3 | 0.00 | 0.12 | 1138.4 | 26.5 | 5.4 | 5 |
| 5 | pt | set_1 | 0.00 | 0.16 | 1159.5 | 47.6 | 1.4 | 5 |
| 5 | pt | set_2 | 0.00 | 0.16 | 1159.5 | 47.6 | 1.4 | 5 |
| 5 | pt | set_3 | 0.00 | 0.12 | 1162.5 | 50.6 | 3.2 | 4 |
| 5 | ph | set_1 | 0.00 | 0.16 | 1137.1 | 25.2 | 1.0 | 3 |
| 5 | ph | set_2 | 0.00 | 0.12 | 1137.1 | 25.2 | 1.0 | 3 |
| 5 | ph | set_3 | 0.00 | 0.08 | 1148.6 | 36.7 | 3.3 | 5 |
| 5 | th | set_1 | 0.00 | 0.08 | 1138.8 | 26.8 | 4.7 | 5 |
| 5 | th | set_2 | 0.00 | 0.08 | 1138.8 | 26.8 | 4.7 | 5 |
| 5 | th | set_3 | 0.00 | 0.08 | 1146.8 | 34.9 | 8.4 | 5 |
| 5 | lqp | set_1 | 0.00 | 0.16 | 1146.6 | 34.7 | 9.4 | 5 |
| 5 | lqp | set_2 | 0.00 | 0.16 | 1146.6 | 34.7 | 9.4 | 5 |
| 5 | lqp | set_3 | 0.00 | 0.16 | 1145.0 | 33.1 | 2.0 | 4 |
| 5 | lqt | set_1 | 0.00 | 0.08 | 1135.3 | 23.3 | 2.7 | 5 |
| 5 | lqt | set_2 | 0.00 | 0.08 | 1136.0 | 24.1 | 1.8 | 5 |
| 5 | lqt | set_3 | 0.00 | 0.08 | 1133.6 | 21.7 | 6.4 | 4 |
| 5 | lqh | set_1 | 0.00 | 0.08 | 1135.3 | 23.3 | 2.7 | 5 |
| 5 | lqh | set_2 | 0.00 | 0.08 | 1136.0 | 24.1 | 1.9 | 5 |
| 5 | lqh | set_3 | 0.00 | 0.08 | 1133.6 | 21.7 | 6.5 | 4 |
| 5 | lpt | set_1 | 0.00 | 0.16 | 1158.5 | 46.6 | 2.5 | 5 |
| 5 | lpt | set_2 | 0.00 | 0.16 | 1158.5 | 46.6 | 2.5 | 5 |
| 5 | lpt | set_3 | 0.00 | 0.12 | 1160.0 | 48.1 | 1.2 | 5 |
| 5 | lph | set_1 | 0.00 | 0.16 | 1139.9 | 28.0 | 2.7 | 4 |
| 5 | lph | set_2 | 0.00 | 0.08 | 1142.4 | 30.4 | 8.3 | 5 |
| 5 | lph | set_3 | 0.00 | 0.08 | 1152.9 | 40.9 | 4.3 | 6 |
| 5 | qpt | set_1 | 0.00 | 0.16 | 1147.1 | 35.1 | 7.9 | 5 |
| 5 | qpt | set_2 | 0.00 | 0.16 | 1147.0 | 35.1 | 8.0 | 5 |
| 5 | qpt | set_3 | 0.00 | 0.16 | 1147.7 | 35.7 | 5.9 | 5 |
| 5 | qph | set_1 | 0.00 | 0.16 | 1141.4 | 29.5 | 1.3 | 6 |
| 5 | qph | set_2 | 0.00 | 0.08 | 1141.4 | 29.5 | 1.3 | 6 |
| 5 | qph | set_3 | 0.00 | 0.08 | 1142.8 | 30.9 | 6.8 | 5 |
| 5 | qth | set_1 | 0.00 | 0.12 | 1138.1 | 26.2 | 7.1 | 5 |
| 5 | qth | set_2 | 0.00 | 0.12 | 1138.1 | 26.2 | 7.1 | 5 |
| 5 | qth | set_3 | 0.00 | 0.12 | 1136.0 | 24.1 | 2.0 | 4 |
| 5 | pth | set_1 | 0.00 | 0.16 | 1139.5 | 27.6 | 3.5 | 4 |
| 5 | pth | set_2 | 0.00 | 0.16 | 1139.5 | 27.6 | 3.5 | 4 |
| 5 | pth | set_3 | 0.00 | 0.08 | 1146.1 | 34.1 | 1.3 | 4 |
| 5 | lqpt | set_1 | 0.00 | 0.16 | 1146.6 | 34.7 | 1.0 | 5 |
| 5 | lqpt | set_2 | 0.00 | 0.16 | 1146.6 | 34.7 | 1.0 | 5 |
| 5 | lqpt | set_3 | 0.00 | 0.16 | 1145.0 | 33.1 | 2.2 | 4 |
| 5 | lqph | set_1 | 0.00 | 0.16 | 1141.8 | 29.8 | 1.1 | 6 |
| 5 | lqph | set_2 | 0.00 | 0.08 | 1139.1 | 27.2 | 4.3 | 5 |
| 5 | lqph | set_3 | 0.00 | 0.08 | 1139.9 | 27.9 | 3.0 | 4 |
| 5 | lqth | set_1 | 0.00 | 0.08 | 1135.3 | 23.4 | 3.0 | 5 |
| 5 | lqth | set_2 | 0.00 | 0.08 | 1136.0 | 24.1 | 2.1 | 5 |
| 5 | lqth | set_3 | 0.00 | 0.08 | 1133.6 | 21.7 | 7.1 | 4 |
| 5 | lpth | set_1 | 0.00 | 0.16 | 1142.5 | 30.6 | 8.2 | 5 |
| 5 | lpth | set_2 | 0.00 | 0.08 | 1142.4 | 30.5 | 8.7 | 5 |
| 5 | lpth | set_3 | 0.00 | 0.08 | 1150.2 | 38.3 | 1.8 | 5 |
| 5 | lqpth | set_1 | 0.00 | 0.16 | 1141.9 | 30.0 | 1.1 | 6 |
| 5 | lqpth | set_2 | 0.00 | 0.08 | 1141.8 | 29.9 | 1.1 | 6 |
| 5 | lqpth | set_3 | 0.00 | 0.08 | 1142.4 | 30.5 | 8.8 | 5 |
| 6 | l | set_1 | 0.00 | 0.16 | 1177.2 | 65.2 | 2.5 | 4 |
| 6 | l | set_2 | 0.00 | 0.16 | 1180.6 | 68.7 | 4.5 | 4 |
| 6 | l | set_3 | 0.00 | 0.16 | 1182.5 | 70.6 | 1.7 | 3 |
| 6 | q | set_1 | 0.00 | 0.32 | 1184.6 | 72.6 | 6.3 | 5 |
| 6 | q | set_2 | 0.00 | 0.32 | 1185.0 | 73.1 | 5.0 | 4 |
| 6 | q | set_3 | 0.00 | 0.32 | 1190.0 | 78.1 | 4.1 | 3 |
| 6 | p | set_1 | 0.00 | 0.16 | 1182.6 | 70.7 | 1.7 | 4 |
| 6 | p | set_2 | 0.00 | 0.16 | 1181.0 | 69.1 | 3.7 | 3 |
| 6 | p | set_3 | 0.00 | 0.12 | 1187.8 | 75.9 | 1.2 | 2 |
| 6 | t | set_1 | 0.00 | 0.28 | 1191.5 | 79.6 | 2.0 | 4 |
| 6 | t | set_2 | 0.00 | 0.28 | 1191.5 | 79.6 | 2.0 | 4 |
| 6 | t | set_3 | 0.00 | 0.28 | 1191.5 | 79.6 | 2.0 | 4 |
| 6 | h | set_1 | 0.00 | 0.08 | 1143.6 | 31.7 | 5.0 | 5 |
| 6 | h | set_2 | 0.00 | 0.08 | 1143.6 | 31.7 | 5.1 | 5 |
| 6 | h | set_3 | 0.00 | 0.08 | 1150.8 | 38.9 | 1.3 | 5 |
| 6 | lq | set_1 | 0.00 | 0.08 | 1138.1 | 26.2 | 7.9 | 5 |
| 6 | lq | set_2 | 0.00 | 0.08 | 1135.8 | 23.8 | 2.6 | 4 |
| 6 | lq | set_3 | 0.00 | 0.08 | 1135.8 | 23.8 | 2.6 | 4 |
| 6 | lp | set_1 | 0.00 | 0.16 | 1180.5 | 68.6 | 5.0 | 4 |
| 6 | lp | set_2 | 0.00 | 0.16 | 1180.9 | 68.9 | 4.2 | 3 |
| 6 | lp | set_3 | 0.00 | 0.12 | 1185.7 | 73.8 | 3.8 | 3 |
| 6 | lt | set_1 | 0.00 | 0.16 | 1170.7 | 58.8 | 6.8 | 8 |
| 6 | lt | set_2 | 0.00 | 0.16 | 1169.6 | 57.7 | 1.2 | 7 |
| 6 | lt | set_3 | 0.00 | 0.16 | 1167.9 | 56.0 | 2.8 | 6 |
| 6 | lh | set_1 | 0.00 | 0.12 | 1159.5 | 47.6 | 1.9 | 8 |
| 6 | lh | set_2 | 0.00 | 0.08 | 1156.7 | 44.8 | 7.8 | 7 |
| 6 | lh | set_3 | 0.00 | 0.08 | 1160.6 | 48.7 | 1.0 | 8 |
| 6 | qp | set_1 | 0.00 | 0.12 | 1152.1 | 40.2 | 7.6 | 5 |
| 6 | qp | set_2 | 0.00 | 0.12 | 1152.1 | 40.2 | 7.6 | 5 |
| 6 | qp | set_3 | 0.00 | 0.12 | 1150.5 | 38.6 | 1.7 | 4 |
| 6 | qt | set_1 | 0.00 | 0.32 | 1157.8 | 45.9 | 4.5 | 4 |
| 6 | qt | set_2 | 0.00 | 0.32 | 1157.8 | 45.9 | 4.5 | 4 |
| 6 | qt | set_3 | 0.00 | 0.32 | 1157.8 | 45.9 | 4.5 | 4 |
| 6 | qh | set_1 | 0.00 | 0.12 | 1138.9 | 27.0 | 5.8 | 4 |
| 6 | qh | set_2 | 0.00 | 0.12 | 1138.9 | 27.0 | 5.8 | 4 |
| 6 | qh | set_3 | 0.00 | 0.12 | 1138.9 | 27.0 | 5.9 | 4 |
| 6 | pt | set_1 | 0.00 | 0.16 | 1164.7 | 52.8 | 1.4 | 4 |
| 6 | pt | set_2 | 0.00 | 0.16 | 1164.7 | 52.8 | 1.4 | 4 |
| 6 | pt | set_3 | 0.00 | 0.12 | 1167.2 | 55.3 | 4.2 | 3 |
| 6 | ph | set_1 | 0.00 | 0.12 | 1144.7 | 32.8 | 3.3 | 4 |
| 6 | ph | set_2 | 0.00 | 0.12 | 1144.7 | 32.8 | 3.3 | 4 |
| 6 | ph | set_3 | 0.00 | 0.12 | 1152.7 | 40.7 | 6.2 | 4 |
| 6 | th | set_1 | 0.00 | 0.08 | 1143.6 | 31.7 | 5.7 | 5 |
| 6 | th | set_2 | 0.00 | 0.08 | 1143.6 | 31.7 | 5.7 | 5 |
| 6 | th | set_3 | 0.00 | 0.08 | 1153.7 | 41.7 | 3.8 | 6 |
| 6 | lqp | set_1 | 0.00 | 0.12 | 1151.7 | 39.8 | 1.0 | 5 |
| 6 | lqp | set_2 | 0.00 | 0.12 | 1151.7 | 39.8 | 1.0 | 5 |
| 6 | lqp | set_3 | 0.00 | 0.12 | 1150.3 | 38.3 | 2.1 | 4 |
| 6 | lqt | set_1 | 0.00 | 0.08 | 1138.1 | 26.2 | 9.1 | 5 |
| 6 | lqt | set_2 | 0.00 | 0.08 | 1135.8 | 23.8 | 3.0 | 4 |
| 6 | lqt | set_3 | 0.00 | 0.08 | 1135.8 | 23.8 | 3.0 | 4 |
| 6 | lqh | set_1 | 0.00 | 0.08 | 1138.2 | 26.2 | 9.2 | 5 |
| 6 | lqh | set_2 | 0.00 | 0.08 | 1135.8 | 23.8 | 3.0 | 4 |
| 6 | lqh | set_3 | 0.00 | 0.08 | 1135.8 | 23.8 | 3.1 | 4 |
| 6 | lpt | set_1 | 0.00 | 0.16 | 1163.5 | 51.5 | 2.9 | 4 |
| 6 | lpt | set_2 | 0.00 | 0.16 | 1163.5 | 51.5 | 3.0 | 4 |
| 6 | lpt | set_3 | 0.00 | 0.12 | 1164.7 | 52.8 | 1.6 | 4 |
| 6 | lph | set_1 | 0.00 | 0.12 | 1146.3 | 34.4 | 1.6 | 4 |
| 6 | lph | set_2 | 0.00 | 0.12 | 1146.3 | 34.3 | 1.6 | 4 |
| 6 | lph | set_3 | 0.00 | 0.08 | 1153.3 | 41.4 | 4.8 | 4 |
| 6 | qpt | set_1 | 0.00 | 0.12 | 1152.1 | 40.2 | 8.8 | 5 |
| 6 | qpt | set_2 | 0.00 | 0.12 | 1152.1 | 40.2 | 8.8 | 5 |
| 6 | qpt | set_3 | 0.00 | 0.12 | 1150.5 | 38.6 | 1.9 | 4 |
| 6 | qph | set_1 | 0.00 | 0.12 | 1148.1 | 36.2 | 6.7 | 6 |
| 6 | qph | set_2 | 0.00 | 0.12 | 1148.1 | 36.2 | 6.8 | 6 |
| 6 | qph | set_3 | 0.00 | 0.08 | 1147.4 | 35.5 | 9.5 | 5 |
| 6 | qth | set_1 | 0.00 | 0.12 | 1138.9 | 27.0 | 6.8 | 4 |
| 6 | qth | set_2 | 0.00 | 0.12 | 1138.9 | 27.0 | 6.8 | 4 |
| 6 | qth | set_3 | 0.00 | 0.12 | 1138.9 | 27.0 | 6.8 | 4 |
| 6 | pth | set_1 | 0.00 | 0.12 | 1144.7 | 32.8 | 3.8 | 4 |
| 6 | pth | set_2 | 0.00 | 0.12 | 1144.7 | 32.8 | 3.8 | 4 |
| 6 | pth | set_3 | 0.00 | 0.12 | 1150.3 | 38.4 | 2.3 | 3 |
| 6 | lqpt | set_1 | 0.00 | 0.12 | 1151.7 | 39.8 | 1.1 | 5 |
| 6 | lqpt | set_2 | 0.00 | 0.12 | 1151.7 | 39.7 | 1.2 | 5 |
| 6 | lqpt | set_3 | 0.00 | 0.12 | 1150.3 | 38.3 | 2.4 | 4 |
| 6 | lqph | set_1 | 0.00 | 0.12 | 1148.6 | 36.6 | 5.6 | 6 |
| 6 | lqph | set_2 | 0.00 | 0.12 | 1148.6 | 36.7 | 5.6 | 6 |
| 6 | lqph | set_3 | 0.00 | 0.08 | 1147.0 | 35.0 | 1.2 | 5 |
| 6 | lqth | set_1 | 0.00 | 0.08 | 1138.2 | 26.2 | 1.0 | 5 |
| 6 | lqth | set_2 | 0.00 | 0.08 | 1135.8 | 23.8 | 3.5 | 4 |
| 6 | lqth | set_3 | 0.00 | 0.08 | 1135.8 | 23.8 | 3.5 | 4 |
| 6 | lpth | set_1 | 0.00 | 0.12 | 1146.3 | 34.3 | 1.8 | 4 |
| 6 | lpth | set_2 | 0.00 | 0.12 | 1148.8 | 36.9 | 5.2 | 5 |
| 6 | lpth | set_3 | 0.00 | 0.08 | 1151.0 | 39.1 | 1.7 | 3 |
| 6 | lqpth | set_1 | 0.00 | 0.12 | 1148.6 | 36.6 | 5.9 | 6 |
| 6 | lqpth | set_2 | 0.00 | 0.12 | 1148.6 | 36.7 | 5.9 | 6 |
| 6 | lqpth | set_3 | 0.00 | 0.08 | 1144.4 | 32.5 | 4.8 | 4 |
| 8 | l | set_1 | 0.00 | 0.16 | 1183.4 | 71.5 | 1.6 | 5 |
| 8 | l | set_2 | 0.00 | 0.16 | 1181.7 | 69.8 | 3.8 | 4 |
| 8 | l | set_3 | 0.00 | 0.16 | 1183.1 | 71.2 | 1.9 | 3 |
| 8 | q | set_1 | 0.00 | 0.32 | 1188.2 | 76.3 | 1.5 | 4 |
| 8 | q | set_2 | 0.00 | 0.32 | 1194.5 | 82.6 | 6.5 | 4 |
| 8 | q | set_3 | 0.00 | 0.32 | 1192.5 | 80.5 | 1.8 | 3 |
| 8 | p | set_1 | 0.00 | 0.12 | 1184.8 | 72.9 | 8.5 | 3 |
| 8 | p | set_2 | 0.00 | 0.12 | 1184.8 | 72.9 | 8.6 | 3 |
| 8 | p | set_3 | 0.00 | 0.12 | 1189.4 | 77.5 | 8.4 | 2 |
| 8 | t | set_1 | 0.00 | 0.08 | 1200.5 | 88.6 | 3.2 | 2 |
| 8 | t | set_2 | 0.00 | 0.08 | 1200.5 | 88.6 | 3.3 | 2 |
| 8 | t | set_3 | 0.00 | 0.08 | 1200.5 | 88.6 | 3.3 | 2 |
| 8 | h | set_1 | 0.00 | 0.08 | 1153.5 | 41.6 | 5.4 | 5 |
| 8 | h | set_2 | 0.00 | 0.08 | 1153.5 | 41.6 | 5.4 | 5 |
| 8 | h | set_3 | 0.00 | 0.08 | 1159.5 | 47.5 | 2.8 | 5 |
| 8 | lq | set_1 | 0.00 | 0.08 | 1140.3 | 28.4 | 4.0 | 4 |
| 8 | lq | set_2 | 0.00 | 0.08 | 1140.3 | 28.4 | 4.0 | 4 |
| 8 | lq | set_3 | 0.00 | 0.08 | 1140.3 | 28.4 | 4.0 | 4 |
| 8 | lp | set_1 | 0.00 | 0.16 | 1183.6 | 71.7 | 1.6 | 3 |
| 8 | lp | set_2 | 0.00 | 0.16 | 1183.6 | 71.7 | 1.6 | 3 |
| 8 | lp | set_3 | 0.00 | 0.12 | 1188.4 | 76.5 | 1.5 | 3 |
| 8 | lt | set_1 | 0.00 | 0.16 | 1185.8 | 73.9 | 5.6 | 6 |
| 8 | lt | set_2 | 0.00 | 0.16 | 1183.9 | 72.0 | 1.4 | 5 |
| 8 | lt | set_3 | 0.00 | 0.16 | 1182.7 | 70.7 | 2.7 | 4 |
| 8 | lh | set_1 | 0.00 | 0.16 | 1167.9 | 56.0 | 4.4 | 6 |
| 8 | lh | set_2 | 0.00 | 0.16 | 1167.9 | 56.0 | 4.4 | 6 |
| 8 | lh | set_3 | 0.00 | 0.16 | 1169.6 | 57.7 | 1.9 | 6 |
| 8 | qp | set_1 | 0.00 | 0.12 | 1160.3 | 48.3 | 2.0 | 4 |
| 8 | qp | set_2 | 0.00 | 0.12 | 1160.3 | 48.3 | 2.0 | 4 |
| 8 | qp | set_3 | 0.00 | 0.12 | 1161.4 | 49.5 | 1.1 | 4 |
| 8 | qt | set_1 | 0.00 | 0.32 | 1167.0 | 55.1 | 7.1 | 4 |
| 8 | qt | set_2 | 0.00 | 0.32 | 1167.0 | 55.1 | 7.2 | 4 |
| 8 | qt | set_3 | 0.00 | 0.32 | 1167.0 | 55.1 | 7.2 | 4 |
| 8 | qh | set_1 | 0.00 | 0.12 | 1145.3 | 33.4 | 3.8 | 4 |
| 8 | qh | set_2 | 0.00 | 0.16 | 1145.3 | 33.4 | 3.8 | 4 |
| 8 | qh | set_3 | 0.00 | 0.16 | 1145.3 | 33.4 | 3.8 | 4 |
| 8 | pt | set_1 | 0.00 | 0.12 | 1175.6 | 63.6 | 1.0 | 4 |
| 8 | pt | set_2 | 0.00 | 0.12 | 1175.6 | 63.6 | 1.0 | 4 |
| 8 | pt | set_3 | 0.00 | 0.12 | 1176.1 | 64.2 | 7.9 | 3 |
| 8 | ph | set_1 | 0.00 | 0.12 | 1154.2 | 42.3 | 4.6 | 3 |
| 8 | ph | set_2 | 0.00 | 0.12 | 1154.2 | 42.3 | 4.6 | 3 |
| 8 | ph | set_3 | 0.00 | 0.12 | 1159.8 | 47.9 | 2.8 | 3 |
| 8 | th | set_1 | 0.00 | 0.08 | 1151.1 | 39.2 | 2.1 | 4 |
| 8 | th | set_2 | 0.00 | 0.08 | 1151.1 | 39.2 | 2.1 | 4 |
| 8 | th | set_3 | 0.00 | 0.08 | 1159.7 | 47.7 | 3.1 | 5 |
| 8 | lqp | set_1 | 0.00 | 0.12 | 1159.7 | 47.8 | 3.0 | 4 |
| 8 | lqp | set_2 | 0.00 | 0.12 | 1159.7 | 47.8 | 3.0 | 4 |
| 8 | lqp | set_3 | 0.00 | 0.12 | 1161.1 | 49.1 | 1.5 | 4 |
| 8 | lqt | set_1 | 0.00 | 0.08 | 1140.3 | 28.4 | 5.0 | 4 |
| 8 | lqt | set_2 | 0.00 | 0.08 | 1140.3 | 28.4 | 5.0 | 4 |
| 8 | lqt | set_3 | 0.00 | 0.08 | 1140.3 | 28.4 | 5.0 | 4 |
| 8 | lqh | set_1 | 0.00 | 0.08 | 1140.3 | 28.4 | 5.1 | 4 |
| 8 | lqh | set_2 | 0.00 | 0.08 | 1140.3 | 28.4 | 5.1 | 4 |
| 8 | lqh | set_3 | 0.00 | 0.08 | 1140.3 | 28.4 | 5.2 | 4 |
| 8 | lpt | set_1 | 0.00 | 0.16 | 1173.9 | 62.0 | 2.7 | 4 |
| 8 | lpt | set_2 | 0.00 | 0.12 | 1173.9 | 62.0 | 2.7 | 4 |
| 8 | lpt | set_3 | 0.00 | 0.12 | 1175.7 | 63.8 | 1.1 | 4 |
| 8 | lph | set_1 | 0.00 | 0.12 | 1153.8 | 41.9 | 6.4 | 3 |
| 8 | lph | set_2 | 0.00 | 0.12 | 1153.8 | 41.9 | 6.5 | 3 |
| 8 | lph | set_3 | 0.00 | 0.12 | 1156.6 | 44.7 | 1.6 | 3 |
| 8 | qpt | set_1 | 0.00 | 0.12 | 1160.3 | 48.3 | 2.6 | 4 |
| 8 | qpt | set_2 | 0.00 | 0.12 | 1160.3 | 48.3 | 2.6 | 4 |
| 8 | qpt | set_3 | 0.00 | 0.12 | 1161.4 | 49.5 | 1.4 | 4 |
| 8 | qph | set_1 | 0.00 | 0.12 | 1156.0 | 44.1 | 2.2 | 5 |
| 8 | qph | set_2 | 0.00 | 0.12 | 1156.0 | 44.1 | 2.2 | 5 |
| 8 | qph | set_3 | 0.00 | 0.12 | 1157.1 | 45.2 | 1.3 | 5 |
| 8 | qth | set_1 | 0.00 | 0.12 | 1145.3 | 33.4 | 4.8 | 4 |
| 8 | qth | set_2 | 0.00 | 0.16 | 1145.3 | 33.4 | 4.9 | 4 |
| 8 | qth | set_3 | 0.00 | 0.16 | 1145.3 | 33.4 | 4.9 | 4 |
| 8 | pth | set_1 | 0.00 | 0.12 | 1156.6 | 44.7 | 1.7 | 4 |
| 8 | pth | set_2 | 0.00 | 0.12 | 1156.6 | 44.7 | 1.7 | 4 |
| 8 | pth | set_3 | 0.00 | 0.12 | 1162.3 | 50.4 | 1.0 | 4 |
| 8 | lqpt | set_1 | 0.00 | 0.12 | 1159.7 | 47.8 | 3.7 | 4 |
| 8 | lqpt | set_2 | 0.00 | 0.12 | 1159.7 | 47.8 | 3.8 | 4 |
| 8 | lqpt | set_3 | 0.00 | 0.12 | 1161.1 | 49.1 | 1.9 | 4 |
| 8 | lqph | set_1 | 0.00 | 0.12 | 1155.5 | 43.6 | 3.2 | 5 |
| 8 | lqph | set_2 | 0.00 | 0.12 | 1155.5 | 43.6 | 3.2 | 5 |
| 8 | lqph | set_3 | 0.00 | 0.12 | 1156.5 | 44.6 | 1.9 | 5 |
| 8 | lqth | set_1 | 0.00 | 0.08 | 1140.3 | 28.4 | 6.4 | 4 |
| 8 | lqth | set_2 | 0.00 | 0.08 | 1140.3 | 28.4 | 6.5 | 4 |
| 8 | lqth | set_3 | 0.00 | 0.08 | 1140.3 | 28.4 | 6.5 | 4 |
| 8 | lpth | set_1 | 0.00 | 0.12 | 1153.8 | 41.9 | 8.0 | 3 |
| 8 | lpth | set_2 | 0.00 | 0.12 | 1153.8 | 41.9 | 8.0 | 3 |
| 8 | lpth | set_3 | 0.00 | 0.12 | 1156.6 | 44.7 | 2.0 | 3 |
| 8 | lqpth | set_1 | 0.00 | 0.12 | 1155.4 | 43.5 | 3.6 | 5 |
| 8 | lqpth | set_2 | 0.00 | 0.12 | 1155.4 | 43.5 | 3.6 | 5 |
| 8 | lqpth | set_3 | 0.00 | 0.12 | 1156.5 | 44.6 | 2.1 | 5 |
| 10 | l | set_1 | 0.00 | 0.12 | 1183.1 | 71.2 | 3.6 | 4 |
| 10 | l | set_2 | 0.00 | 0.12 | 1183.1 | 71.2 | 3.7 | 4 |
| 10 | l | set_3 | 0.00 | 0.12 | 1183.8 | 71.9 | 2.6 | 3 |
| 10 | q | set_1 | 0.00 | 0.36 | 1194.5 | 82.5 | 1.2 | 4 |
| 10 | q | set_2 | 0.00 | 0.36 | 1195.3 | 83.3 | 8.8 | 3 |
| 10 | q | set_3 | 0.00 | 0.36 | 1195.3 | 83.3 | 8.9 | 3 |
| 10 | p | set_1 | 0.00 | 0.12 | 1189.2 | 77.3 | 1.8 | 3 |
| 10 | p | set_2 | 0.00 | 0.12 | 1189.2 | 77.3 | 1.8 | 3 |
| 10 | p | set_3 | 0.00 | 0.16 | 1191.5 | 79.5 | 6.1 | 2 |
| 10 | t | set_1 | 0.00 | 0.08 | 1210.9 | 98.9 | 3.8 | 2 |
| 10 | t | set_2 | 0.00 | 0.08 | 1210.9 | 98.9 | 3.8 | 2 |
| 10 | t | set_3 | 0.00 | 0.08 | 1210.9 | 98.9 | 3.9 | 2 |
| 10 | h | set_1 | 0.00 | 0.08 | 1158.8 | 46.9 | 8.0 | 3 |
| 10 | h | set_2 | 0.00 | 0.08 | 1158.8 | 46.9 | 8.1 | 3 |
| 10 | h | set_3 | 0.00 | 0.08 | 1168.3 | 56.4 | 6.9 | 5 |
| 10 | lq | set_1 | 0.00 | 0.08 | 1145.2 | 33.2 | 7.5 | 4 |
| 10 | lq | set_2 | 0.00 | 0.08 | 1145.2 | 33.2 | 7.6 | 4 |
| 10 | lq | set_3 | 0.00 | 0.08 | 1145.2 | 33.2 | 7.7 | 4 |
| 10 | lp | set_1 | 0.00 | 0.12 | 1187.0 | 75.0 | 6.5 | 3 |
| 10 | lp | set_2 | 0.00 | 0.12 | 1187.0 | 75.0 | 6.6 | 3 |
| 10 | lp | set_3 | 0.00 | 0.12 | 1191.7 | 79.8 | 6.2 | 3 |
| 10 | lt | set_1 | 0.00 | 0.12 | 1183.1 | 71.2 | 4.7 | 4 |
| 10 | lt | set_2 | 0.00 | 0.12 | 1183.1 | 71.2 | 4.8 | 4 |
| 10 | lt | set_3 | 0.00 | 0.12 | 1183.8 | 71.9 | 3.4 | 3 |
| 10 | lh | set_1 | 0.00 | 0.12 | 1181.3 | 69.4 | 1.2 | 5 |
| 10 | lh | set_2 | 0.00 | 0.12 | 1181.2 | 69.3 | 1.2 | 5 |
| 10 | lh | set_3 | 0.00 | 0.12 | 1182.6 | 70.7 | 6.5 | 5 |
| 10 | qp | set_1 | 0.00 | 0.12 | 1171.2 | 59.3 | 1.9 | 4 |
| 10 | qp | set_2 | 0.00 | 0.12 | 1171.2 | 59.3 | 1.9 | 4 |
| 10 | qp | set_3 | 0.00 | 0.16 | 1172.8 | 60.9 | 9.2 | 4 |
| 10 | qt | set_1 | 0.00 | 0.36 | 1177.0 | 65.1 | 1.1 | 4 |
| 10 | qt | set_2 | 0.00 | 0.36 | 1177.0 | 65.1 | 1.1 | 4 |
| 10 | qt | set_3 | 0.00 | 0.36 | 1177.0 | 65.1 | 1.1 | 4 |
| 10 | qh | set_1 | 0.00 | 0.28 | 1157.3 | 45.4 | 2.2 | 6 |
| 10 | qh | set_2 | 0.00 | 0.12 | 1157.3 | 45.4 | 2.2 | 6 |
| 10 | qh | set_3 | 0.00 | 0.12 | 1154.7 | 42.8 | 8.3 | 5 |
| 10 | pt | set_1 | 0.00 | 0.12 | 1185.0 | 73.0 | 2.3 | 3 |
| 10 | pt | set_2 | 0.00 | 0.12 | 1185.0 | 73.0 | 2.3 | 3 |
| 10 | pt | set_3 | 0.00 | 0.16 | 1186.1 | 74.2 | 1.3 | 3 |
| 10 | ph | set_1 | 0.00 | 0.12 | 1164.4 | 52.5 | 7.0 | 4 |
| 10 | ph | set_2 | 0.00 | 0.12 | 1164.4 | 52.5 | 7.1 | 4 |
| 10 | ph | set_3 | 0.00 | 0.12 | 1165.7 | 53.8 | 3.9 | 2 |
| 10 | th | set_1 | 0.00 | 0.08 | 1158.8 | 46.9 | 1.2 | 3 |
| 10 | th | set_2 | 0.00 | 0.08 | 1158.8 | 46.9 | 1.2 | 3 |
| 10 | th | set_3 | 0.00 | 0.08 | 1166.0 | 54.1 | 3.4 | 4 |
| 10 | lqp | set_1 | 0.00 | 0.12 | 1170.5 | 58.6 | 3.7 | 4 |
| 10 | lqp | set_2 | 0.00 | 0.12 | 1170.5 | 58.6 | 3.8 | 4 |
| 10 | lqp | set_3 | 0.00 | 0.12 | 1172.2 | 60.3 | 1.6 | 4 |
| 10 | lqt | set_1 | 0.00 | 0.08 | 1145.2 | 33.2 | 1.2 | 4 |
| 10 | lqt | set_2 | 0.00 | 0.08 | 1145.2 | 33.2 | 1.3 | 4 |
| 10 | lqt | set_3 | 0.00 | 0.08 | 1145.2 | 33.2 | 1.3 | 4 |
| 10 | lqh | set_1 | 0.00 | 0.08 | 1145.2 | 33.2 | 1.3 | 4 |
| 10 | lqh | set_2 | 0.00 | 0.08 | 1145.2 | 33.2 | 1.4 | 4 |
| 10 | lqh | set_3 | 0.00 | 0.08 | 1145.2 | 33.2 | 1.4 | 4 |
| 10 | lpt | set_1 | 0.00 | 0.12 | 1185.2 | 73.3 | 2.9 | 4 |
| 10 | lpt | set_2 | 0.00 | 0.12 | 1185.2 | 73.3 | 3.0 | 4 |
| 10 | lpt | set_3 | 0.00 | 0.12 | 1187.6 | 75.7 | 9.2 | 4 |
| 10 | lph | set_1 | 0.00 | 0.12 | 1160.1 | 48.1 | 9.2 | 3 |
| 10 | lph | set_2 | 0.00 | 0.12 | 1162.4 | 50.5 | 2.8 | 4 |
| 10 | lph | set_3 | 0.00 | 0.12 | 1162.9 | 51.0 | 2.3 | 3 |
| 10 | qpt | set_1 | 0.00 | 0.12 | 1171.2 | 59.3 | 3.8 | 4 |
| 10 | qpt | set_2 | 0.00 | 0.12 | 1171.2 | 59.3 | 3.9 | 4 |
| 10 | qpt | set_3 | 0.00 | 0.16 | 1172.8 | 60.9 | 1.8 | 4 |
| 10 | qph | set_1 | 0.00 | 0.12 | 1163.0 | 51.1 | 2.4 | 4 |
| 10 | qph | set_2 | 0.00 | 0.12 | 1163.0 | 51.1 | 2.5 | 4 |
| 10 | qph | set_3 | 0.00 | 0.12 | 1164.8 | 52.9 | 1.0 | 4 |
| 10 | qth | set_1 | 0.00 | 0.28 | 1154.8 | 42.9 | 1.6 | 5 |
| 10 | qth | set_2 | 0.00 | 0.12 | 1154.7 | 42.8 | 1.7 | 5 |
| 10 | qth | set_3 | 0.00 | 0.12 | 1154.7 | 42.8 | 1.8 | 5 |
| 10 | pth | set_1 | 0.00 | 0.12 | 1164.5 | 52.5 | 1.4 | 4 |
| 10 | pth | set_2 | 0.00 | 0.12 | 1164.5 | 52.5 | 1.5 | 4 |
| 10 | pth | set_3 | 0.00 | 0.12 | 1168.1 | 56.1 | 2.6 | 3 |
| 10 | lqpt | set_1 | 0.00 | 0.12 | 1170.5 | 58.6 | 8.0 | 4 |
| 10 | lqpt | set_2 | 0.00 | 0.12 | 1170.5 | 58.6 | 8.4 | 4 |
| 10 | lqpt | set_3 | 0.00 | 0.12 | 1172.2 | 60.3 | 3.7 | 4 |
| 10 | lqph | set_1 | 0.00 | 0.12 | 1162.4 | 50.5 | 5.3 | 4 |
| 10 | lqph | set_2 | 0.00 | 0.12 | 1162.4 | 50.5 | 5.5 | 4 |
| 10 | lqph | set_3 | 0.00 | 0.12 | 1164.1 | 52.2 | 2.5 | 4 |
| 10 | lqth | set_1 | 0.00 | 0.08 | 1145.2 | 33.2 | 3.5 | 4 |
| 10 | lqth | set_2 | 0.00 | 0.08 | 1145.2 | 33.2 | 3.7 | 4 |
| 10 | lqth | set_3 | 0.00 | 0.08 | 1145.2 | 33.2 | 3.9 | 4 |
| 10 | lpth | set_1 | 0.00 | 0.12 | 1162.5 | 50.5 | 7.4 | 4 |
| 10 | lpth | set_2 | 0.00 | 0.12 | 1162.5 | 50.5 | 8.0 | 4 |
| 10 | lpth | set_3 | 0.00 | 0.12 | 1165.4 | 53.5 | 1.9 | 4 |
| 10 | lqpth | set_1 | 0.00 | 0.12 | 1162.5 | 50.6 | 9.1 | 4 |
| 10 | lqpth | set_2 | 0.00 | 0.12 | 1162.5 | 50.6 | 1.0 | 4 |
| 10 | lqpth | set_3 | 0.00 | 0.12 | 1166.5 | 54.6 | 1.4 | 5 |

l: linear, q: quadratic, p: product, t: threshold, h: hinge
